# Supplementary material for: Binary peptide coacervates as an active model for biomolecular condensates
Source: Nat Commun. 2025 Mar 11;16:2407. doi: 10.1038/s41467-025-57772-z (PMC11897134; doi:10.1038/s41467-025-57772-z)
Supplement: Supplementary file 1 — Supplementary Information [file 41467_2025_57772_MOESM1_ESM.pdf]

Supplementary Information

Binary Peptide-Coacervates as an Active Model for Biomolecular Condensates

Shoupeng Cao[1,2,#], Peng Zhou[3,#], Guizhi Shen[3], Tsvetomir Ivanov[2], Xuehai Yan[3], Katharina Landfester[2]*, and Lucas Caire da Silva[2,4]**

[1]College of Polymer Science and Engineering, State Key Laboratory of Polymer Materials Engineering, Sichuan University, Chengdu 610065, PR China

[2]Max Planck Institute for Polymer Research, 55128 Mainz, Germany

[3]State Key Laboratory of Biochemical Engineering, Key Laboratory of Biopharmaceutical Preparation and Delivery, Institute of Process Engineering, Chinese Academy of Science, Beijing 100190, PR China

[4]Department of Chemistry, McGill University, Montreal H3A 0B8, Canada

[#]These authors contributed equally: Shoupeng Cao, Peng Zhou

*e-mail: yanxh@ipe.ac.cn; landfester@mpip-mainz.mpg.de;
lucas.cairedasilva@mcgill.ca

1. Materials

Boc-*L*-phenylalanine (Boc-Phe-OH, 99%, Sigma), *L*-Phenylalanin-methylester-hydrochlorid (98%, Sigma), *L*-glutamic acid (99%, Sigma), N-(tert-Butoxycarbonyl)-*L*-leucine monohydrate (99%, Sigma), N-(tert-Butoxycarbonyl)-*L*-methionine (98.0 %, TCI), *L*-methionine methyl ester (99%, Sigma), isobutylamin (99%, Sigma), thionyl chloride (99.5%, Sigma), L-glutamic acid (99%, Sigma), glycine methyl ester hydrochloride (99%, Sigma), N-(tert-Butoxycarbonyl)-L-leucine monohydrate (98%, Sigma), allyl chloroformate (97%, Sigma), methyl 2-[(2-aminoacetyl)amino]acetate hydrochloride (98%, Aladdin Scientific), N-(tert-Butoxycarbonyl)-*L*-methionine (98%, Aladdin Scientific), L-Phenylalanine methyl ester hydrochloride (98%, Aladdin Scientific), N-(tert-Butoxycarbonyl)-L-phenylalanine (98%, Aladdin Scientific), L-Methionine methyl ester hydrochloride (98%, Aladdin Scientific), glycine methyl ester hydrochloride (98%, Aladdin Scientific),1-hydroxybenzotriazole (HOBt, 97%, Sigma), 2-(1H-benzotriazole-1-yl)-1,1,3,3-tetramethyluronium tetra fluoroborate (HBTU, 89%, Sigma), *N,N*-diisopropylethylamine (DIPEA, 99%, Sigma), pyridine (99.8%, Sigma), $KMnO_4$ (99%, Sigma), sodium thiosulfate (99%, Sigma), hydrogen peroxide (35 wt% solution, Sigma), 4 M hydrogen chloride solution in dioxane (TCI), bromotris(triphenylphosphine)copper(I) ($Cu(PPh_3)_4Br$, 98%, Sigma), 3-azido-7-hydroxycoumarin (98%, TCI), phenylacetylene (98%, Sigma), chloro-(cyclooctadien)-(pentamethylcyclopentadienyl)-ruthenium(II) (Sigma), resorufin sodium salt (Sigma), Rhodamine 110 chloride (>75%,Sigma), Nile Red (98%, Sigma), methylene blue (Sigma), Rhodamine 6G (95%, Sigma), thioflavin T (>65%, Sigma), Zinc (II) Protoporphyrin IX (92%, Sigma), calcein (mixed isomers, Sigma), Bovine serum albumin (BSA, Sigma), albumin from bovine serum FITC conjugate (FITC-BSA, Thermofisher), horseradish peroxidase (HRP, Sigma), glucose oxidase from aspergillus niger (GOX, Sigma), amplex™ red reagent (Thermo Fisher). $CDCl_3$ (99.9% D, Sigma-Aldrich), DMSO-d_6 (99.9% D, Sigma-Aldrich), HEPES buffer solution (1 M, Sigma), phosphate-buffered saline (PBS, Sigma). Grace Bio-Labs reusable CultureWell™ gaskets (Sigma). All the other solvents, chemicals, and salts used were purchased from Sigma Aldrich unless otherwise stated.

2. Instruments

2.1 Nuclear Magnetic Resonance Spectroscopy (NMR): Routine proton nuclear magnetic resonance (^{1}H NMR) measurements were conducted on a Bruker Avance 300 MHz Ultrashield^{TM} spectrometer equipped with a Bruker SampleCase auto-sampler, using $CDCl_3$ or DMSO-d_6 as the solvent and TMS as the internal standard.

2.2 Micro-plate Reader: Catalytic reactions were evaluated via a microplate reader (TECAN, infinite M1000). The reaction progress was monitored via the absorption or fluorescent signal on the microplate reader (TECAN).

2.3 UV-Vis spectroscopy: UV-Vis spectra were characterized by using UV-vis spectroscopy (Agilent Cary 60 instrument).

2.4 Mass-spectrum: the molecular weights of peptide compounds were measured on an Advion Express L CMS Compact Mass Spectrometer. The obtained spectra were analyzed by using Advion CheMS Express software version 5.1.0.2.

2.5 Wild-field fluorescent microscopy imaging: The wild-field fluorescent images were captured with a Leica DMi8 inverted microscope.

2.6 Bright-field microscopy imaging: The bright-field images were captured with the Leica Mateo TL microscope.

2.7 Confocal scanning microscopy imaging: confocal microscopy was performed using the Leica TCS 264 SP5X system.

3. Synthesis procedures

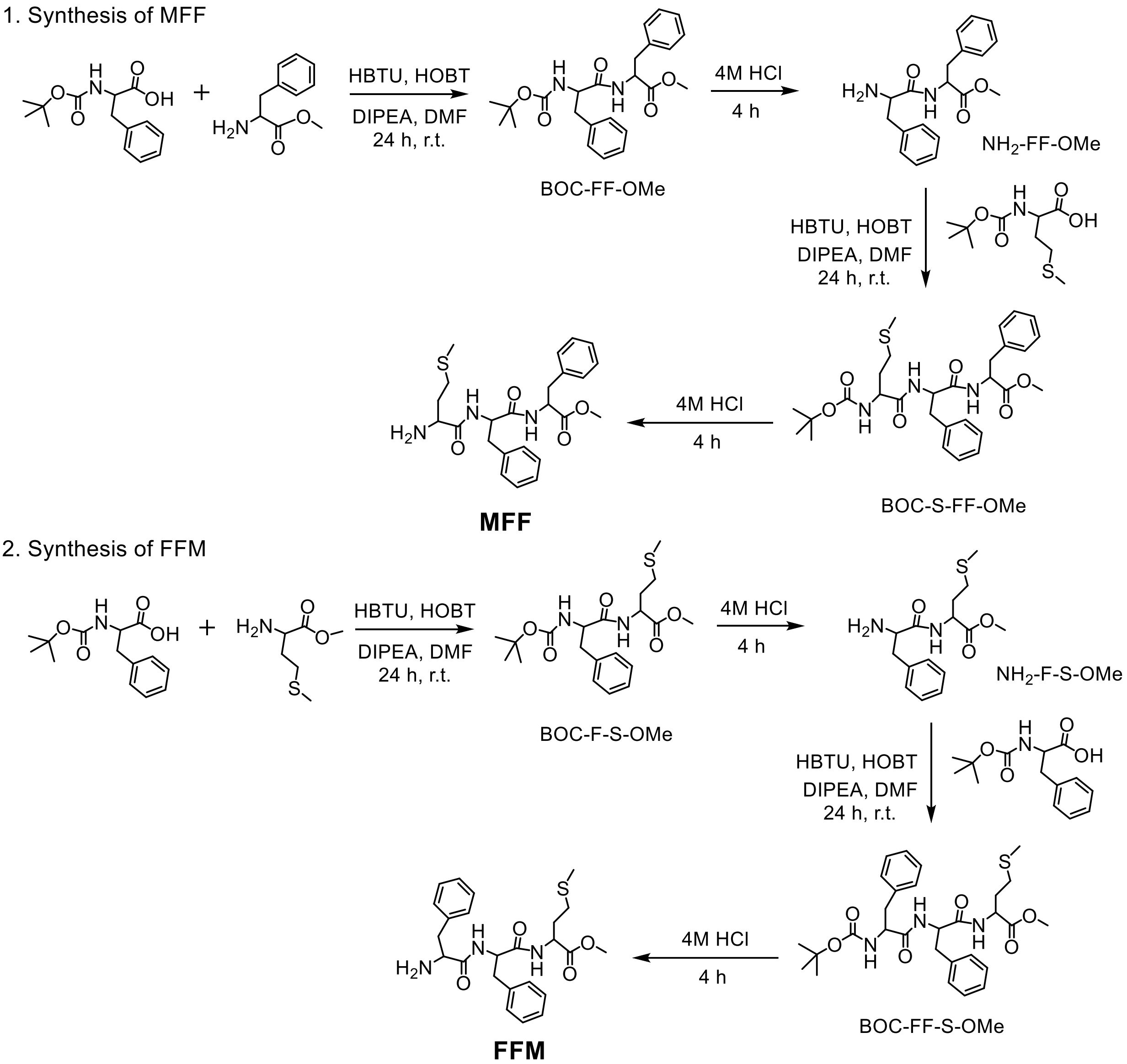

Supplementary Figure 1: Synthesis route towards diphenylalanine-based compounds derivatives, which were synthesized via simple multi-step reactions according to the literature report with slight modifications.[1, 2] This synthesis of **FFM** and **MFF** was typically illustrated in the below, and other diphenylalanine compounds (FFF, FFIba, FFFE-OMe, LF-OMe, MFG, MFM and MGG) were synthesized with similar procedures.

MFF: This compound was synthesized in a four-step reaction.

(**i**) Synthesis of BOC-FF-OMe: First, N-(tert-butoxycarbonyl)-*L*-phenylalanine (Boc-Phe-OH) (2.9 g, 10.9 mmol), HBTU (4.13 g 10.9 mmol) and HOBt (1.47 g, 10.9 mmol) were dissolved in DMF (15 mL) in a round-bottom flask and the mixture was stirred with a magnetic stirrer. N,N-Diisopropylethylamine (DIPEA) (3.5 mL, 21.8 mmol), and L-phenylalanin-methylester-hydrochlorid (2.12 g, 9.9 mmol) were added with an interval of 1 minute respectively and the reaction mixture was stirred for 24 h at room temperature. The reaction mixture was poured

into 500 mL of water. The white precipitate was collected by filtration and washed with water. The crude product was dried in an oven at 40 °C overnight, which produced a 1.7 g white solid characterized by NMR. ^{1}H NMR (300 MHz, $CDCl_3$) δ 7.38 – 6.93 (m, 10H), 6.28 (d, *J* = 7.6 Hz, 1H), 4.94 (s, 1H), 4.80 (q, *J* = 6.3 Hz, 1H), 4.34 (d, *J* = 8.2 Hz, 1H), 3.69 (s, 3H), 3.05 (q, *J* = 6.3 Hz, 4H), 1.42 (s, 9H). ^{13}C NMR (75 MHz, $CDCl_3$) δ 171.33, 170.72, 136.49, 135.63, 129.37, 129.22, 128.68, 128.55, 127.12, 126.99, 77.44, 77.02, 76.60, 53.26, 52.26, 38.27, 37.98, 28.23.

(**ii**) Synthesis of NH_2-FF-OMe: The intermediate compound (1.2 g) was dissolved in 6 mL 4 M hydrogen chloride solution in dioxane for protection. After stirring for 3 h, the solvent was evaporated on a rotary evaporator, yielding oily residue. Diethyl ether was added to the flask and the content was gently stirred. A white precipitate formed and was separated by centrifugation, which produced around 1.0 g of white product. The product was further characterized by NMR. ^{1}H NMR (300 MHz, DMSO) δ 9.26 (d, *J* = 7.5 Hz, 1H), 8.29 (s, 2H), 7.36 – 7.16 (m, 10H), 4.53 (m, 1H), 4.08 (s, 1H), 3.59 (s, 3H), 3.16 (dd, *J* = 14.0, 5.6 Hz, 1H), 3.09 – 2.91 (m, 3H). ^{13}C NMR (75 MHz, DMSO) δ 171.12, 168.15, 136.76, 134.75, 129.59, 129.10, 128.40, 128.33, 127.06, 126.68, 117.68, 53.94, 53.10, 51.97, 36.69, 36.55.

(iii) Synthesis of BOC-S-FF-OMe: N-(tert-Butoxycarbonyl)-L-methionine (Boc-Met-OH) (0.54 g, 2.2 mmol), HBTU (0.83 g 2.2 mmol) and HOBt (0.3 g, 2.2 mmol) were dissolved in DMF (5 mL) in a round-bottom flask and the mixture was stirred with a magnetic stirrer. N,N-Diisopropylethylamine (DIPEA) (0.7 mL, 4.4 mmol), and the product in (ii) (0.72 g, 2 mmol) were added with an interval of 1 minute respectively, and the reaction mixture was stirred for 24 h at room temperature. The reaction mixture was poured into 200 mL of water. A white precipitate was collected by filtration and washed with water. The crude product was dried in an oven at 40 °C overnight, which produced 0.93 g white solid characterized by NMR. ^{1}H NMR (300 MHz, Chloroform-d) δ 7.28 (m, 5H), 7.11 (m, 5H), 6.75 (d, J = 7.8 Hz, 1H), 6.26 (d, J = 7.5 Hz, 1H), 5.21 – 5.03 (m, 1H), 4.86 – 4.58 (m, 2H), 4.22 (d, J = 6.8 Hz, 1H), 3.68 (s, 3H), 3.10 – 2.97 (m, 5H), 2.48 (t, J = 7.2 Hz, 2H), 2.08 (s, 4H), 1.93 (m, 2H), 1.45 (s, 10H). ^{13}C NMR (75 MHz, $CDCl_3$) δ 171.32, 171.23, 170.00, 136.20, 135.59, 129.34, 129.17, 128.70, 128.60, 127.15, 127.12, 77.45, 77.22, 77.02, 76.60, 54.36, 53.43, 52.30, 38.09, 37.84, 31.38, 30.10, 28.30, 15.26.

(iv) Synthesis of MFF: The intermediate compound in (iii) (0.9 g) was dissolved in 10 mL 4 M hydrogen chloride solution in dioxane for protection. After stirring for 3 h, the solvent was evaporated on a rotary evaporator, yielding oily residue. Diethyl ether was added to the flask and the content was gently stirred. A white precipitate formed and was separated by centrifugation, which produced around 0.71 g of white product. The product was further characterized by NMR and GC-Ms. ^{1}H NMR (300 MHz, DMSO) ^{1}H NMR (400 MHz, DMSO-

d_6) δ 8.71 (m, 2H), 8.18 (s, 2H), 7.35 – 7.14 (m, 10H), 4.65 – 4.43 (m, 2H), 3.78 (t, *J* = 6.1 Hz, 1H), 3.59 (s, 3H), 3.16 – 2.92 (m, 4H), 2.87 – 2.66 (m, 2H), 2.45 (m, 2H), 2.03 (s, 3H), 1.96 (m, 2H). ^{13}C NMR (75 MHz, DMSO) δ 171.58, 170.81, 167.95, 137.37, 137.03, 129.15, 128.99, 128.21, 128.10, 126.52, 126.37, 54.07, 53.61, 51.84, 51.42, 37.28, 36.48, 31.10, 27.93, 14.41. MS(EI): calculated for $C_{21}H_{34}N_3O_4S$: 457.20 (M^+); MALDI-TOF found 458.2319 ($M+H^+$).

FFM: This compound was synthesized in a four-step reaction.

(**i**) Synthesis of BOC-F-S-OMe: N-(tert-butoxycarbonyl)-*L*-phenylalanine (Boc-Phe-OH) (1.45 g, 5.5 mmol), HBTU (2.08 g, 5.5 mmol) and HOBt (0.75 g, 5.5 mmol) were dissolved in DMF (10 mL) in a round-bottom flask and the mixture was stirred with a magnetic stirrer. N,N-Diisopropylethylamine (DIPEA) (1.75 mL, 10 mmol), and L-Methionin-methylester-hydrochlorid (1 g, 5 mmol) were added with an interval of 1 minute respectively and the reaction mixture was stirred for 24 h at room temperature. The reaction mixture was poured into 250 mL of water. A white precipitate was collected by filtration and washed with water. The crude product was dried in an oven at 40 ^oC overnight, which produced a 1.26 g white solid characterized by NMR. ^{1}H H NMR (300 MHz, $CDCl_3$) δ 7.42 – 7.14 (m, 5H), 6.56 (d, *J* = 7.7 Hz, 1H), 4.99 (s, 1H), 4.66 (m, 1H), 4.37 (d, *J* = 7.3 Hz, 1H), 3.73 (s, 3H), 3.16 – 3.00 (m, 2H), 2.42 (t, 2H), 2.07 (s, 3H), 1.44 (s, 9H). ^{13}C NMR (75 MHz, $CDCl_3$) δ 171.74, 171.04, 155.35, 136.41, 129.34, 128.71, 127.02, 80.37, 55.79, 52.50, 51.56, 38.06, 31.59, 29.69, 28.25, 15.38.

(**ii**) Synthesis of NH_2-F-S-OMe: The intermediate compound (1.2 g) was dissolved in 9 mL 4 M hydrogen chloride solution in dioxane for protection. After stirring for 3 h, the solvent was evaporated on a rotary evaporator, yielding oily residue. Diethyl ether was added to the flask and the content was gently stirred. A white precipitate formed and was separated by centrifugation, which produced around 1.13 g of white product. The product was further characterized by NMR techniques. ^{1}H NMR (300 MHz, DMSO-d_6) δ 9.10 (d, *J* = 7.6 Hz, 1H), 8.36 – 8.27 (m, 2H), 7.40 – 7.17 (m, 5H), 4.52 – 4.37 (m, 1H), 4.10 (s, 1H), 3.76 – 3.64 (m, 1H), 3.63 (s, 3H), 3.49 (s, 1H), 3.14 (m, 1H), 2.99 (m, 1H), 2.05 (s, 3H), 2.01 – 1.78 (m, 2H). ^{13}C NMR (75 MHz, DMSO) δ 171.46, 168.19, 134.81, 129.53, 128.43, 127.07, 53.18, 52.10, 50.80, 36.67, 30.41, 29.26, 14.40.

(**iii**) Synthesis of BOC-F-F-S-OMe: Then, N-(tert-butoxycarbonyl)-*L*-phenylalanine (Boc-Phe-OH) (0.9 g, 3.3 mmol), HBTU (1.26 g 3.3 mmol) and HOBt (0.45 g, 3.3 mmol) was dissolved in DMF (7 mL) in a round-bottom flask and the mixture was stirred with a magnetic stirrer. N,N-Diisopropylethylamine (DIPEA) (1.05 mL, 6 mmol), and the above intermediate (1.04 g, 3 mmol) were added with an interval of 1 minute respectively and the reaction mixture was stirred for 24 h at room temperature. The reaction mixture was poured into 100 mL of water.

A white precipitate was collected by filtration and washed with water. The crude product was dried in an oven at 40 °C overnight, which produced a 1.5 g white solid characterized by NMR. ^{1}H NMR (300 MHz, Chloroform-*d*) δ 7.38 – 7.05 (m, 10H), 6.72 – 6.32 (m, 2H), 4.84 (d, *J* = 6.6 Hz, 1H), 4.74 – 4.54 (m, 2H), 4.31 (d, *J* = 6.0 Hz, 1H), 3.73 (s, 3H), 3.19 – 2.89 (m, 4H), 2.40 (t, *J* = 7.4 Hz, 2H), 2.07 (s, 4H), 1.91 (m, 1H), 1.36 (s, 9H).

(**iv**) Synthesis of FFM: Then, the above intermediate compound (1.45 g) was dissolved in 10 mL 4 M hydrogen chloride solution in dioxane for protection. After stirring for 3 h, the solvent was evaporated on a rotary evaporator, yielding oily residue. Diethyl ether was added to the flask and the content was gently stirred. A white precipitate formed and was separated by centrifugation, which produced around 1.2 g of white solid. The product was further characterized by NMR and GC-Ms. ^{1}H NMR (400 MHz, DMSO-d_6) δ 8.93 (d, *J* = 8.1 Hz, 1H), 8.67 (d, *J* = 7.7 Hz, 1H), 8.11 (s, 2H), 7.41 – 7.17 (m, 10H), 4.62 (m, 1H), 4.45 (m, 1H), 4.01 (s, 1H), 3.63 (s, 3H), 3.15 (m, 1H), 3.06 (m, 1H), 2.90 (m, 2H), 2.05 (s, 3H), 2.03 – 1.85 (m, 2H). ^{13}C NMR (75 MHz, DMSO) δ 171.92, 170.83, 167.82, 137.40, 134.77, 129.69, 129.25, 128.48, 128.35, 128.11, 126.98, 126.37, 66.31, 54.27, 53.14, 51.96, 50.92, 37.44, 36.63, 30.43, 29.47, 14.50. MS(EI): calculated for $C_{24}H_{31}N_3O_4S$: 457.20 (M^+); MALDI-TOF found 458.2245 ($M+H^+$).

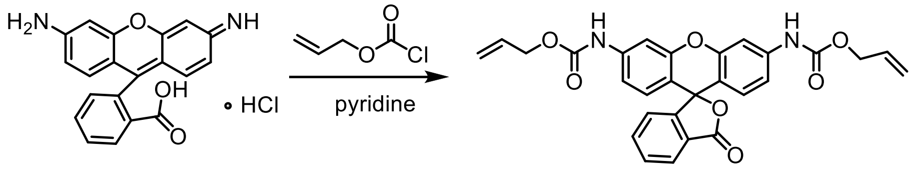

Supplementary Figure 2: synthesis route towards a caged Rhodamine 110

This compound was synthesized according to literature with similar procedures.[3] In details, rhodamine 110 (100 mg, 0.27 mmol) was dissolved in dry DMF (1 mL) in a 10 mL pear-shaped flask equipped with a magnetic stir bar. Pyridine (65 μL, 0.80 mmol) was then added and the mixture was cooled down to 0 °C. Then the flask was purged with nitrogen. Finally, allyl chloroformate (57 μL, 0.53 mmol) was added dropwise to the above solution. The reaction was then allowed to warm to room temperature and stirred overnight. Afterwards, the reaction mixture was diluted with ethyl acetate (20 mL) and the mixture was washed with aqueous hydrochloric acid (5%, 10 mL) twice and with aqueous sodium bicarbonate solution (saturated, 2×10 mL). The organic phase was then dried with sodium sulfate and filtered. The solution was then concentrated with the rotavap and subjected to flash chromatography using 1:1 hexanes/ethyl acetate, which yielded a solid product (40% yield). ^{1}H NMR (400 MHz, DMSO) δ 10.09 (s, 2H), 8.02 (d, *J* = 7.5 Hz, 1H), 7.76 (m, 2H), 7.58 (d, *J* = 2.1 Hz, 2H), 7.28 (d, *J* = 7.7 Hz, 1H), 7.23 – 7.13 (m, 2H), 6.71 (d, *J* = 8.6 Hz, 2H), 5.99 (m, 2H), 5.38 (d, *J* = 17.2 Hz, 2H), 5.26 (d, *J* = 10.5 Hz, 2H), 4.64 (d, *J* = 5.5 Hz, 4H). ^{13}C NMR (101 MHz,

DMSO) δ 169.17, 153.58, 152.98, 151.42, 141.92, 136.21, 133.51, 130.70, 129.00, 126.19, 125.26, 124.44, 118.30, 114.97, 112.94, 105.60, 82.44, 65.44, 40.63, 40.58, 40.43, 40.38, 40.22, 40.17, 40.01, 39.96, 39.80, 39.75, 39.54, 39.33.

RITC/Cy5 labeling of GOX and HRP: Enzyme labeling was realized according to the reported literature with slight modifications.[4] In brief, 10 mg GOX or HRP was dissolved in 2 mL of sodium carbonate buffer (100 mM, pH 8.5). Then 20 μL of FITC/RITC solution (1.0 mg/mL, in DMSO) was added slowly to the above solution. The mixture was stirred at r.t. for 3 h in the dark, and followed by purification with dialyzing (MWCO 12−14 kDa) against Milli-Q water for 2 days and storage at 4°C for further use.

Synthesis of quaternized amylose (Q-AM) and carboxy-functionalized amylose (C-AM): The synthesis of pairs for complex coacervation was accomplished using a literature report with slight modifications.[4] In brief, quaternized amylose (Q-AM) was prepared by dissolving 1.5 g amylose and 2.8 g NaOH in 14 mL Milli-Q at 30°C. After complete dissolution of the amylose, 11.6 mL of 3-chloro-2-hydroxypropyltrimethylammonium chloride solution (60 wt% in water) was added dropwise into the stirring reaction mixture, which was subsequently left to react overnight. After this time, the mixture was neutralized with acetic acid and precipitated into 200 mL cold ethanol. The resulting precipitate was re-dissolved in Milli-Q water and dialyzed extensively against water using regenerated cellulose dialysis tubing (Spectrum Labs, USA) with a 3.5 kDa MWCO before lyophilization. ^{1}H NMR (D_2O) characterization data and chemical structures are presented in Figure S15.

Carboxyl-functionalized amylose (C-AM) was prepared by dissolving 1.5 g amylose and 3.6 g NaOH in 15 mL Milli-Q at 70 °C. After complete dissolution of the amylose, 2.7 g chloroacetic acid was added and the reaction mixture was left to stir for 2 h. After the reaction, the mixture was neutralized with acetic acid and precipitated into 200 mL cold ethanol. The resulting precipitate was re-dissolved in Milli-Q water and dialyzed extensively against water using regenerated cellulose dialysis tubing (Spectrum Labs, USA) with a 3.5 kDa MWCO before lyophilization. ^{1}H NMR (D_2O) characterization data and chemical structures are presented in Figure S15.

Synthesis of BSA@MnO_2 nanoparticles: The synthesis of BSA-stabilized MnO_2 nanoparticles was performed according to the reported literature with slight modifications.[5] In brief, a 4 mL $KMnO_4$ solution (5 mM) was added to a 25 mL round bottom flask, which was purged with N_2 and bubbled for 30 minutes at RT. Then an 8 mL solution of $Na_2S_2O_3$ (1.875 mM) was added at a rate of 1 mL min^{-1} with a syringe pump. The mixture solution was stirred for 10 minutes. Then 3 mL BSA solution (7 mg mL^{-1}) was added dropwise. Then the solution was stirred for another hour, and the solution was dialyzed against MilliQ (Spectrapor,

MWCO: 1000 KDa) to remove excess BSA. Samples were stored in the 4 ^{o}C fridge before use.

RITC labeling of BSA@MnO_2 nanoparticles: In brief, 500 μL of MnO_2 nanoparticle solution was dispersed in PBS buffer (pH 8.5). 20 μL RITC (1 mg mL-1 in DMSO) was added, and the reaction mixture was allowed to stir at R.T. for 3 h. Then the RITC labeled nanoparticles were recovered via dialyzing (MWCO 12-14 KDa) against Milli-Q water for 2 days and stored at 4°C for further use.

4. Peptide droplet formulations and guest cargoes encapsulation

Preparation of peptide coacervates: taking the coacervate droplet formation from FFM/MFF as an example, the FFM and FFM solids were dissolved in 5 mM HEPES buffer (pH~6) with a concentration of 20 mg mL^{-1} after heating. For microscopy imaging the coacervates, 1.25 µL of FFM solution and 1.25 µL of MFF solution were mixed and added with 0.1 M NaOH solution to reach a pH above 7 (within a total of 5 mg mL^{-1} peptide). The solution immediately turned into a milky color. The formation of coacervate droplets was confirmed with a Leica DMi8 inverted microscope.

Turbidity measurement: All turbidity-based titrations were performed on a Tecan multimode plate reader. Turbidity was utilized as the indicator of phase separation of samples, in which the droplet formation was confirmed by optical microscopy. Absorption at 600 nm is used as the wavelength for all turbidity measurements, and all measurements were performed at room temperature. After sample addition and shaking for 5 s, the turbidity value was recorded. A well with the same volume of buffer was used as blank.

Encapsulating of protein and enzyme: All experiments were performed at room temperature unless indicated otherwise. All of the protein and enzyme solutions were kept at -4 ^{o}C or -20 ^{o}C before use. In detail, 10 µL of the prepared coacervate solution (5 mg mL^{-1}) was mixed with 0.5 µL RITC-BSA (0.5 mg mL^{-1}) or Cy5-GOX (0.5 mg mL^{-1}) or RITC-HRP (0.5 mg mL^{-1}) solution, and then the encapsulation was further confirmed with confocal microscopy using the Leica TCS 264 SP5X system.

Partitioning of guest molecules: All experiments were performed at room temperature unless indicated otherwise. In brief, the coacervate droplets solution was first prepared by adding 0.1 M NaOH solution into peptide solution in HEPES buffer. Then 10 µL of the prepared coacervate solution (10 mg mL^{-1}) was mixed with the 0.2 µL dye solutions (2 mg mL^{-1} in DMSO or Milli-Q) by pipetting. Then the mixture was dropped on a glass surface with a cover glass using a home-made setup (Supplementary Figure S16). Then droplets were imaged with confocal microscopy using the Leica TCS 264 SP5X system.

Fluorescence recovery after photobleaching (FRAP): All experiments were performed at room temperature. Briefly, the coacervate droplet solution was first prepared by adding 0.1 M NaOH solution to the peptide solution in HEPES buffer. Then, 20 µL of the prepared coacervate dispersion (5 mg mL^{-1}) was mixed with 0.5 µL rhodamine B solution (0.2 mg mL^{-1} in 10% DMSO in Milli-Q) by pipetting. The mixture was then dropped onto a glass surface with a coverslip using a homemade setup to avoid evaporation after days of incubation. For FRAP measurements, the fluorescence intensities of the peptide coacervates were quenched to 40%-50% of the initial level, and then the recovery of the fluorescence intensity of the droplets was followed using the Leica TCS 264 SP5X system.

5. Biomimicry application with peptide droplets

5.1 Enzymatic metabolism with peptide coacervates: The enzymatic reactions with peptide coacervates were determined with confocal imaging.

Confocal imaging measurements: 20 μL peptide coacervates solution (5 mg mL^{-1}) in a HEPES/NaCl buffer (pH~8) was mixed with 1 μL HRP solution (final conc. 0.0025 mg mL^{-1}). The mixture was firstly treated with 0.2 μL Amplex-red (20 mg mL^{-1}). After that, different concentration of H_2O_2 was added, and the emission intensity at λ_{em}=580 nm was then recorded via confocal imaging.

5.2 Bio-orthogonal click chemistry with peptide coacervates: To a 200 μL peptide-coacervates solution (5 mg mL^{-1}) was added 1 μL $Cu(PPh_3)_4Br$ (10 mg mL^{-1} in DMSO) via pipetting. After equilibrium for 2 min, the mixture was added with 1 μL 3-azido-7-hydroxycoumarin (10 mg mL^{-1} in DMSO) and 1 μL phenylacetylene (10 mg mL^{-1} in DMSO). The solution was mixed via pipetting for seconds. Since the product is hydrophobic, the product should be dissolved with an organic solution to better compare with the control. Thus, after a certain period, aliquot solutions (20 μL) were taken and dissolved with 30 μL acetonitrile. The change in fluorescent intensity (λ_{em}=451 nm) was measured with a microplate reader.

5.4 Bio-orthogonal uncaging reaction with alloc-protected Rhodamine 110 utilizing peptide coacervates: The bio-orthogonal catalytic potential of peptide-coacervates was assessed using the allylcarbamate cleavage of alloc-protected Rhodamine 110 with microplate-reader test and confocal imaging study.

(i) Micro-plate reader measurements: To a 100 μL peptide-coacervates solution (5 mg mL^{-1}) was added 0.5 μL [Cp*Ru(cod)Cl] (Cp* = pentamethylcyclopentadienyl, cod =1,5-cyclooctadiene, abbreviated as Ru, 10 mg mL^{-1} in DMSO) via pipetting. After equilibrium for 2 min, the mixture was added with 0.5 μL caged Rhodamine 110 (10 mg mL^{-1} in DMSO). The solution was mixed via pipetting for seconds. Change of fluorescent intensity (λ_{em}=525 nm) was monitored through a micro-plate reader under periodic shaking.

(ii) *Confocal imaging measurements*: 20 μL peptide coacervates solution (10 mg mL^{-1} in HEPES/NaCl buffer, pH ~8) was mixed with 0.1 μL Ru solution (2 mg mL^{-1} in DMSO). The mixture was then treated with 0.1 μL caged Rhodamine 110 (2 mg mL^{-1} in DMSO). After that, the emission intensity at λ_{em}=525 nm was then recorded via confocal imaging.

6. As sub-organelles inside complex coacervates-based synthetic cells

6.1 Membrane-bound complex coacervates formation: the formation of membrane-less complex coacervates was adapted to the literature report with slight modifications, which utilized the complex coacervation between Q-Am and C-Am, then the membrane-less complex coacervates were stabilized with BSA@MnO_2 nanoparticles.[2, 4] In brief Q-Am and CM-Am was dissolved in PBS buffer with a concentration of 2.5 mg mL^{-1}. Coacervation was induced by mixing the solutions of Q-AM and C-AM in a ratio of 1:1. Then different volumes of BSA@MnO_2 nanoparticles were added to the solution by pipetting. After that, the stability of BSA@MnO2 stabilized complex coacervate was checked via optical microscopy.

6.2 Integration of peptide-coacervates as sub-organelle: 8 μL of peptide-coacervates (10 mg mL^{-1}) was first formed by adding a few amounts of 0.1 M NaOH solution to peptide solution. Then 10 μL of C-AM (2.5 mg mL^{-1} in PBS, pH~9) was added and mixed via pipetting for ~10 s. After that, 12.5 μL of Q-AM (2.5 mg mL^{-1} in PBS, pH~9) was added and mixed via pipetting for ~10 s. Then a solution of 4 μL of BSA@MnO_2 (~0.7 mg mL^{-1}) was added followed by pipetting for ~10 s. The resultant complex was then subject to optical microscopy to observe the multi-compartmentalized structure.

6.3 Bio-orthogonal uncaging reaction in the multi-compartmentalized system: The fabrication procedures are similar to those above, except that before encapsulation into complex coacervates as organelles, the peptide-coacervates were treated with 0.2 μL Ru (2 mg mL^{-1} in DMSO). After the formation of a multi-compartmentalized structure, the mixture was treated with 0.2 μL caged Rhodamine 110 (2 mg mL^{-1} in DMSO). Then the fluorescent emission from the uncaged product was monitored via confocal microscopy.

7. Molecular dynamics simulation

All-atom molecular dynamics (AA-MD) simulations were performed using the Gromacs package (version 2020).[6] The CharmM36 force field was used to model the FFM, MFF, and Hepes molecules,[7] while the water molecules were modeled using the tip3p potential.[8] The simulation system comprised 24 peptide molecules, 48 Hepes molecules, and 5892 water molecules in a box sized 6.0 nm × 6.0 nm × 6.0 nm at the concentration of 80 mg mL^{-1}. In the single-molecule system, there are 24 FFM or MFF molecules and in the mixture system, there are 12 FFM and 12 MFF molecules. For the concentration of 160 mg mL^{-1}, the simulation system comprised 48 peptide molecules, 96 Hepes molecules, and 4741 water molecules. The charge neutrality was maintained by introducing sodium ions. The initial steps involved energy minimization using the steepest-descent method and the conjugate-gradient algorithm until convergence was achieved. The NPT ensemble was used to maintain a temperature of 293 K and a pressure of 1 bar.[9] The Nosé–Hoover thermostat was used with 0.1 ps as the thermostat relaxation time. The isotropic Parrinello-Rahman barostat was used with 1.0 ps as the barostat relaxation time. The bond lengths were constrained using the LINCS algorithm. The electrostatic interactions were computed using the particle mesh Ewald (PME) algorithm with a cutoff of 1.0 nm,[10] while the Lennard-Jones interactions employed a cutoff of 1.0 nm. The production runs in the NPT ensemble were run for at least 60 ns. A dielectric constant of 1 and a time step of 2 fs were applied. The aggregation propensity is defined as the ratio of the solvent-accessible surface area (SASA) of the peptide molecules in the initial state to the SASA of the final configuration of the simulation.[11] The clustering degree of the peptide is defined by the largest cluster size divided by a total number of peptide molecules in the system.[12] The H-bond is defined as the angle between the donor atom and the acceptor atom less than 30 degrees, and the distance between the donor atom and acceptor atom less than 0.35 nm.

8. Supplementary Figures and Tables

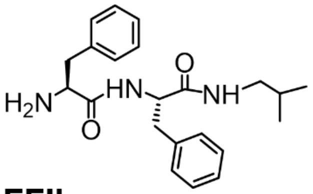

FFIba

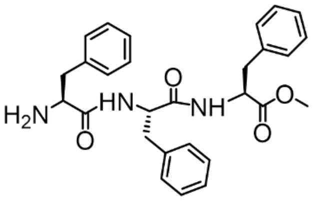

FFF

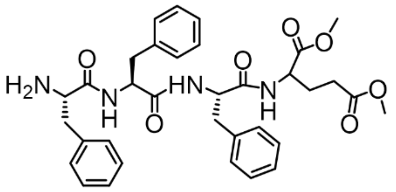

FFFE-OMe

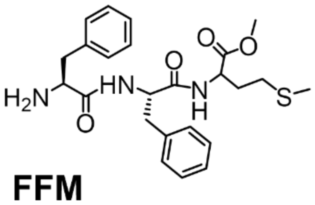

FFM

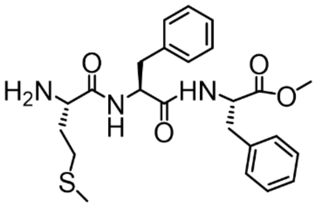

MFF

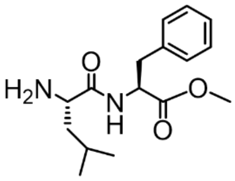

LF

Supplementary Figure 3. Chemical structures of the diphenylalanine precursor and derivatives.

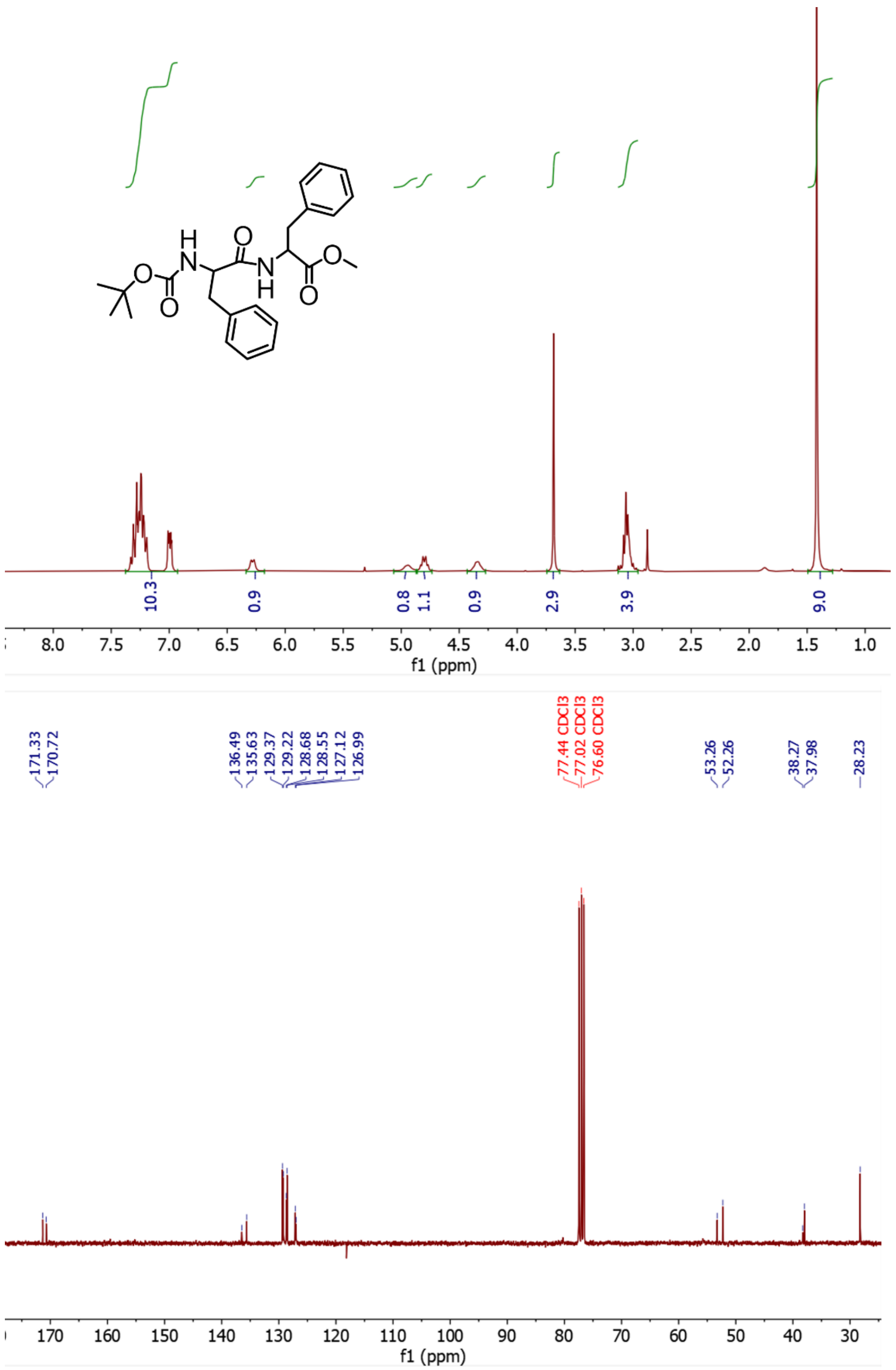

Supplementary Figure 4. ^{1}H NMR and ^{13}C NMR spectra of BOC-FF-OMe in $CDCl_3$.

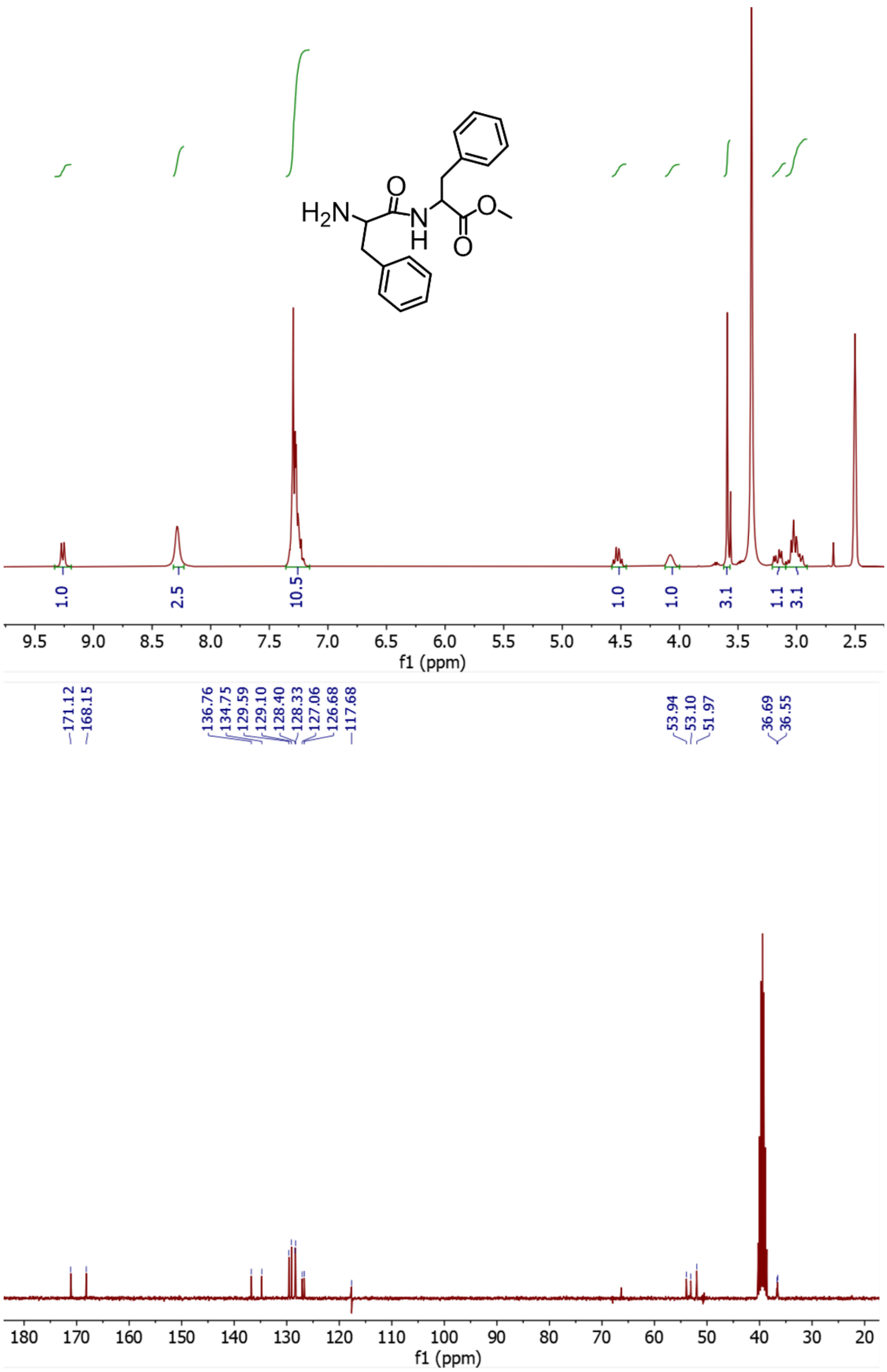

Supplementary Figure 5. ^{1}H NMR and ^{13}C NMR spectra of NH_2-FF-OMe in DMSO.

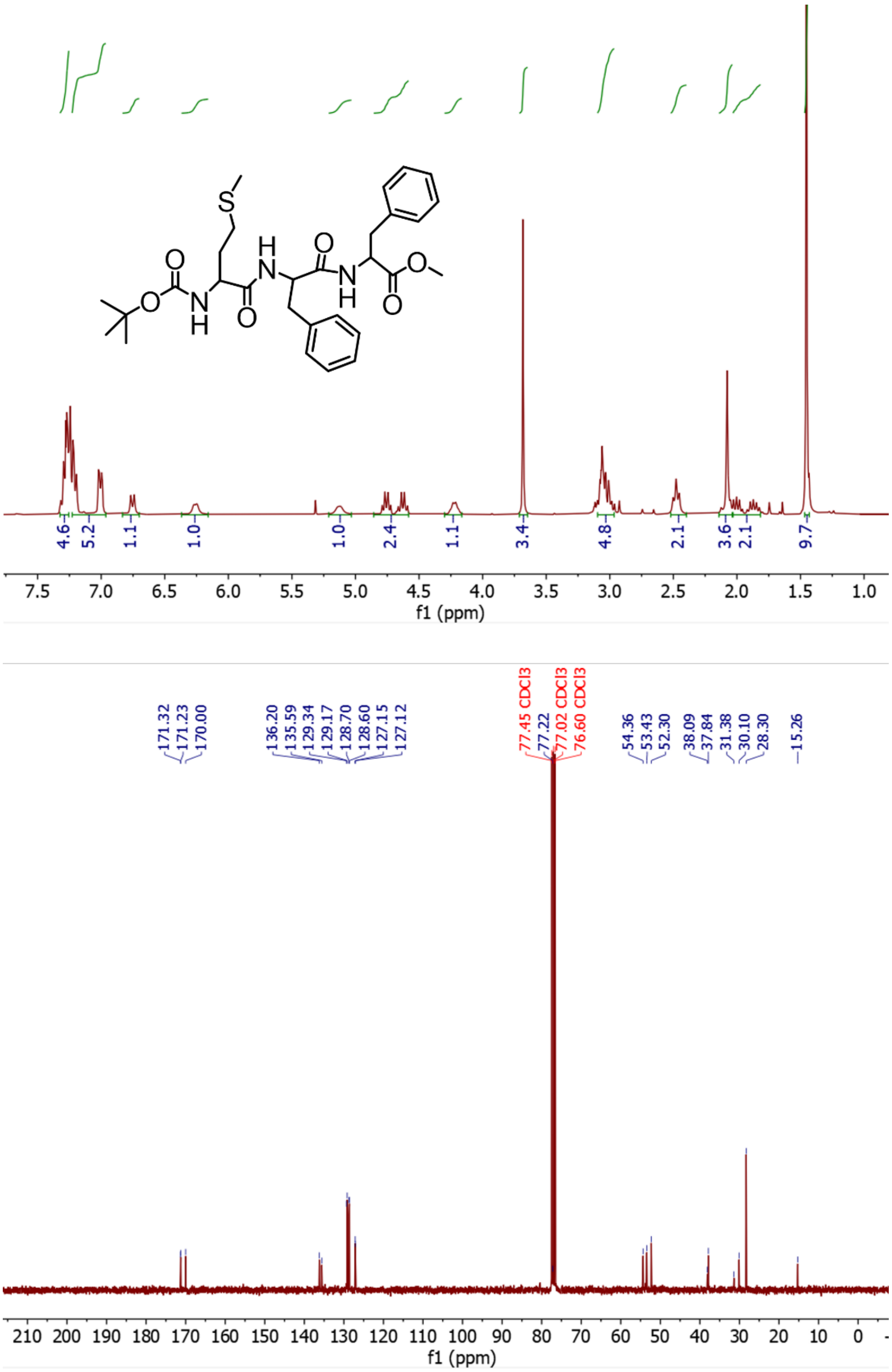

Supplementary Figure 6. ^{1}H NMR and ^{13}C NMR spectra of BOC-S-FF-OMe in $CDCl_3$.

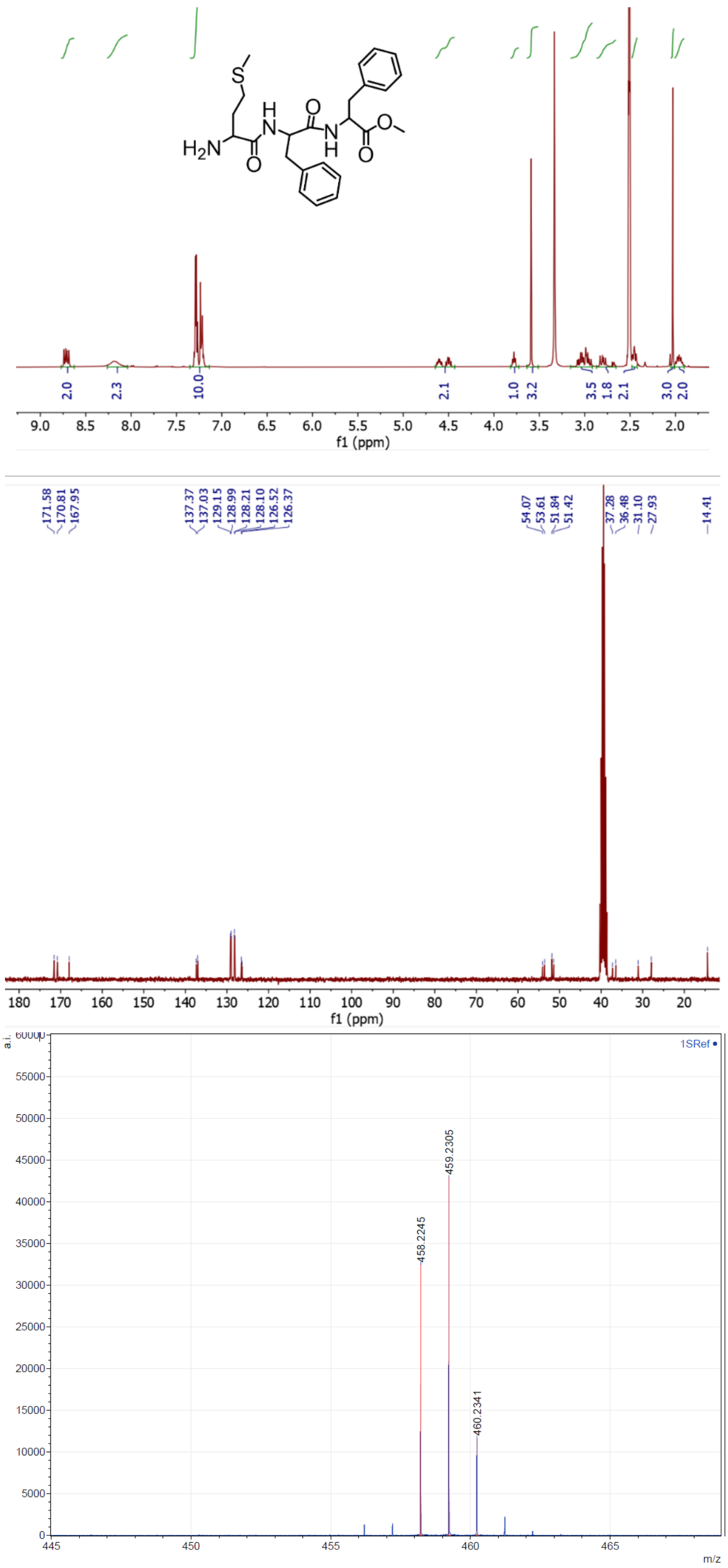

Supplementary Figure 7. ^{1}H NMR, ^{13}C NMR, and MALDI-TOF spectra of MFF (NMRs in DMSO).

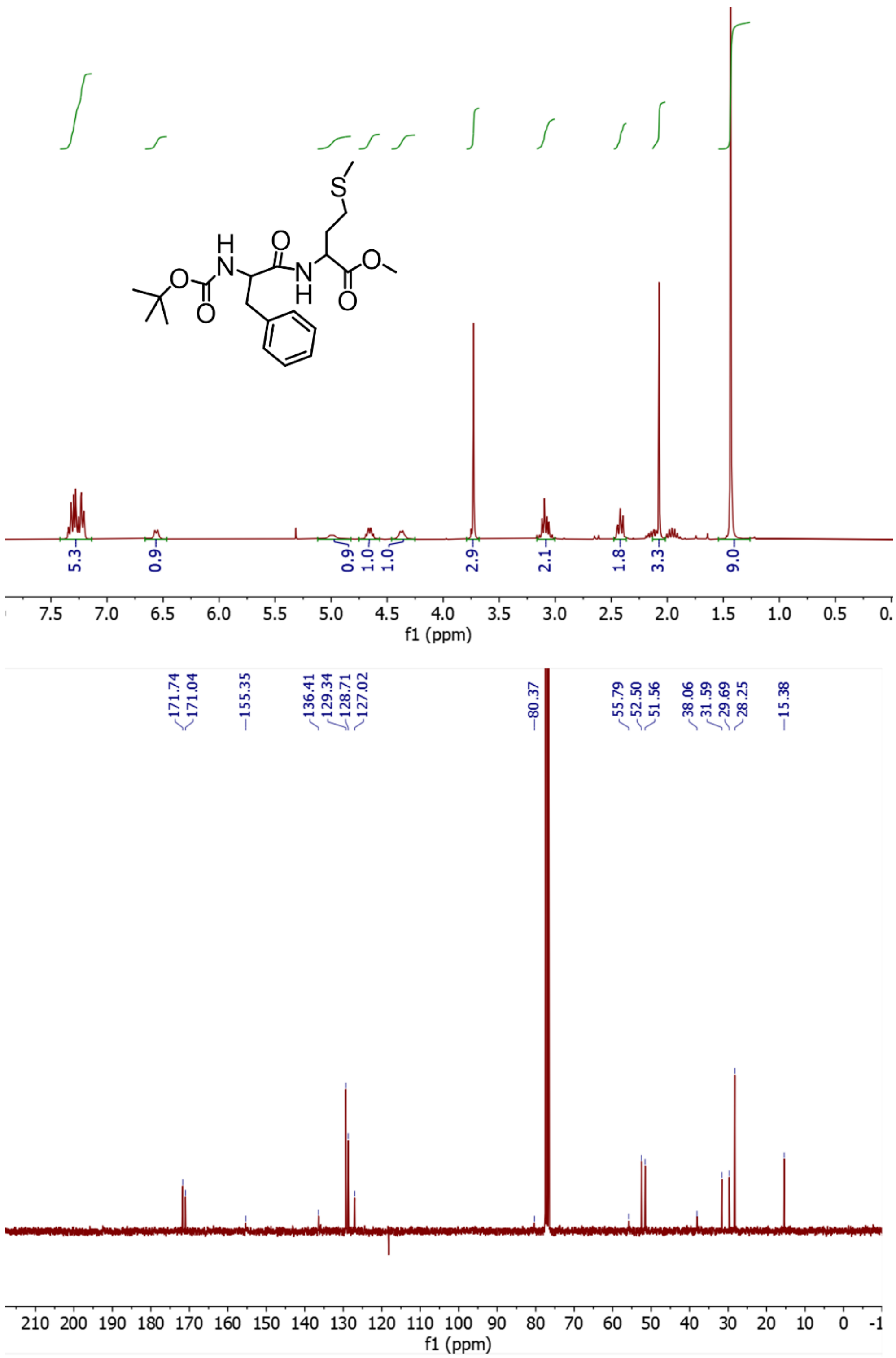

Supplementary Figure 8. ^{1}H NMR and ^{13}C NMR spectra of BOC-F-S-OMe in $CDCl_3$.

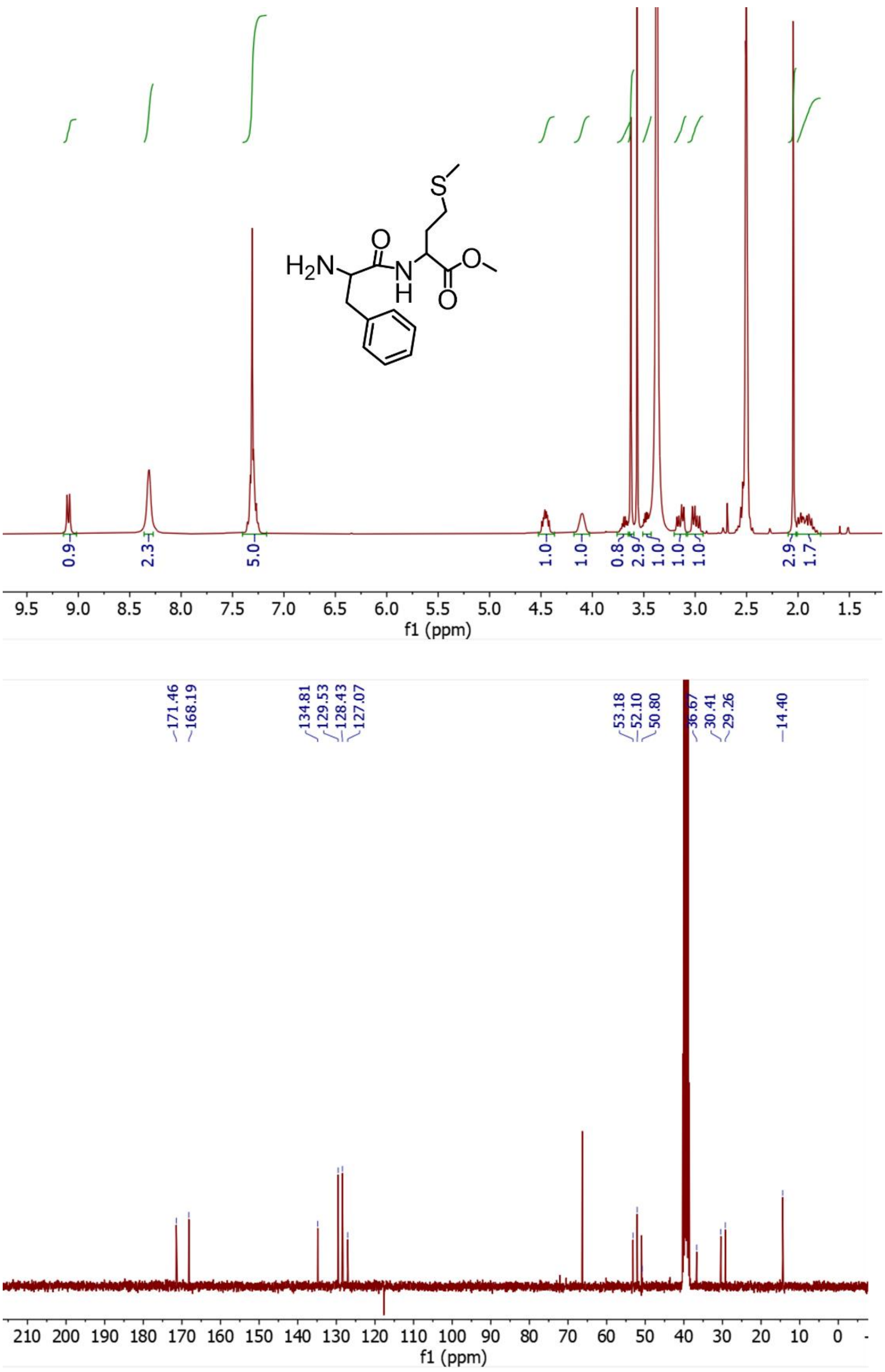

Supplementary Figure 9. ^{1}H NMR and ^{13}C NMR spectra of NH_2-F-S-OMe in DMSO.

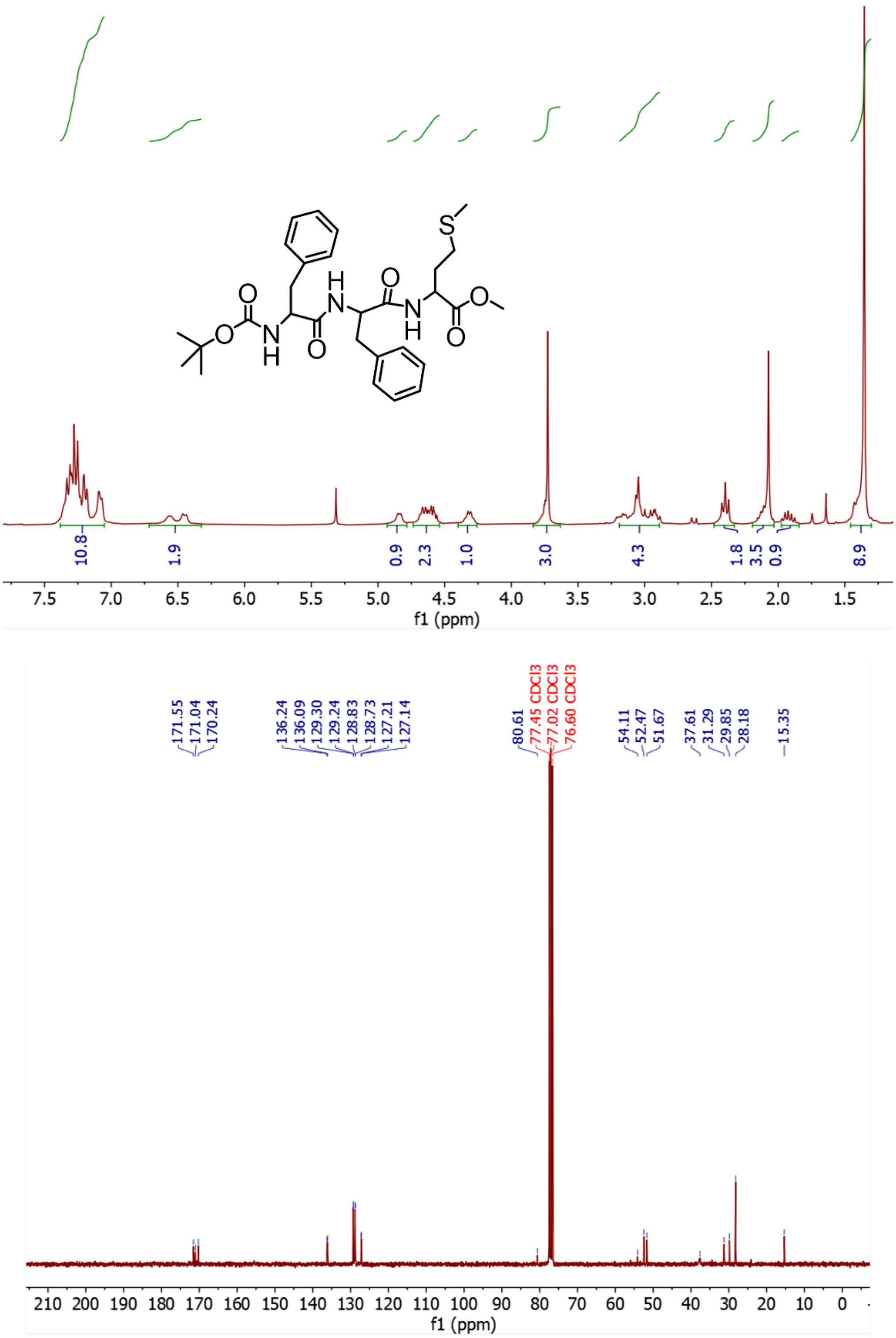

Supplementary Figure 10. ^{1}H NMR and ^{13}C NMR spectra of BOC-F-F-S-OMe in $CDCl_3$.

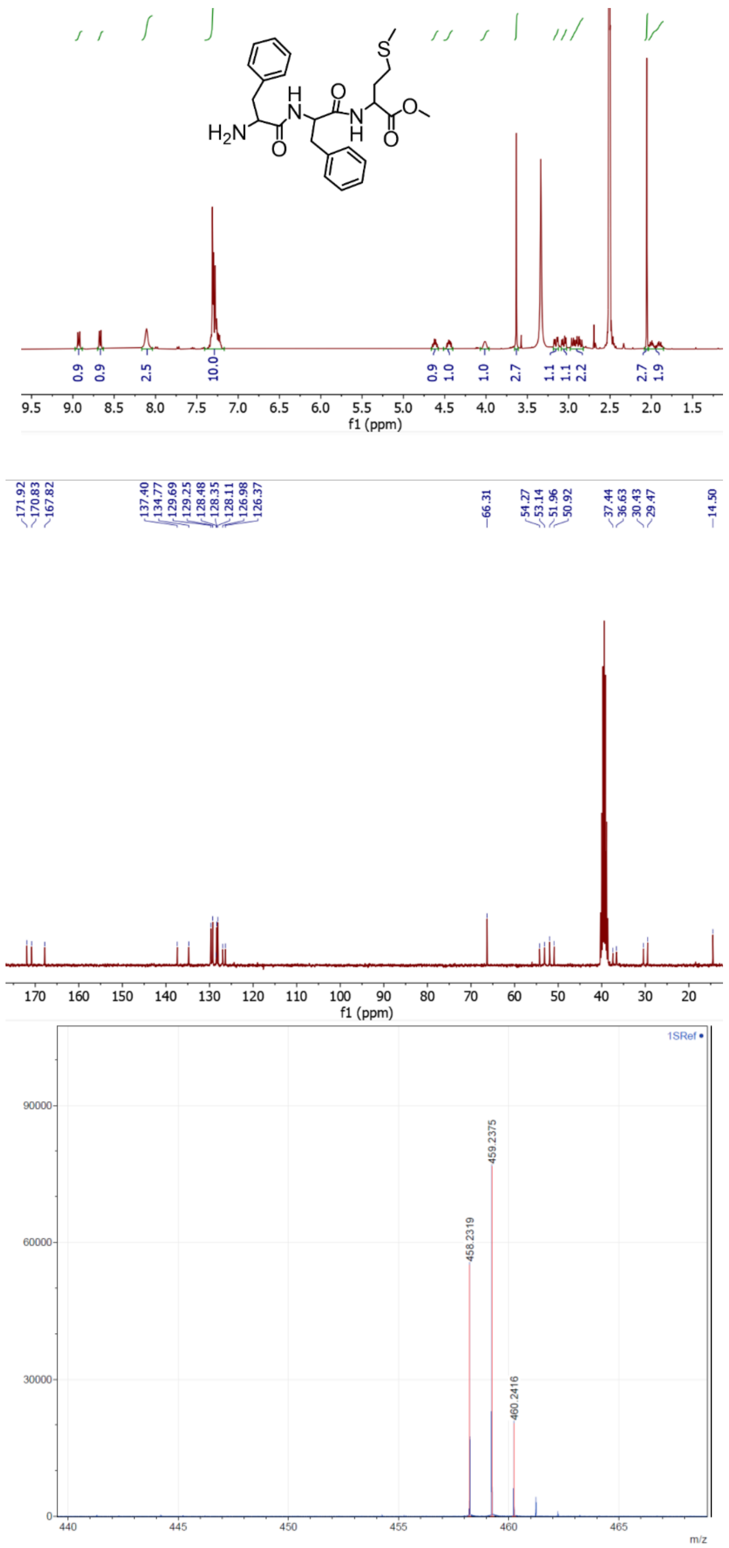

Supplementary Figure 11. ^{1}H NMR, ^{13}C NMR and MALDI-TOF spectra of FFM (NMRs in DMSO)

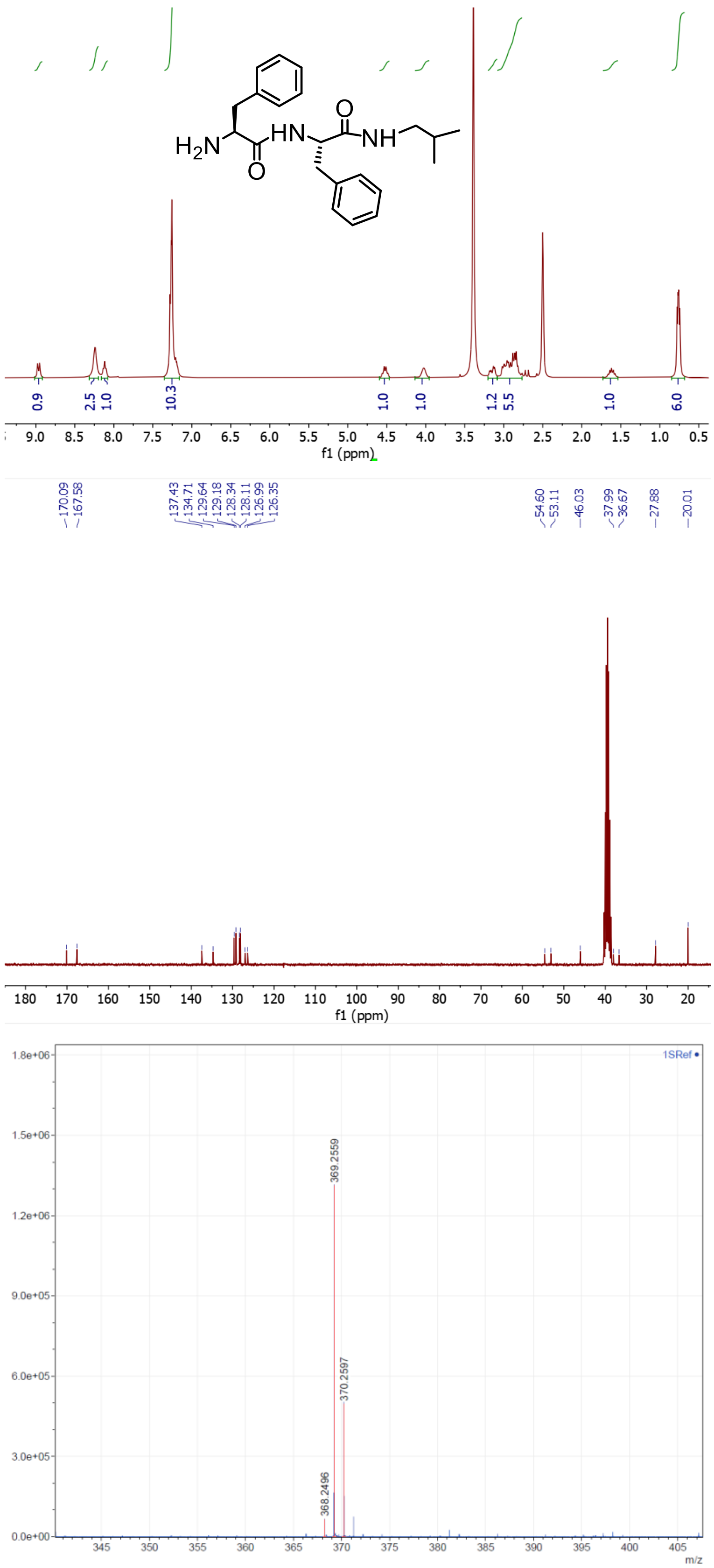

Supplementary Figure 12. ^{1}H NMR, ^{13}C NMR and MALDI-TOF spectra of FFIba (NMRs in DMSO).

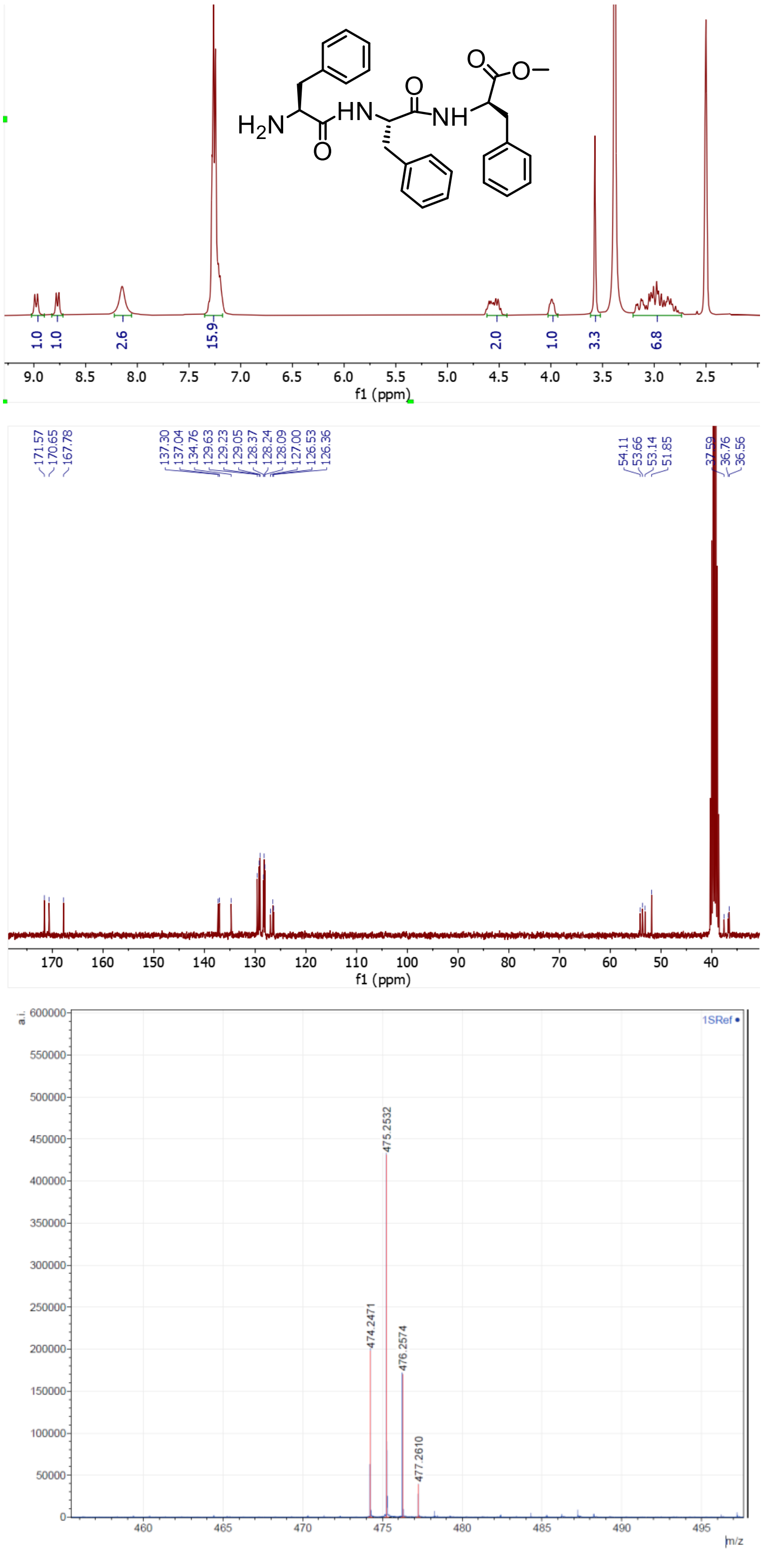

Supplementary Figure 13. ^{1}H NMR, ^{13}C NMR, and MALDI-TOF spectra of FFF (NMRs in DMSO).

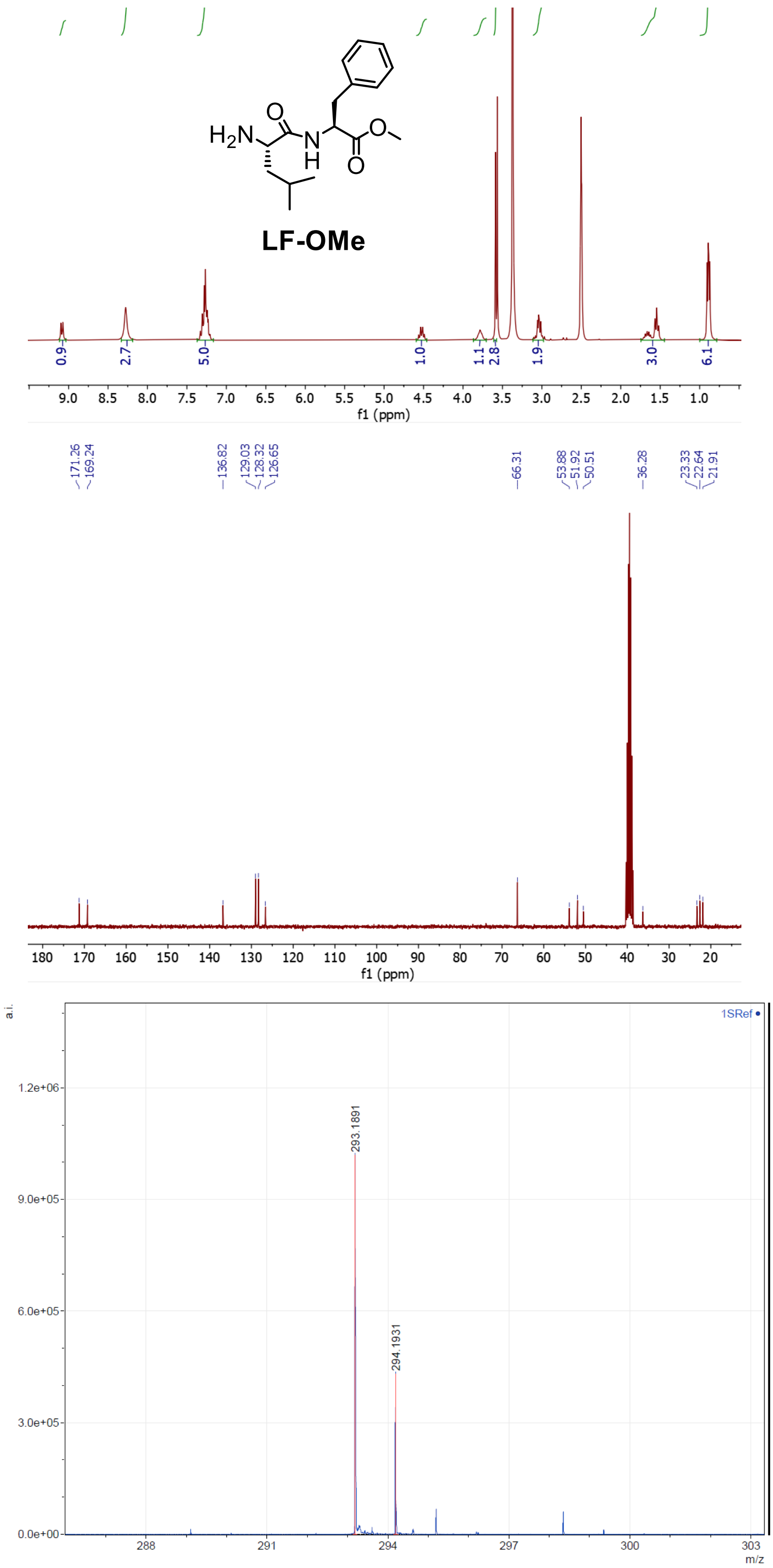

Supplementary Figure 14. ^{1}H NMR, ^{13}C NMR and MALDI-TOF spectra of LF-OMe (NMRs in DMSO).

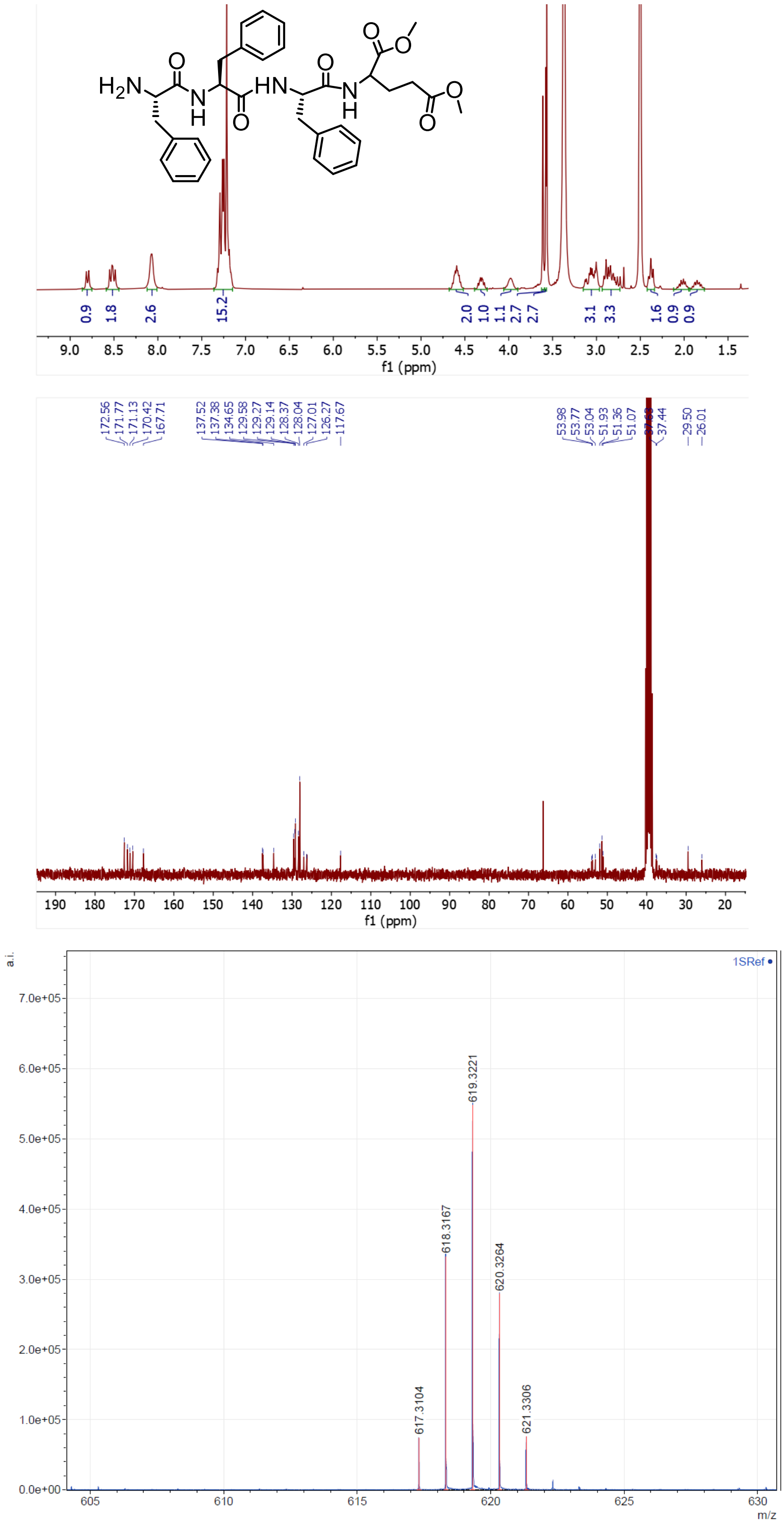

Supplementary Figure 15. ^{1}H NMR, ^{13}C NMR, and MALDI-TOF spectra of FFFE-OMe (NMRs in DMSO).

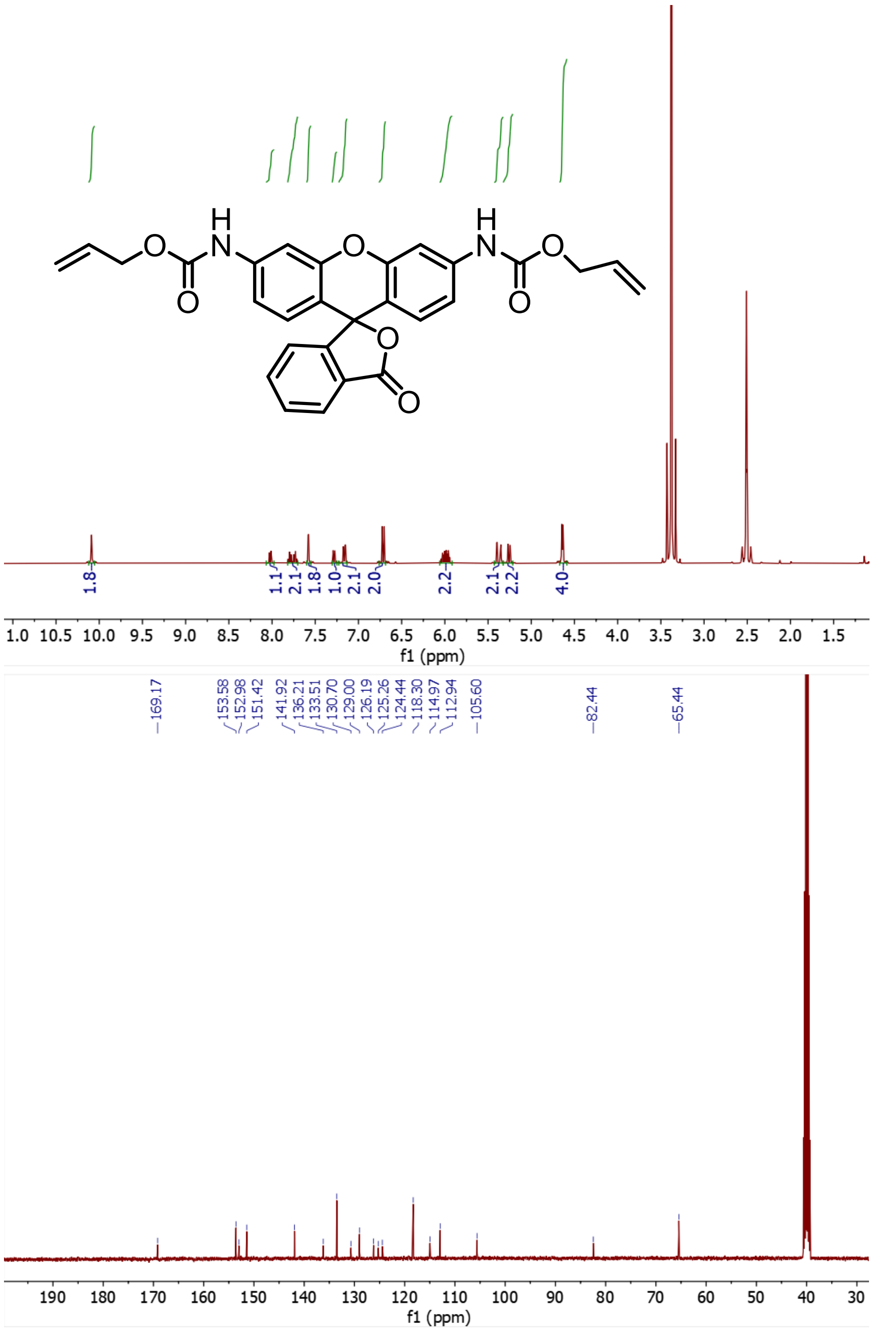

Supplementary Figure 16. ^{1}H NMR and ^{13}C NMR spectra of caged Rho-110 in DMSO.

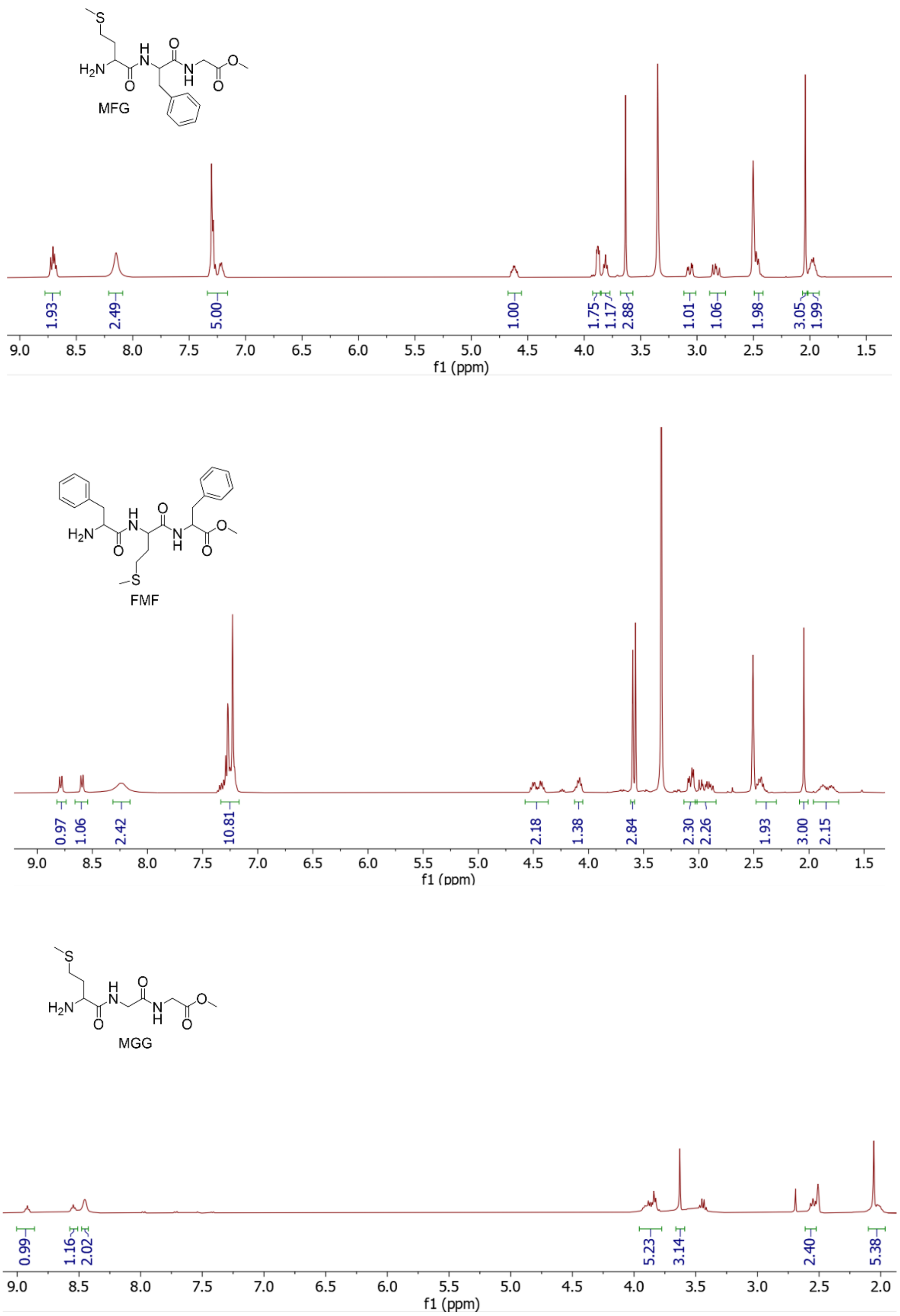

Supplementary Figure 17. ^{1}H NMR spectra of MFG, FMF and MGG in DMSO.

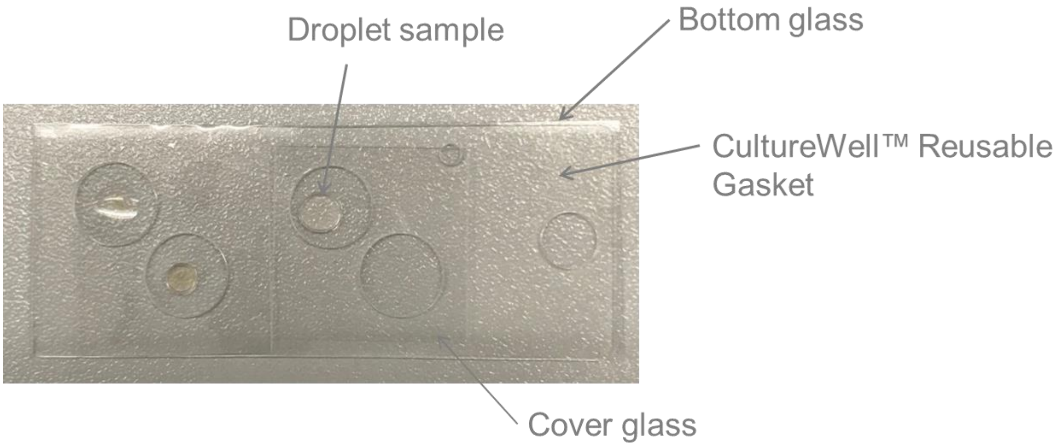

Supplementary Figure 18. Glass setup for droplet observation with microscopy imaging. In detail, a Grace Bio-Labs CultureWell™ chambered coverglass (GBL103340, Sigma) was placed on the top of a coverglass (24*60 mm, Deckglass). Droplet samples (10~20 μL) were placed in the chamber and a coverglass was placed on the top to prevent droplet evaporation.

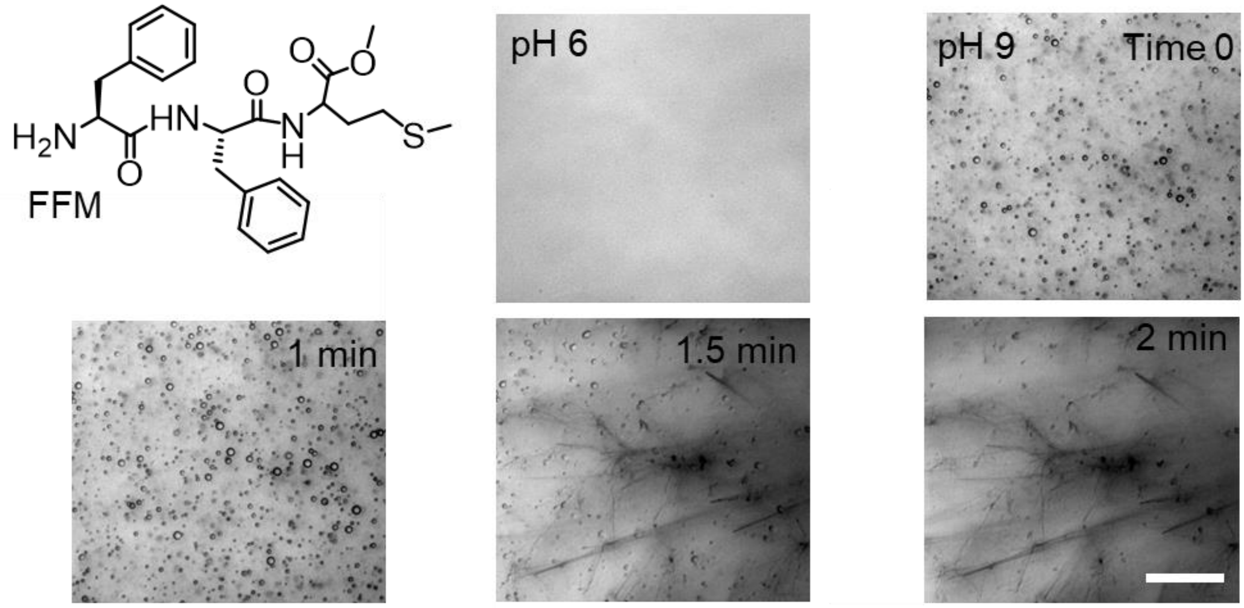

Supplementary Figure 19. Microscopic imaging (bright field) of the phase separation behavior of FFM (5 mg mL^{-1} in 5 mM HEPES with 100 mM NaCl), in which the FFM peptide is soluble in an acidic solution (i.e., pH 6). While an increase in pH resulted in phase separation and the formation of condensates, which very rapidly transformed into a fibrous material, scale bar=50 μm in all images. Similar results were obtained with 3 samples measured independently.

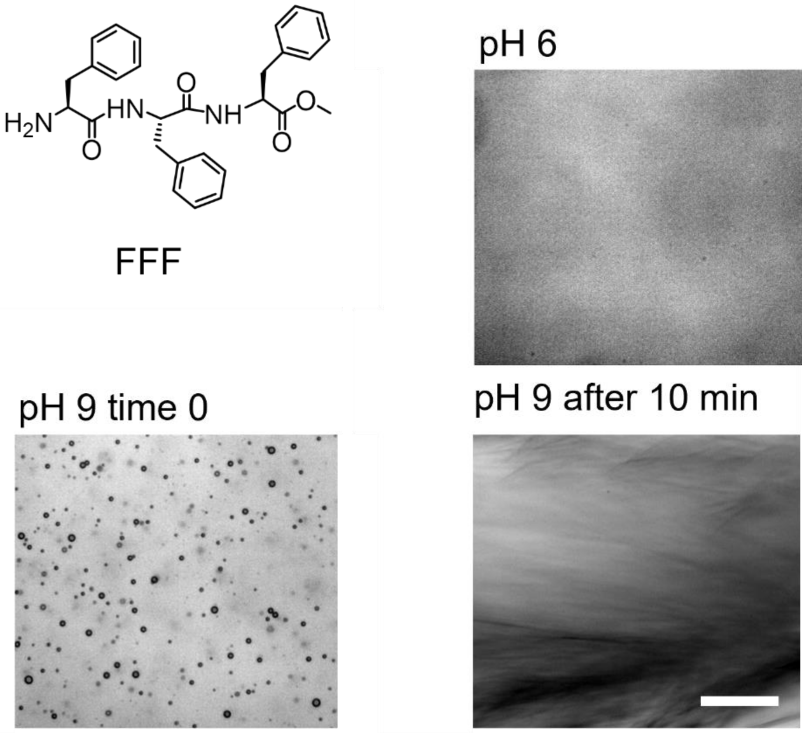

Supplementary Figure 20. Microscopic imaging (bright field) of the phase separation behavior of FFF (5 mg mL^{-1} in 5 mM HEPES with 100 mM NaCl), in which the FFF peptide is soluble in an acidic solution (i.e., pH 6). Increasing the pH resulted in phase separation and the formation of condensates, which rapidly transformed into fibrous structures, scale bar=50 μm in all images. Similar results were obtained with 3 samples measured independently.

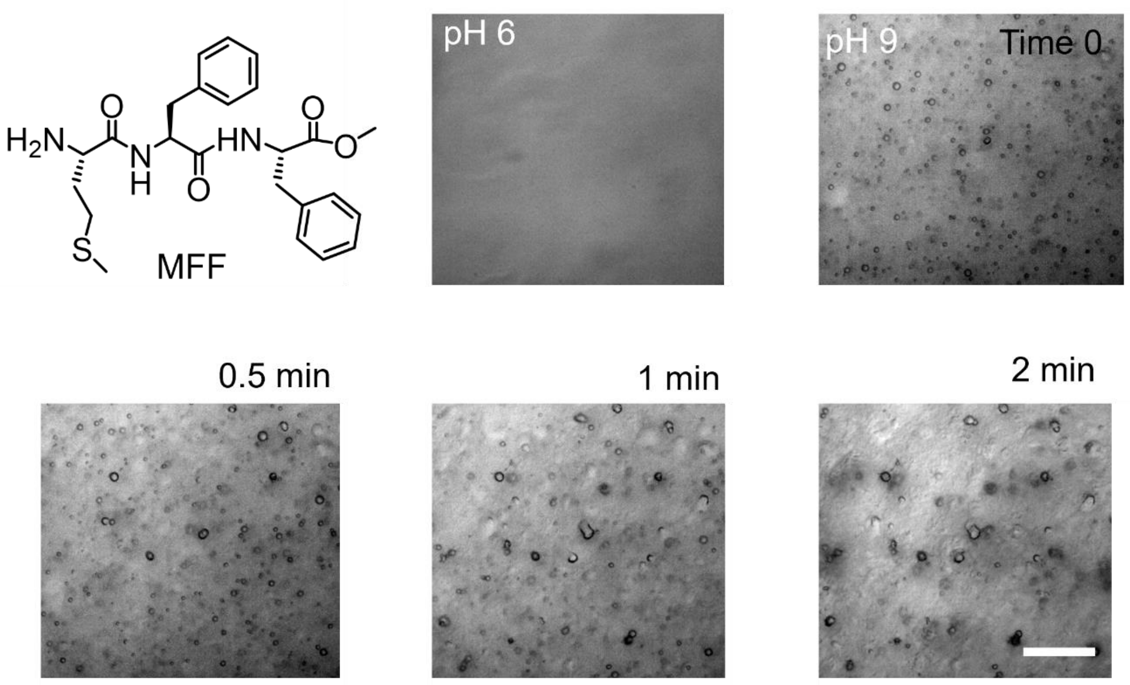

Supplementary Figure 21. Microscopic imaging (bright field) of the phase separation behavior of MFF (5 mg mL+ in 5 mM HEPES with 100 mM NaCl), in which the MFF peptide is soluble in an acidic solution (i.e., pH 6). Increasing the pH resulted in phase separation and the formation of condensates, which were rapidly transformed into aggregates, scale bar = 50 μm in all images. Similar results were obtained with 3 samples measured independently.

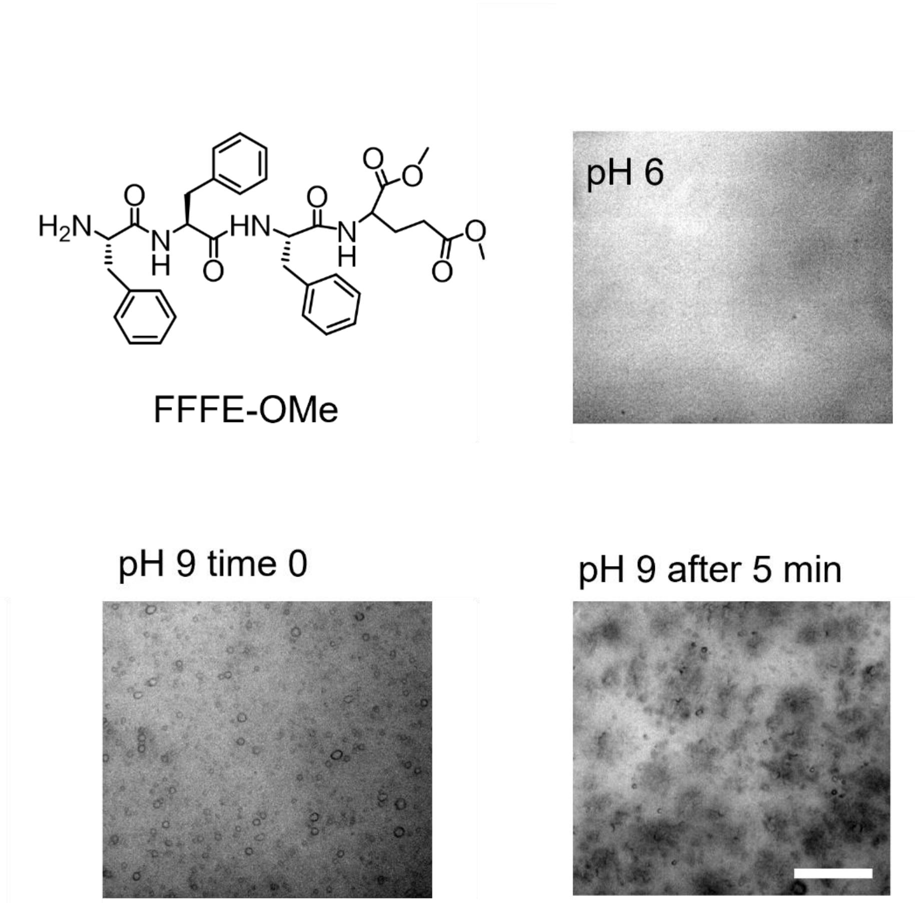

Supplementary Figure 22. Microscopic imaging (bright field) of the phase separation behavior of FFFE-OMe (5 mg mL^{-1} in 5 mM HEPES with 100 mM NaCl), in which the FFF peptide is soluble in an acidic solution (i.e., pH 6). Increasing the pH resulted in phase separation and the formation of condensates, which were rapidly transformed into aggregates, scale bar = 50 μm in all images. Similar results were obtained with 3 samples measured independently.

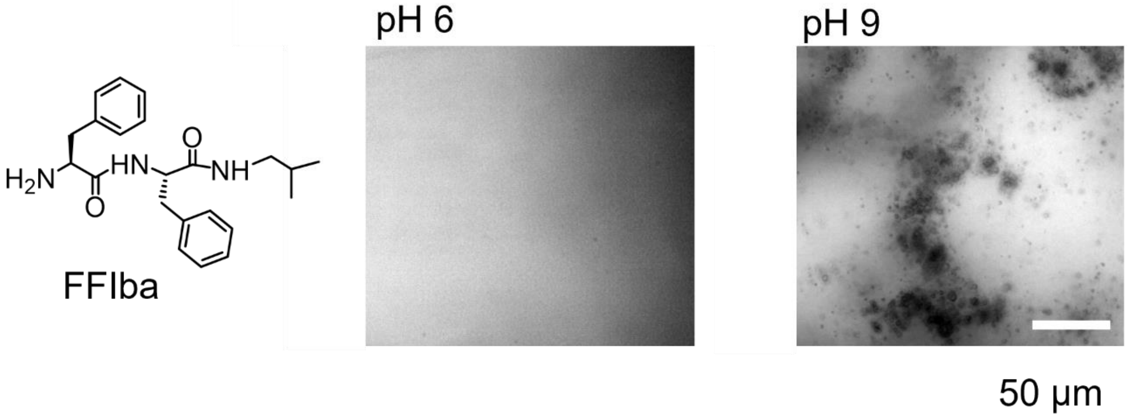

Supplementary Figure 23. Microscopic imaging (bright field) of the phase separation behavior of FFIba (5 mg mL^{-1} in 5 mM HEPES with 100 mM NaCl), in which the FFF peptide is soluble in an acidic solution (i.e., pH 6). While an increase in pH resulted in phase separation producing aggregates, scale bar=50 μm in all images. Similar results were obtained with 3 samples measured independently.

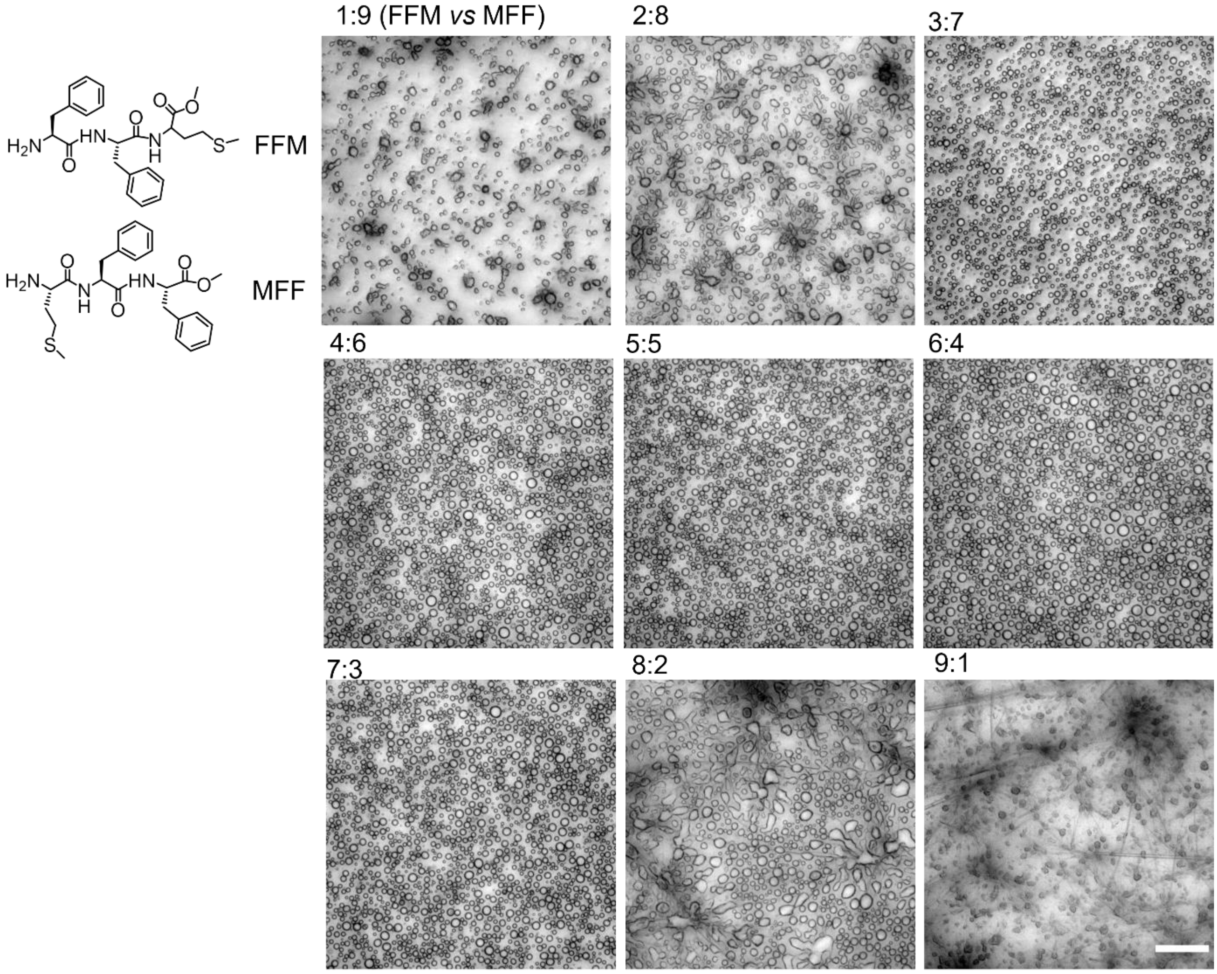

Supplementary Figure 24. The phase separation of the FFM/MFF mixture is closely related to their mixing ratios. Microscopic imaging shows that after 20 min of observation, relatively stable droplets with a weight ratio of FFM to MFF between 3:7 and 7:3 were observed, scale bar=50 μm in all images.

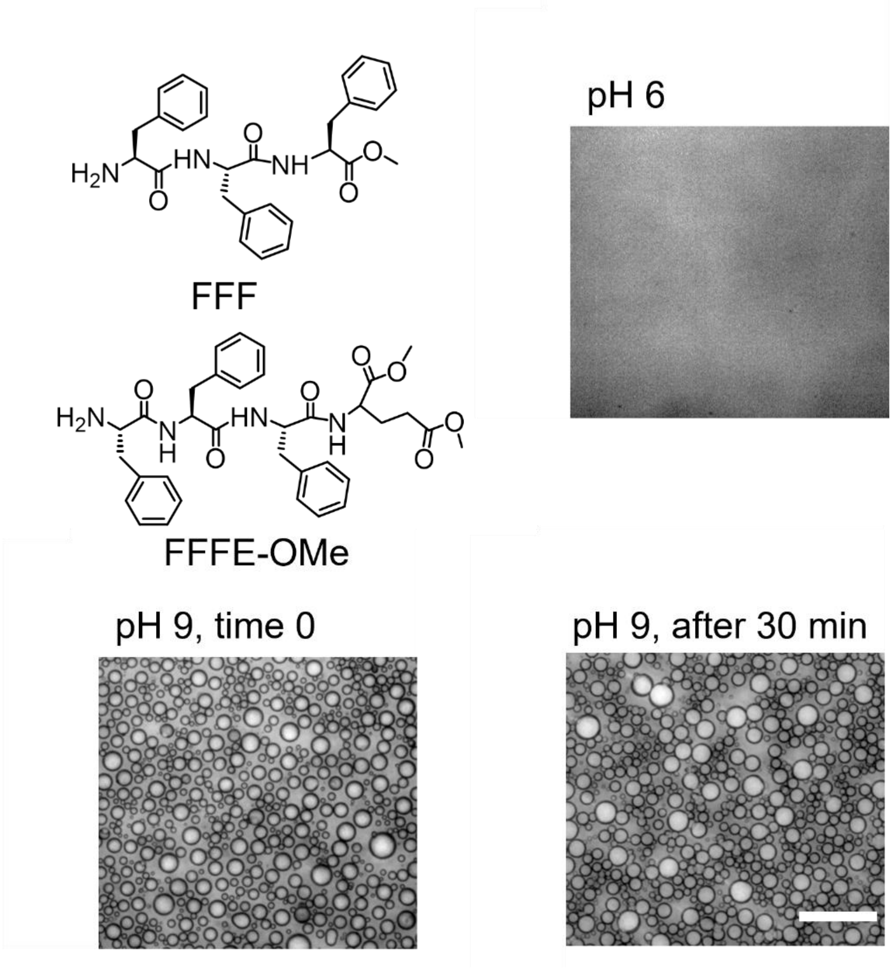

Supplementary Figure 25. Microscopic imaging (bright field) of the phase separation behavior of a peptide mixture (FFF/FFFE-OMe, 1:1 weight ratio, 5 mg mL^{-1} in 5 mM HEPES with 100 mM NaCl). The peptide mixture is soluble in an acidic solution (pH 6). Increasing the pH resulted in phase separation and the formation of droplets. No solid/fiber transition was observed over 30 min, scale bar = 50 μm in all images.

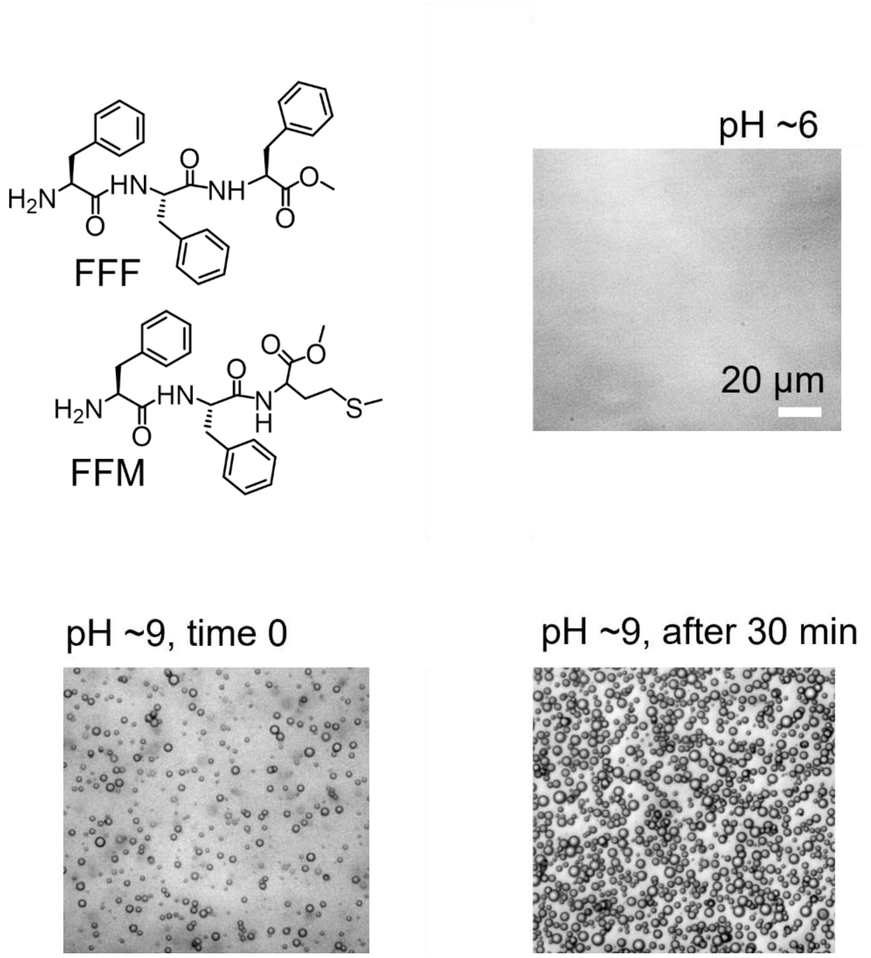

Supplementary Figure 26. Microscopic imaging (bright field) of the phase separation behavior of a peptide mixture (FFF/FFM, 1:1 weight ratio, 5 mg mL^{-1} in 5 mM HEPES with 100 mM NaCl). The peptide mixture is soluble in an acidic solution (pH 6). Increasing the pH resulted in phase separation and the formation of droplets. No solid/fiber transition was observed over 30 min, scale bar = 20 µm in all images.

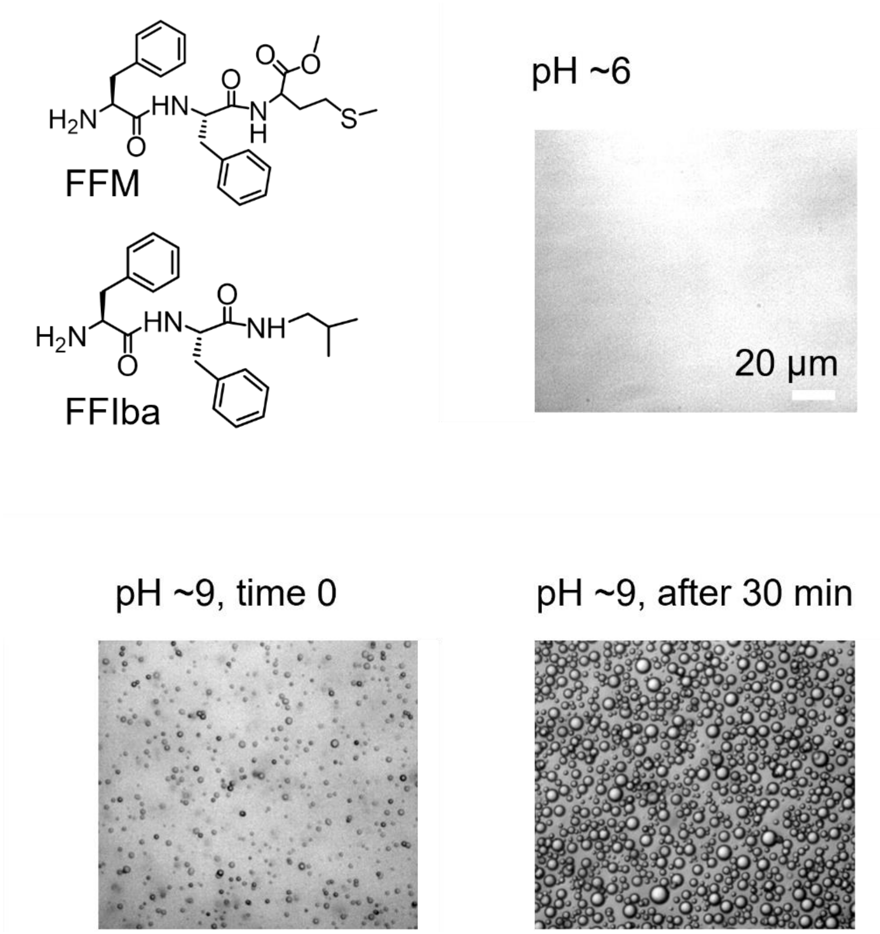

Supplementary Figure 27. Microscopic imaging (bright field) of the phase separation behavior of a peptide mixture (FFM/FFIba, 1:1 weight ratio, 5 mg mL^{-1} in 5 mM HEPES with 100 mM NaCl). The peptide mixture is soluble in an acidic solution (pH 6). Increasing the pH resulted in phase separation and the formation of droplets. No solid/fiber transition was observed over 30 min, scale bar = 20 μm in all images.

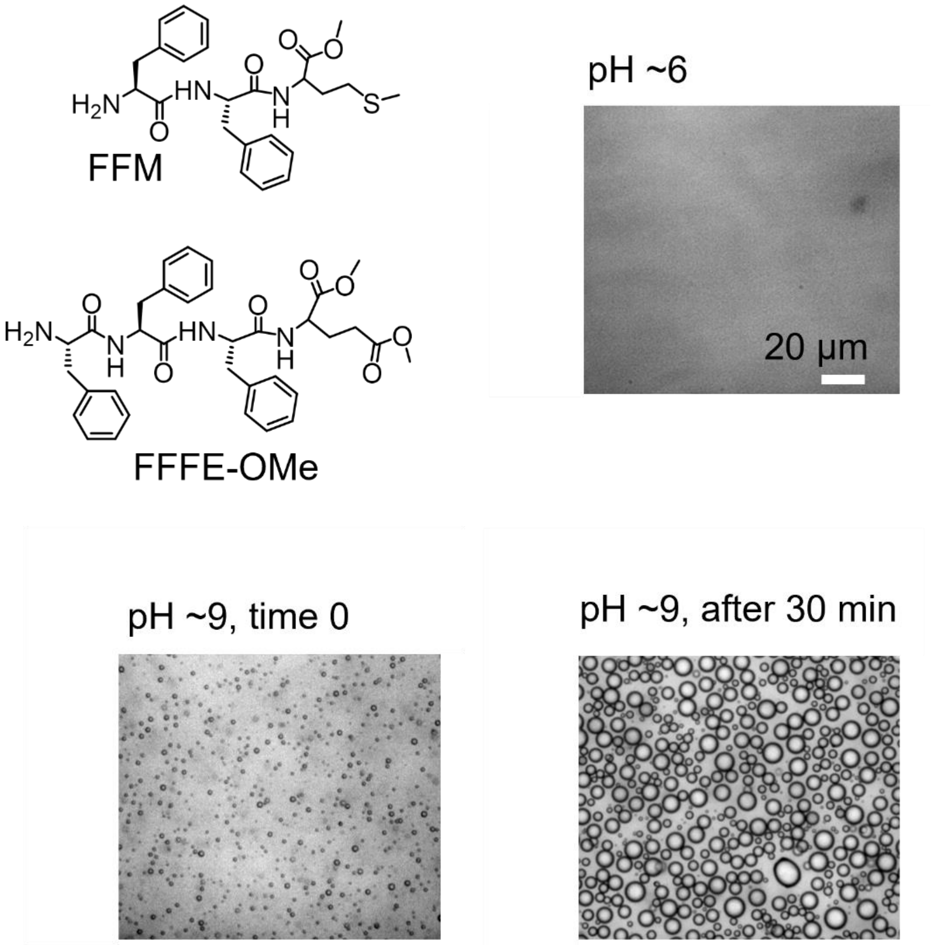

Supplementary Figure 28. Microscopic imaging (bright field) of the phase separation behavior of a peptide mixture (FFM/FFFE-OMe, 1:1 weight ratio, 5 mg mL^{-1} in 5 mM HEPES with 100 mM NaCl). The peptide mixture is soluble in an acidic solution (pH 6). Increasing the pH resulted in phase separation and the formation of droplets. No solid/fiber transition was observed over 30 min, scale bar = 20 μm in all images in all images.

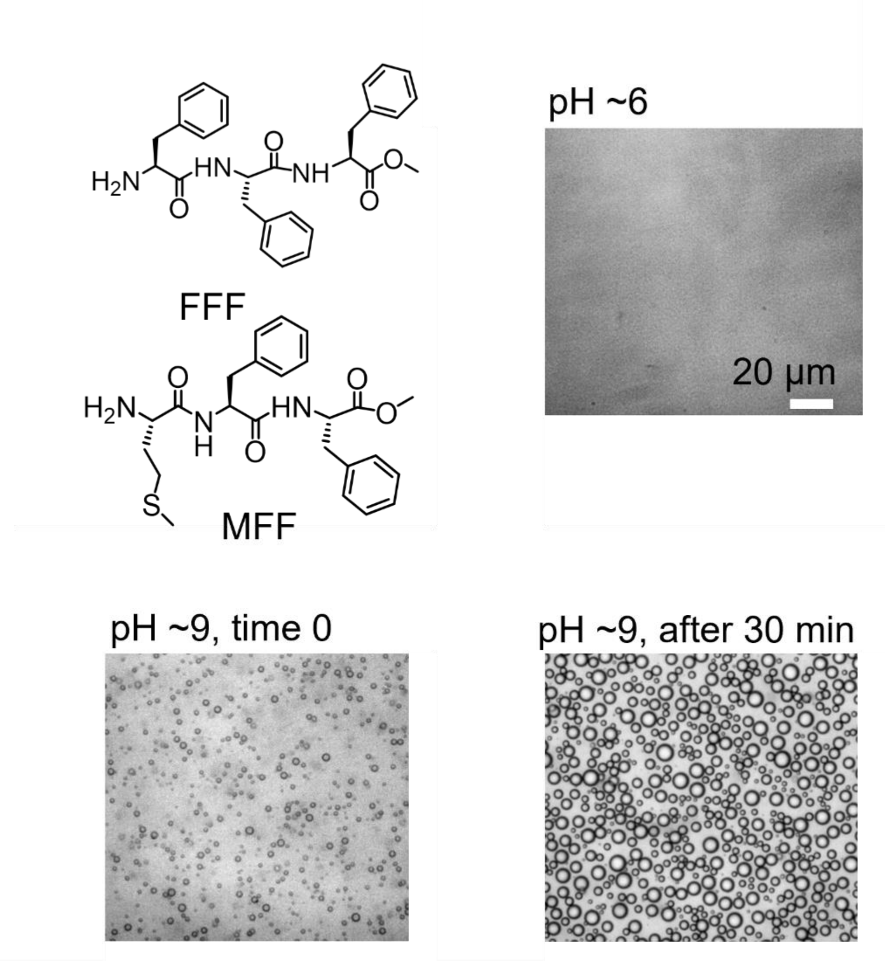

Supplementary Figure 29. Microscopic imaging (bright field) of the phase separation behavior of a peptide mixture (FFF/MFF, 1:1 weight ratio, 5 mg mL^{-1} in 5 mM HEPES with 100 mM NaCl). The peptide mixture is soluble in an acidic solution (pH 6). Increasing the pH resulted in phase separation and the formation of droplets. No solid/fiber transition was observed over 30 min, scale bar = 20 μm in all images.

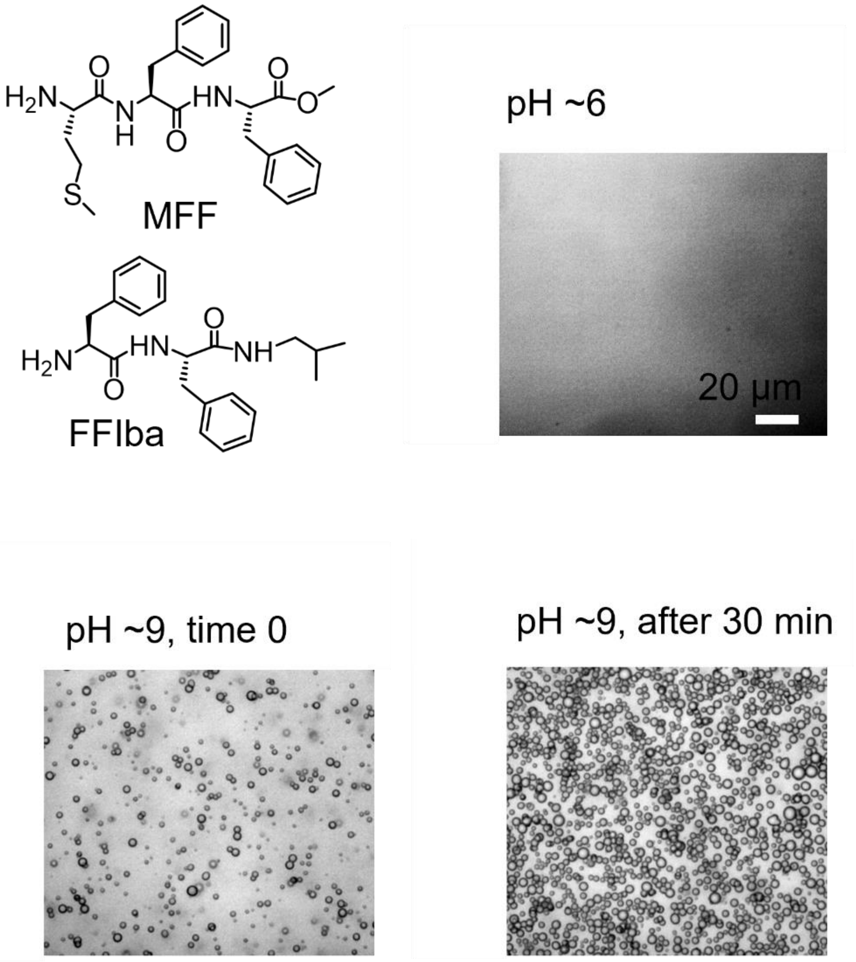

Supplementary Figure 30. Microscopic imaging (bright field) of the phase separation behavior of a peptide mixture (MFF/FFIba, 1:1 weight ratio, 5 mg mL^{-1} in 5 mM HEPES with 100 mM NaCl). The peptide mixture is soluble in an acidic solution (pH 6). Increasing the pH resulted in phase separation and the formation of droplets. No solid/fiber transition was observed over 30 min, scale bar = 20 μm in all images.

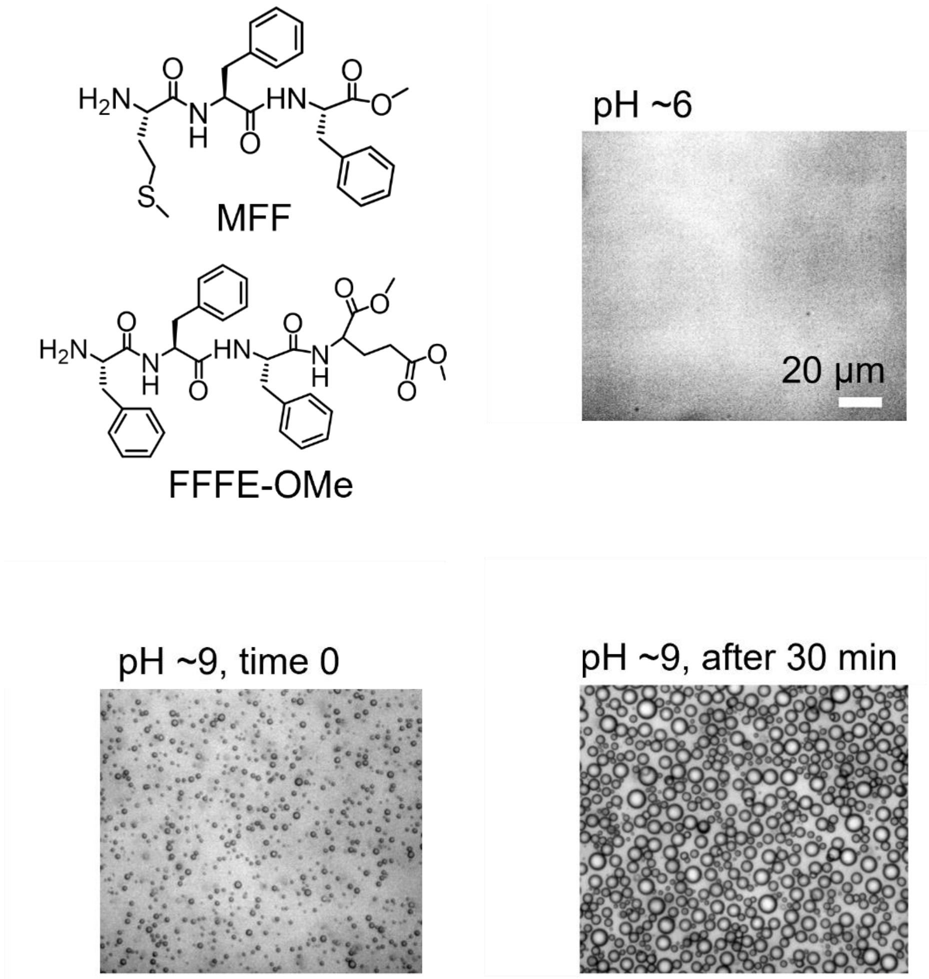

Supplementary Figure 31. Microscopic imaging (bright field) of the phase separation behavior of a peptide mixture (MFF/FFFE-OMe, 1:1 weight ratio, 5 mg mL^{-1} in 5 mM HEPES with 100 mM NaCl). The peptide mixture is soluble in an acidic solution (pH 6). Increasing the pH resulted in phase separation and the formation of droplets. No solid/fiber transition was observed over 30 min, scale bar = 20 μm in all images.

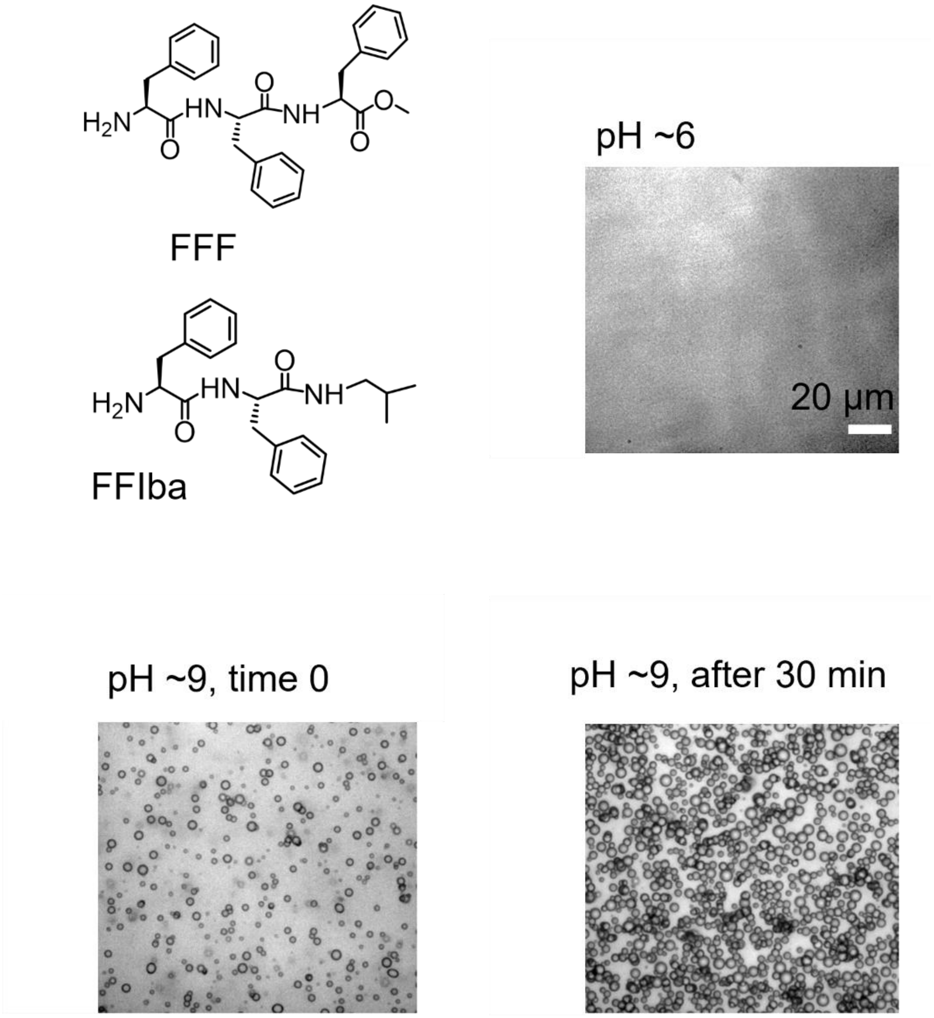

Supplementary Figure 32. Microscopic imaging (bright field) of the phase separation behavior of a peptide mixture (FFF/FFIba, 1:1 weight ratio, 5 mg mL^{-1} in 5 mM HEPES with 100 mM NaCl). The peptide mixture is soluble in an acidic solution (pH 6). Increasing the pH resulted in phase separation and the formation of droplets. No solid/fiber transition was observed over 30 min, scale bar = 20 μm in all images.

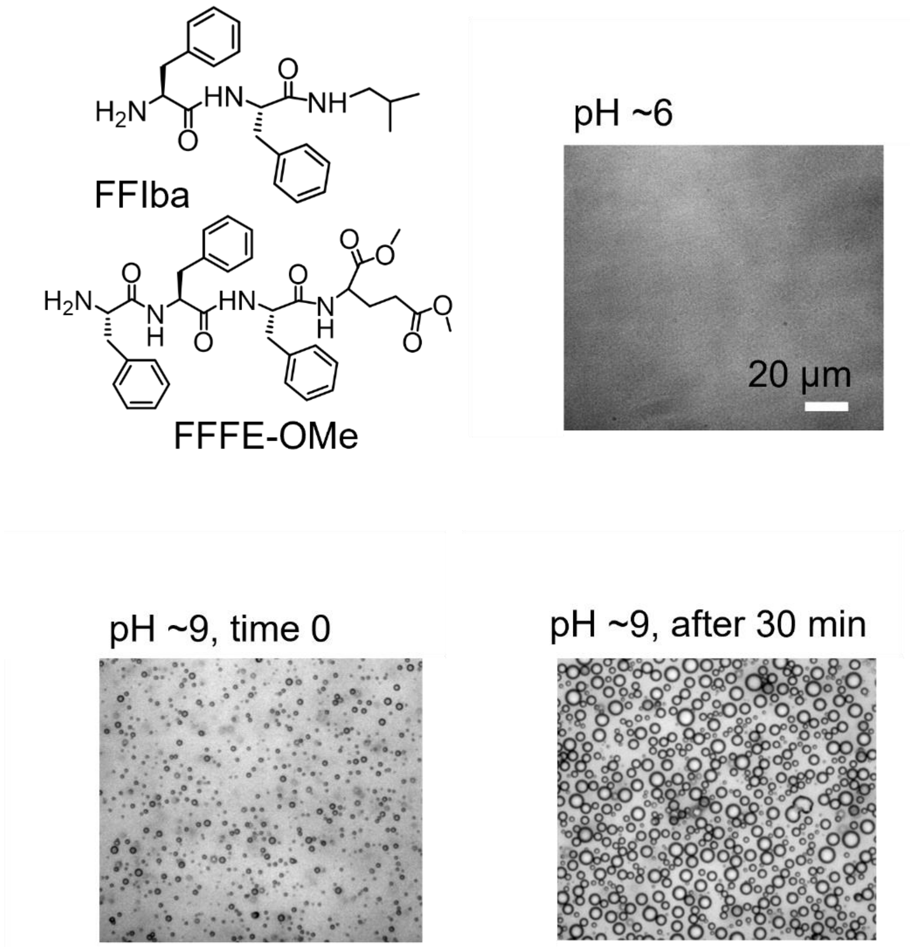

Supplementary Figure 33. Microscopic imaging (bright field) of the phase separation behavior of a peptide mixture (FFIba/FFFE-OMe, 1:1 weight ratio, in total 5 mg mL^{-1} in 5 mM HEPES with 100 mM NaCl). The peptide mixture is soluble in an acidic solution (pH 6). Increasing the pH resulted in phase separation and the formation of droplets. No solid/fiber transition was observed over 30 min, scale bar = 20 μm in all images.

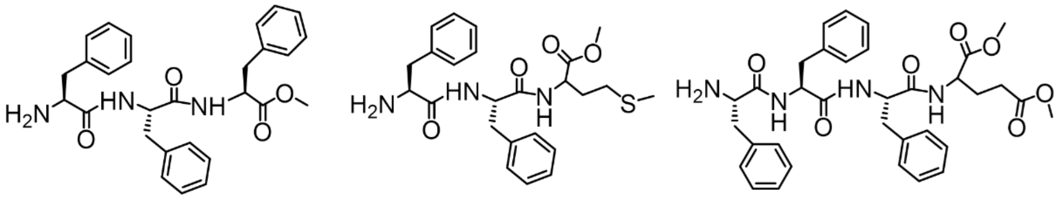

FFF FFM FFFE-OMe

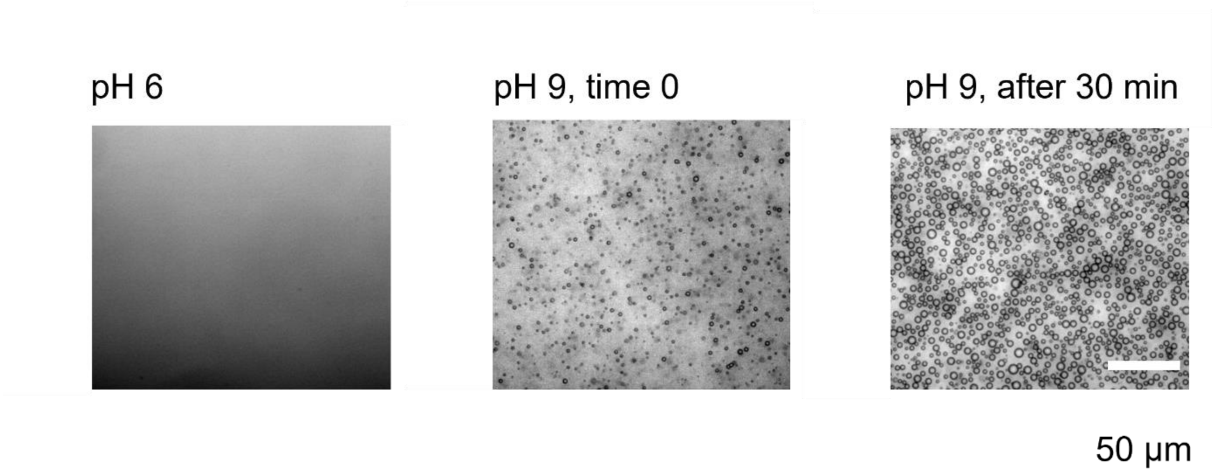

Supplementary Figure 34. Microscopic imaging (bright field) of the phase separation behavior of peptides mixture (FFF/FFM/FFFE-OMe, 1:1:1 weight ratio, in total 3.75 mg mL^{-1} in 5 mM HEPES with 100 mM NaCl). The peptide mixture is soluble in an acidic solution (pH 6). Increasing the pH resulted in phase separation and the formation of droplets. No solid/fiber transition was observed over 30 min, scale bar = 50 µm in all images.

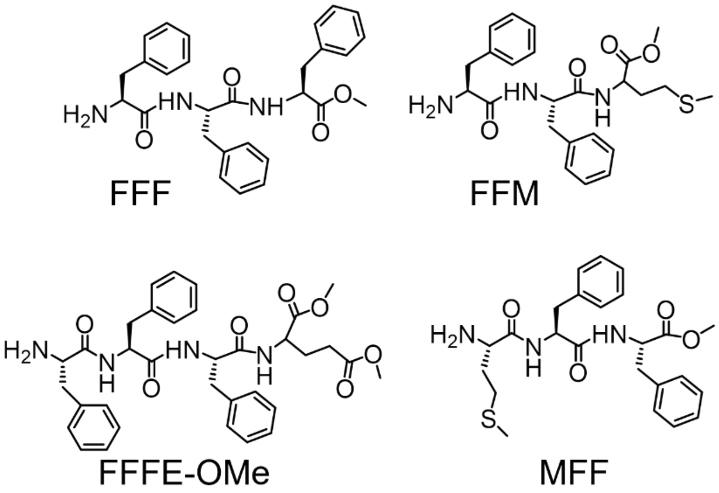

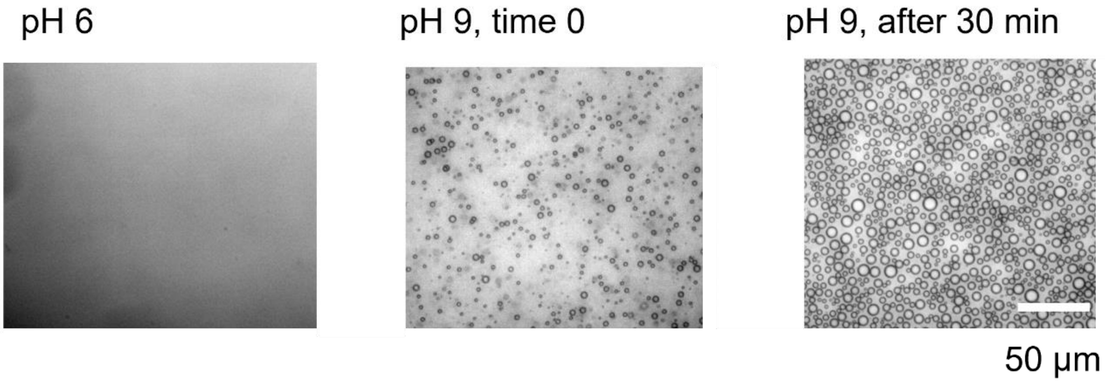

Supplementary Figure 35. Microscopic imaging (bright field) of the phase separation behavior of a peptide mixture (FFF/FFM/FFFE-OMe/MFF, 1:1:1:1 weight ratio, in total 5 mg mL^{-1} in 5 mM HEPES with 100 mM NaCl). The peptide mixture is soluble in an acidic solution (pH 6). Increasing the pH resulted in phase separation and the formation of droplets. No solid/fiber transition was observed over 30 min, scale bar = 50 μm in all images.

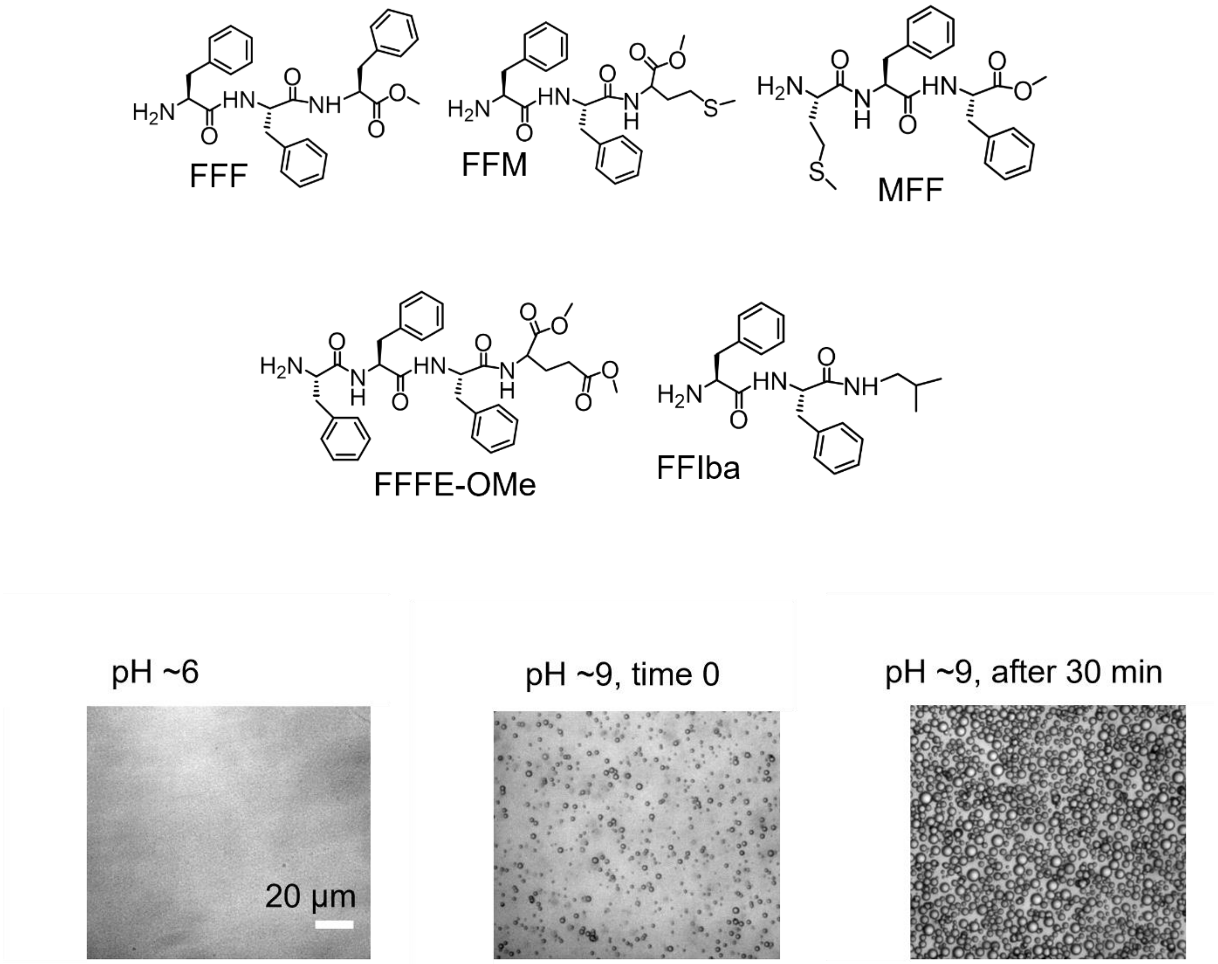

Supplementary Figure 36. Microscopic imaging (bright field) of the phase separation behavior of a peptide mixture (FFF/FFM/FFFE-OMe/MFF/FFIba, 1:1:1:1:1 weight ratio, in total 5 mg mL^{-1} in 5 mM HEPES with 100 mM NaCl). The peptide mixture is soluble in an acidic solution (pH 6). Increasing the pH resulted in phase separation and the formation of droplets. No solid/fiber transition was observed over 30 min, scale bar = 50 μm in all images.

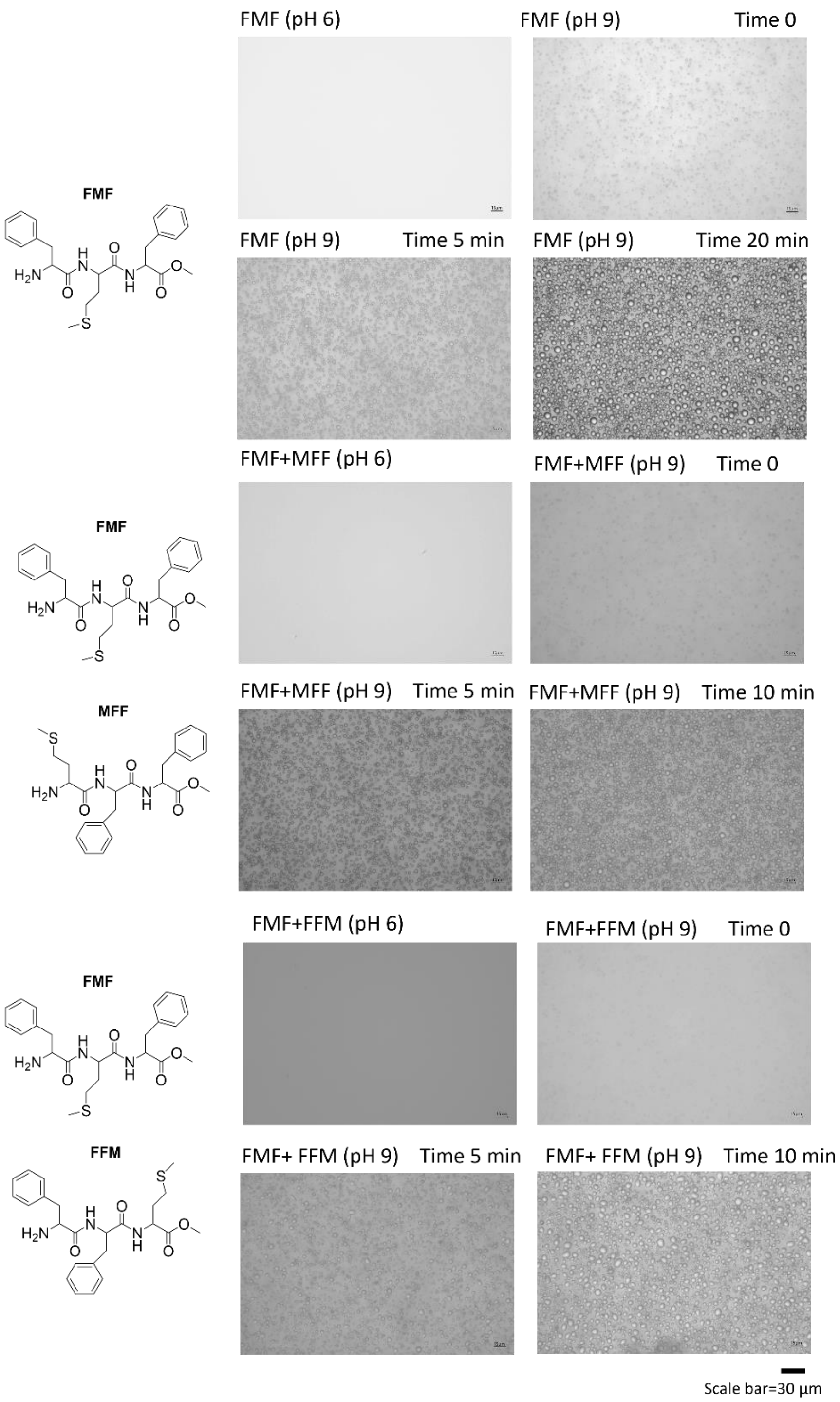

Supplementary Figure 37. Top: Microscopic imaging (bright field) of FMF at pH 6 and pH 9 (5 mg mL^{-1}). Middle: Microscopic imaging showing the phase behavior of a 2-peptide solution (MFM+MFF, 1:1, in total 5 mg mL^{-1} in 5 mM HEPES with 100 mM NaCl). The solution is clear at pH 6. Increasing the pH to 9 resulted in phase separation, producing relatively stable droplets. Scale bar=30 μm in all images. Bottom: Microscopic imaging showing the phase behavior of a 2-peptide solution (MFM+FFM, 1:1, in total 5 mg mL^{-1} in 5 mM HEPES with 100 mM NaCl). The solution is clear at pH 6. Increasing the pH to 9 resulted in phase separation, producing relatively stable droplets. Scale bar=30 μm in all images.

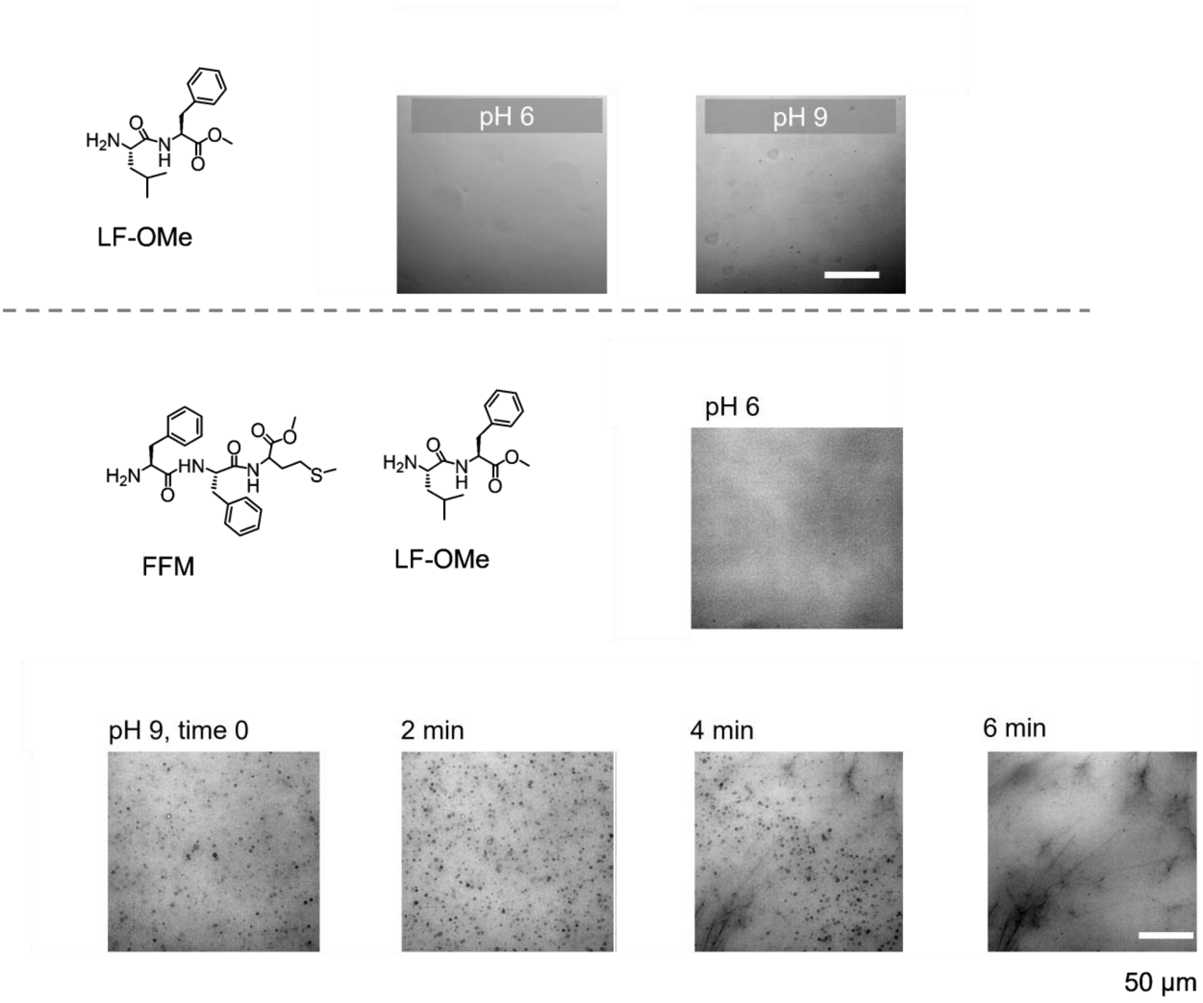

Supplementary Figure 38. Top: Microscopic imaging (bright field) of LF-OMe at pH 6 and pH 9 (5 mg mL^{-1}). Bottom: Microscopic imaging showing the phase behavior of a 2-peptide solution (FFM/LF-OMe, 1:1, in total 5 mg mL^{-1} in 5 mM HEPES with 100 mM NaCl). The solution is clear at pH 6. Increasing the pH to 9 resulted in phase separation, producing droplets that rapidly transformed into fibers. Scale bar=50 μm in all images.

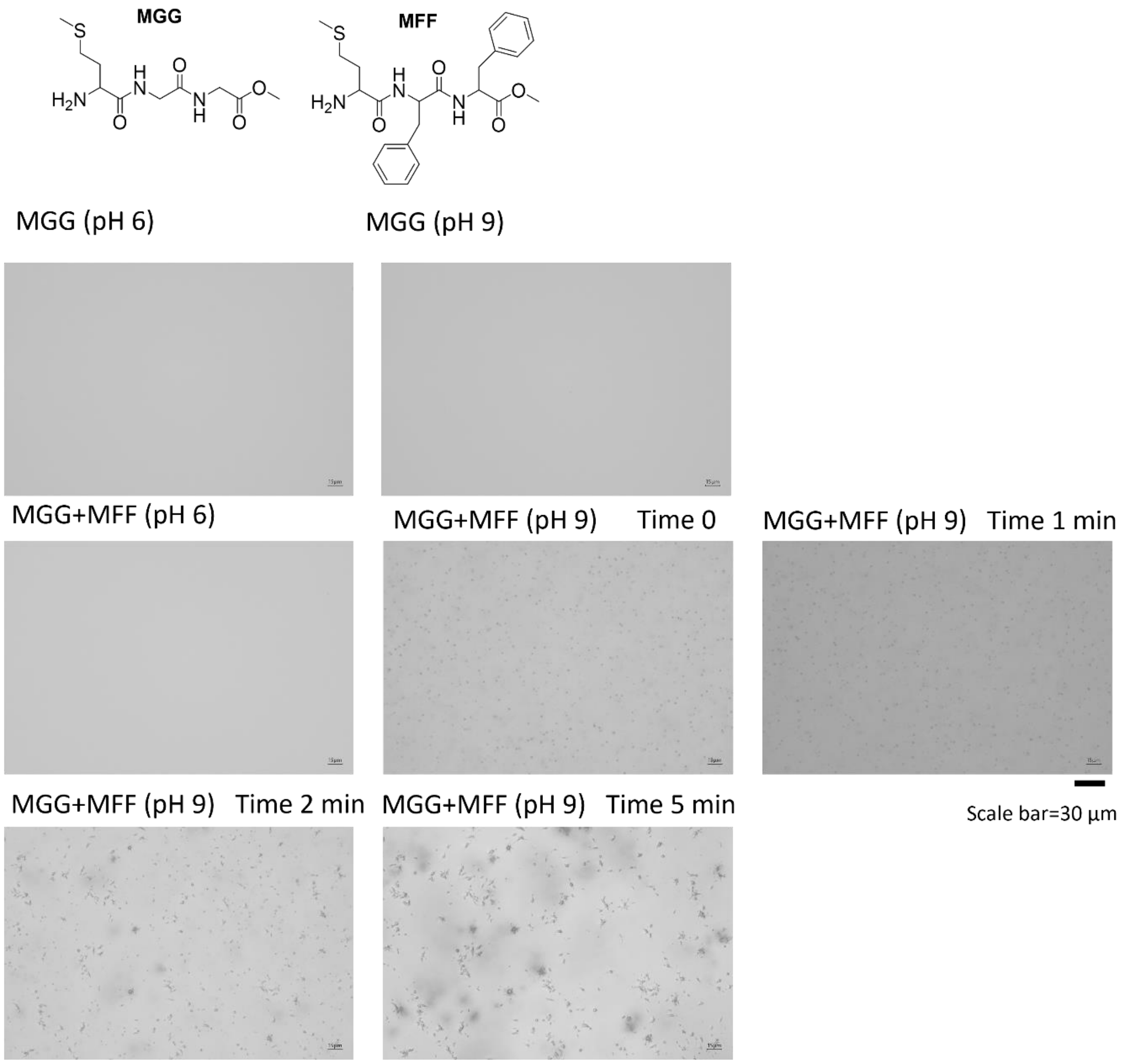

Supplementary Figure 39. Top: Microscopic imaging (bright field) of MGG at pH 6 and pH 9 (5 mg mL^{-1}). Bottom: Microscopic imaging showing the phase behavior of a 2-peptide solution (MGG+MFF, 1:1 weight ratio, in total 5 mg mL^{-1} in 5 mM HEPES with 100 mM NaCl). The solution is clear at pH 6. Increasing the pH to 9 resulted in phase separation and the formation of droplets, which rapidly transformed into irregular aggregates. Scale bar=30 µm in all images.

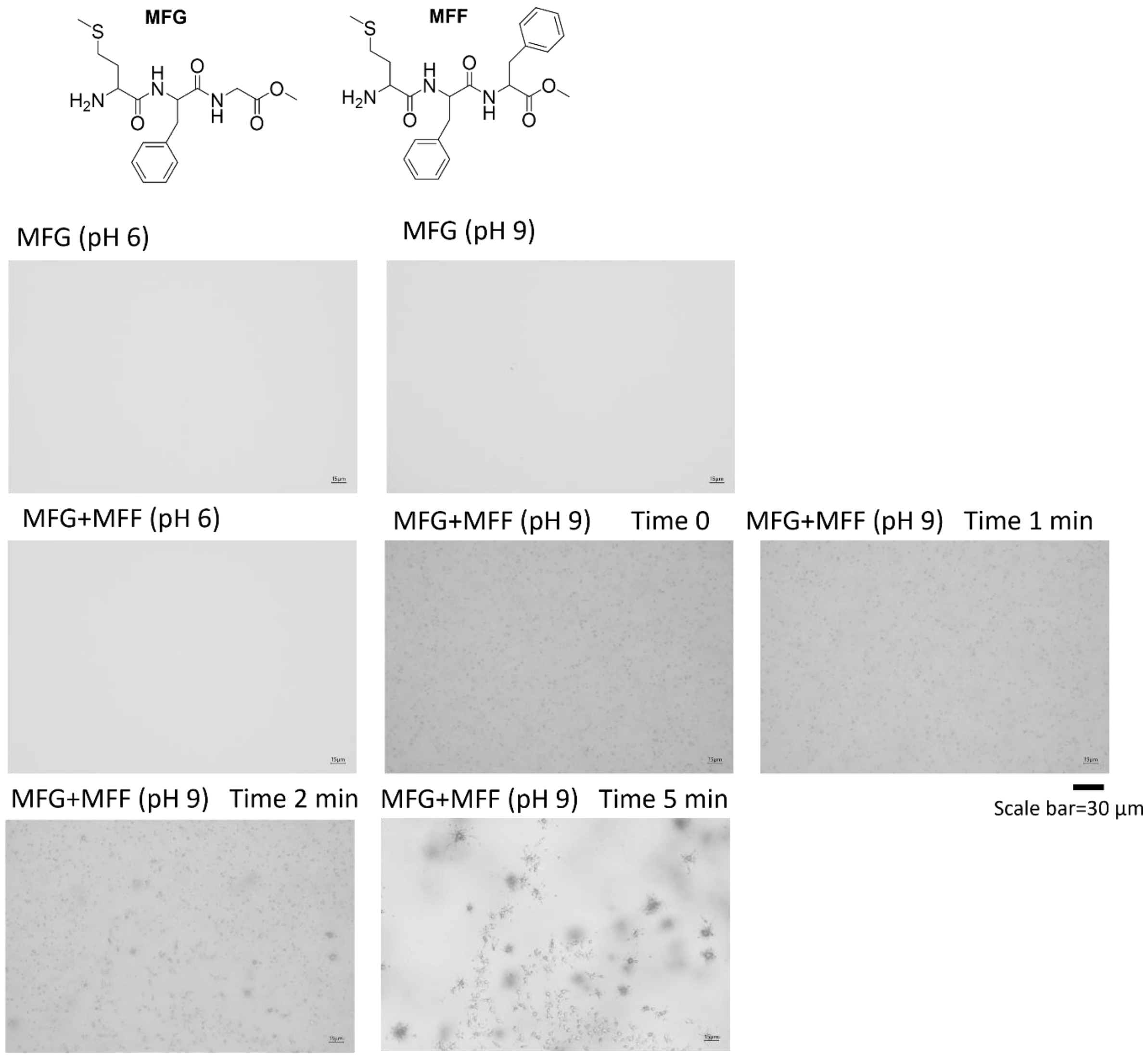

Supplementary Figure 40. Top: Microscopic imaging (bright field) of MFG at pH 6 and pH 9 (5 mg mL^{-1}). Bottom: Microscopic imaging showing the phase behavior of a 2-peptide solution (MFG+MFF, 1:1 weight ratio, in total 5 mg mL^{-1} in 5 mM HEPES with 100 mM NaCl). The solution is clear at pH 6. Increasing the pH to 9 resulted in phase separation and the formation of droplets, which rapidly transformed into irregular aggregates. Scale bar=30 µm in all images.

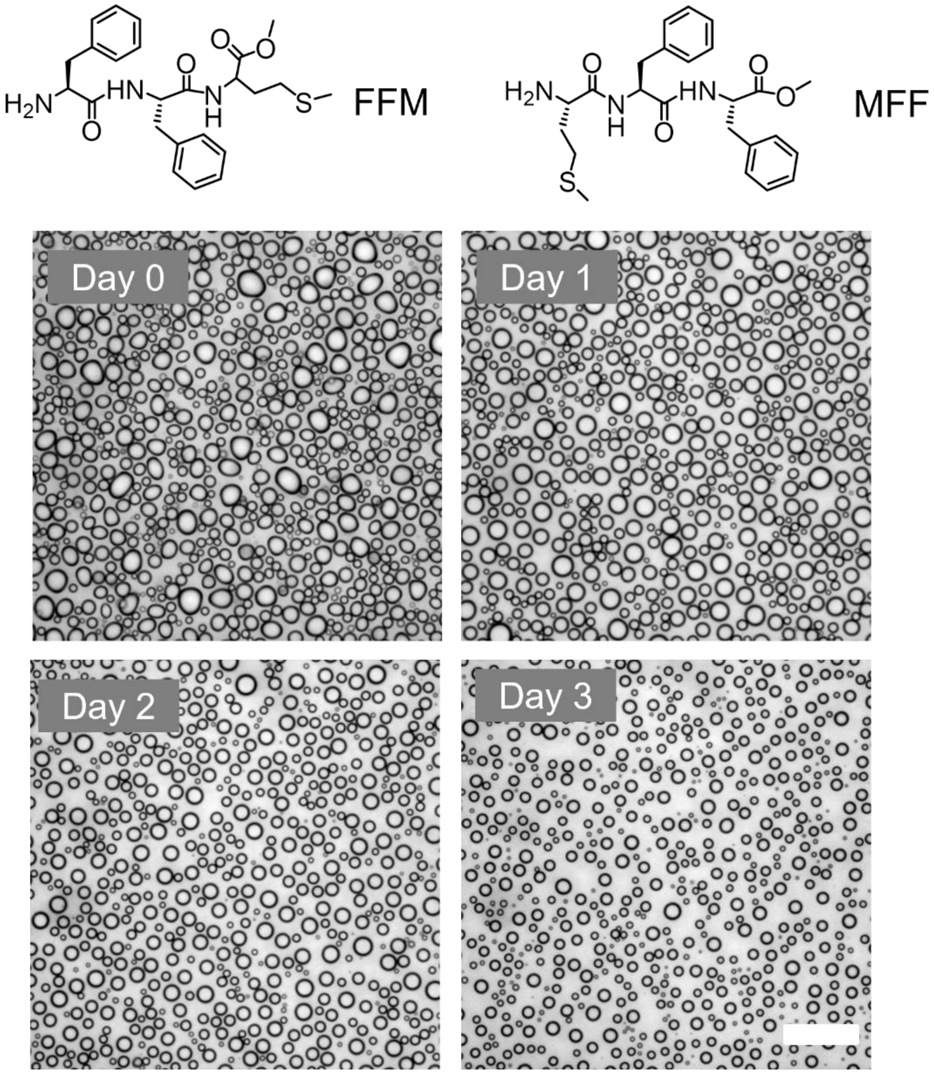

Supplementary Figure 41. Stability of FFM/MFF coacervates (1:1 weight ratio, total 5 mg mL^{-1}) by incubation over 3 days, scale bar=20 μm in all images. Similar results were obtained with 3 samples measured independently.

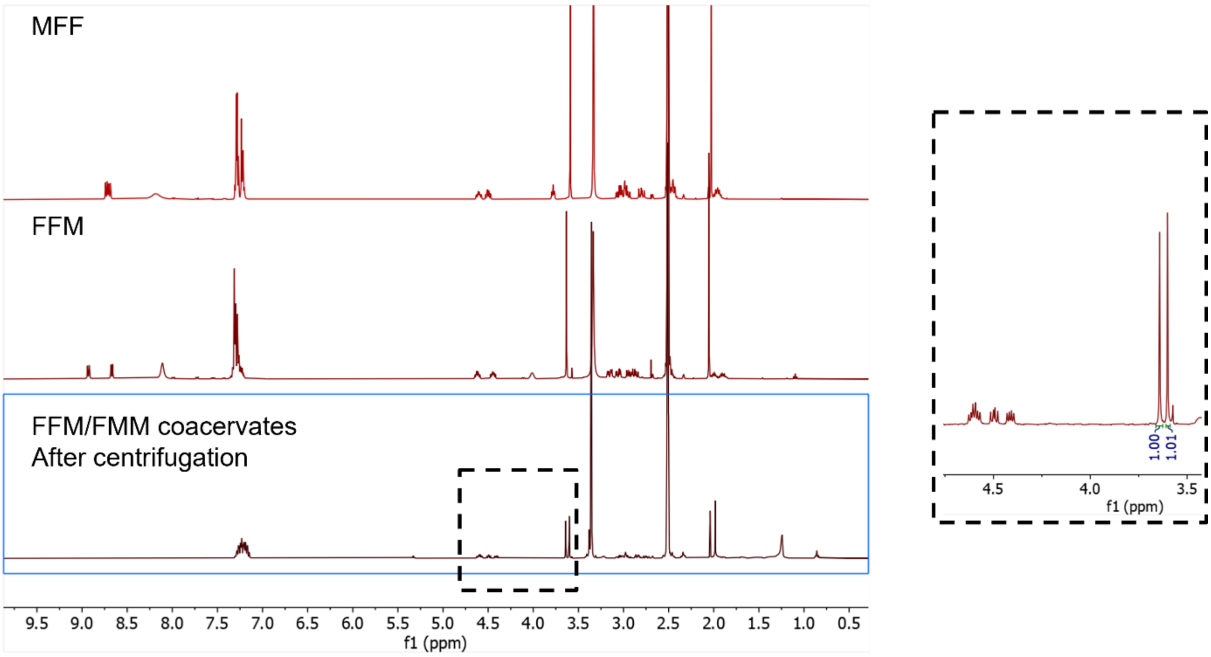

Supplementary Figure 42. ^{1}H NMR analysis of individual solutions of MFF and FFM peptides in DMSO and the composition of the binary peptide coacervate phase FFM/MFF obtained after centrifugation and redissolution in DMSO. Integration of the -OCH_3 proton peaks for the FFM and MFF peptides indicated that the ratio of FFM to MFF in the binary peptide coacervates was close to the 1:1 feed ratio.

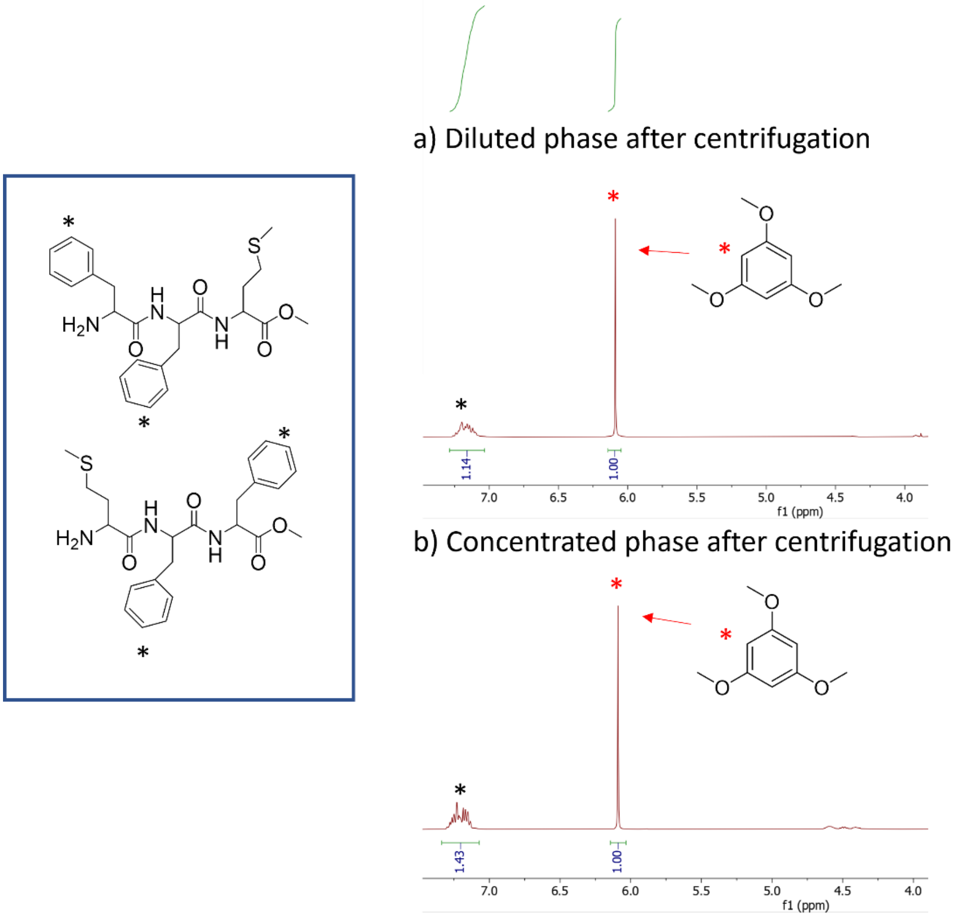

Supplementary Figure 43. ^{1}H NMR analysis of relative peptide content in the dilute phase and concentrated phase for MFF/FFM. Phase separation was achieved by low-speed centrifugation and redissolution in DMSO. Each phase was analyzed using 1,3,5-trimethoxybenzene as an external standard. Integration of the aromatic proton peaks (both peptides) indicated that the ratio of total peptide content in the concentrated phase compared to the diluted phase was approximately 1.25:1.

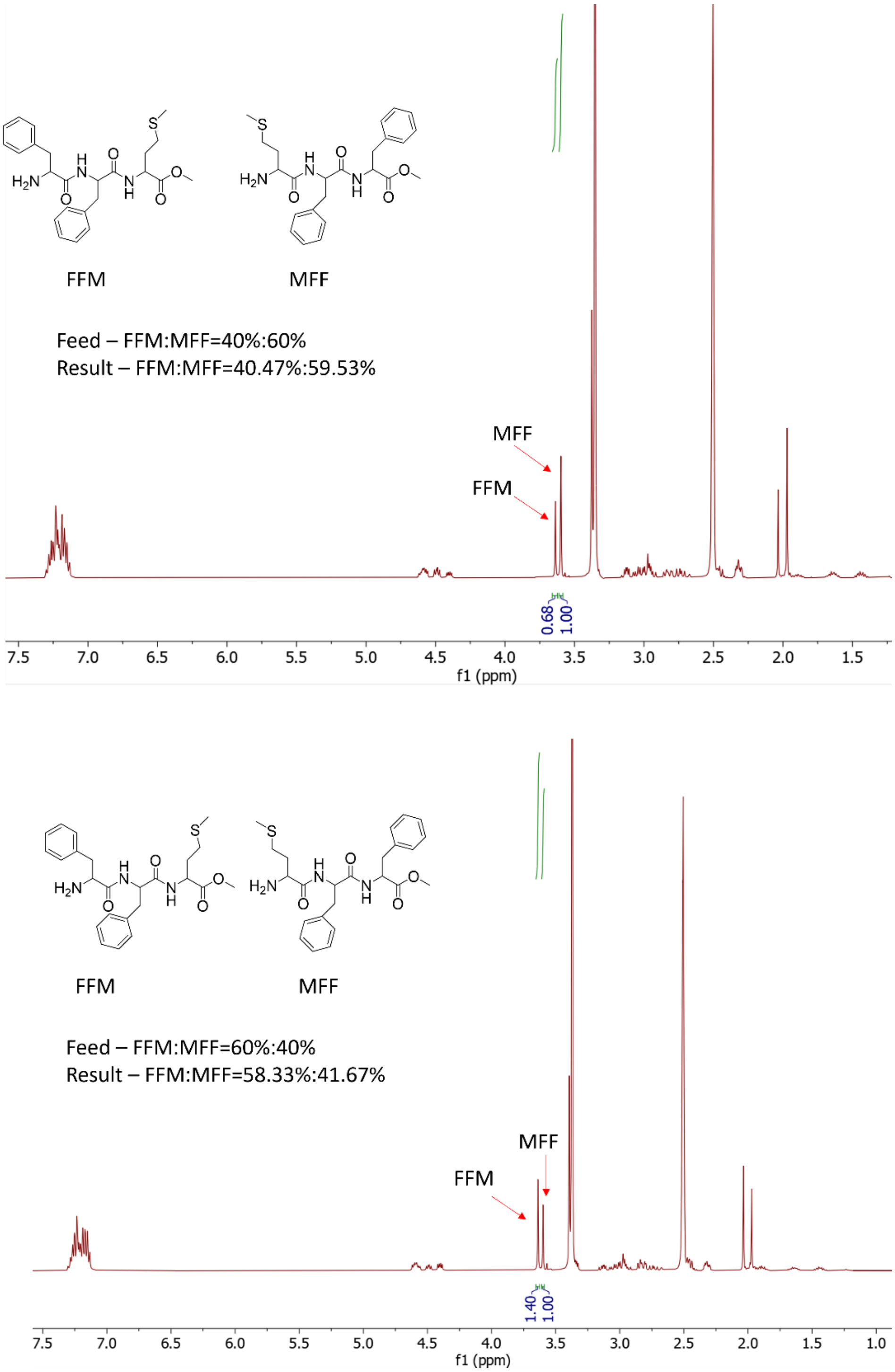

Supplementary Figure 44. ^{1}H NMR analysis of individual solutions of MFF and FFM peptides in DMSO and the composition of the binary peptide coacervate phase FFM/MFF obtained after centrifugation and redissolution in DMSO. Integration of the -OCH_3 proton peaks for the FFM and MFF peptides indicated that the ratio of FFM to MFF in the binary peptide coacervates closely corresponds to their initial feed ratios (60% FFM and 40%, MFF).

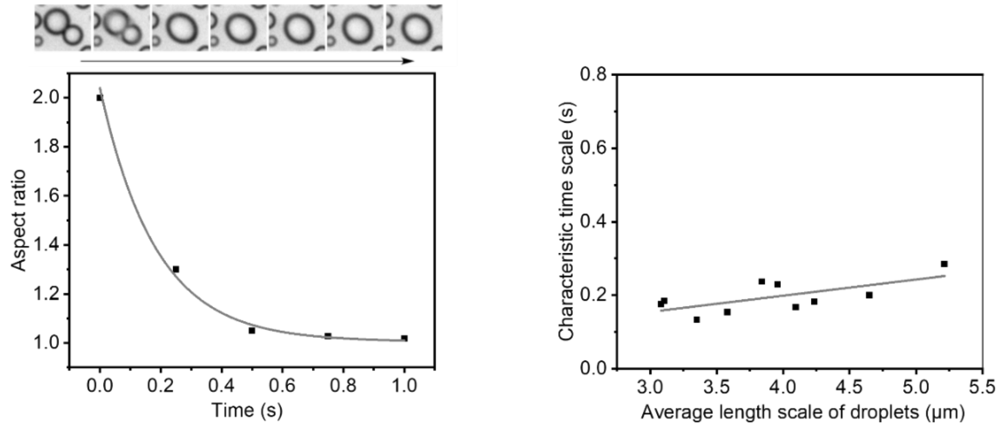

Supplementary Figure 45. Left: Representative changes in the aspect ratio of two coalescing droplets exhibit an an exponential decay. The characteristic fusion time of fusion τ was obtained from equation $y = A_1 \exp(-x/\tau) + y_0$, fitted with Origin 2021. In the representative case shown in the left the τ is calculated to be 0.183 s. Right: The characteristic time (τ) is plotted against the average length scale of the droplets ($l_{average}$). The slope of the fitted linear function represents the inverse capillary velocity, which corresponds to the viscosity-to-surface tension ratio. (η/γ) ≈ 0.044 s/μm.

$$\tau \approx l_{average} \times \left(\frac{\eta}{\gamma}\right)$$

τ: characteristic time scale. Time it takes for two droplets to fuse and assume a spherical shape.

$l_{average}$: characteristic length scale. Estimated as the average initial diameter of two merging droplets.

η: viscosity of the coacervate phase.

γ: interfacial tension between coacervate phase and dilute phase

Droplet pair	Characteristic time scale (τ)	Average droplet diameter ($l_{average}$)
1	0.285	5.21
2	0.237	3.84
3	0.229	3.96
4	0.133	3.35
5	0.185	3.10
6	0.154	3.58
7	0.200	4.65
8	0.183	4.23
9	0.176	3.08
10	0.167	4.09

Supplementary Table 1. Average droplet diameter ($l_{average}$) (um) and characteristic time scale (τ) of representative droplets.

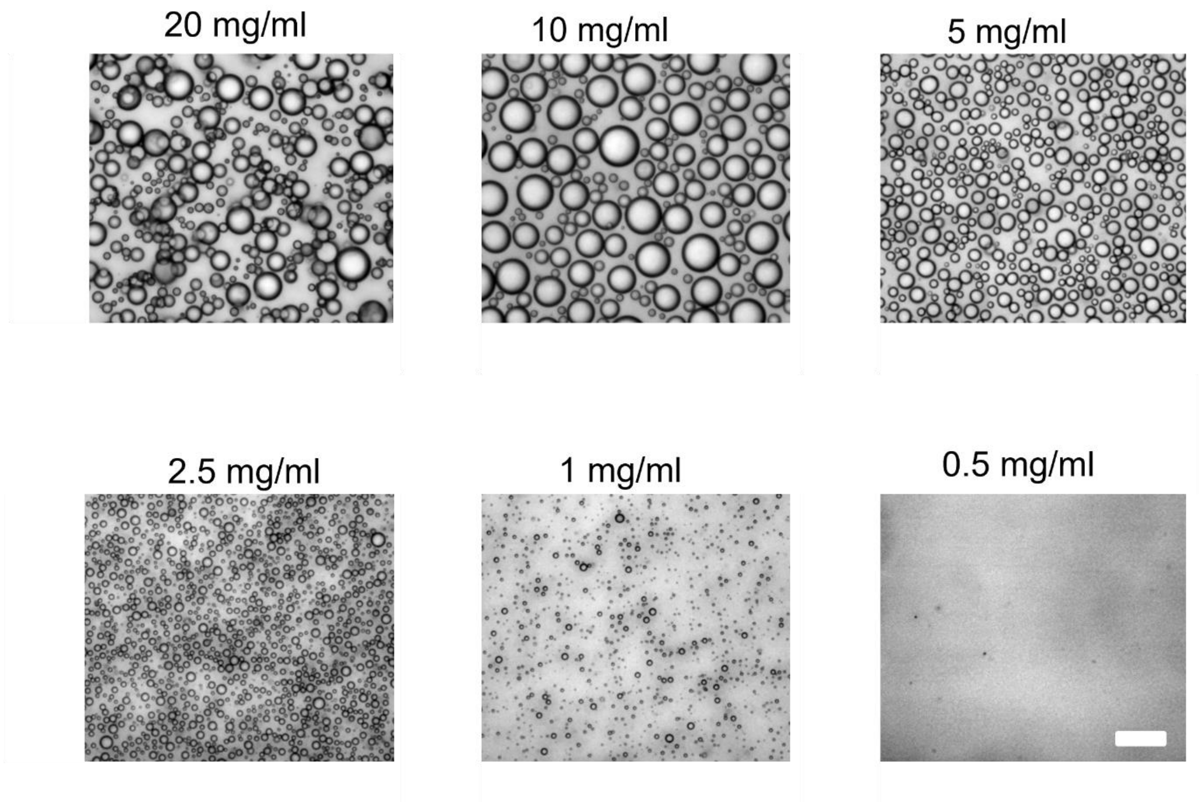

Supplementary Figure 46. Concentration dependent phase separation of FFM/MFF (1:1 eight ratio, in total concentration) at pH ~ 8. Based on microscopy imaging, the threshold concentration for coacervate formation is approximately 1 mg mL^{-1}, scale bar=20 μm in all images. Similar results were obtained with 3 samples measured independently.

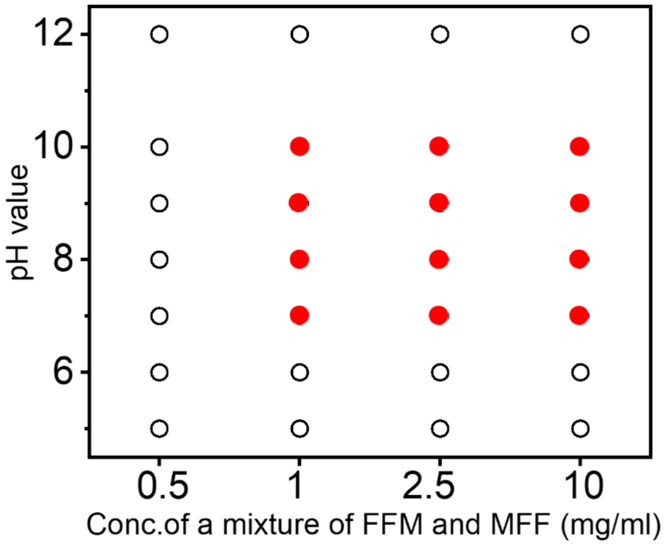

Supplementary Figure 47. Phase diagram of peptide coacervation for a mixture of FFM and MFF (1:1 weight ratio). Red dots represent conditions where coacervate droplets were observed. Empty circles indicate the absence of coacervate droplets.

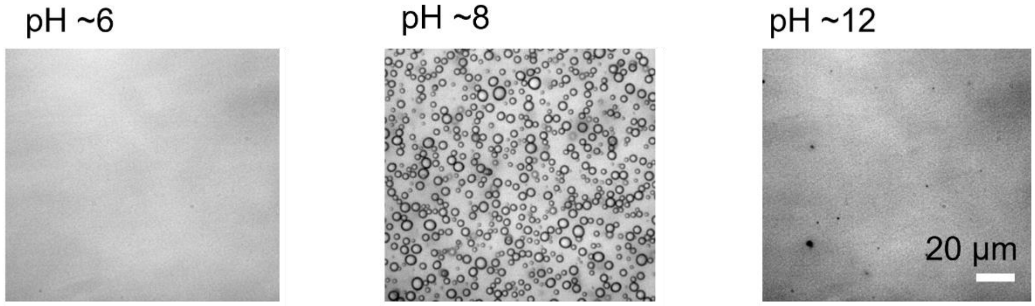

Supplementary Figure 48. pH-dependent phase separation of FFM/MFF. Initially, FFM/MFF peptides (1:1, 2.5 mg mL^{-1} each) are solubilized in pH 6 buffer, and an increase in pH to ~8 induces the formation of liquid droplets. However, a further increase in pH to ~12 results in the dissolution of the coacervates. Similar results were obtained with 3 samples measured independently.

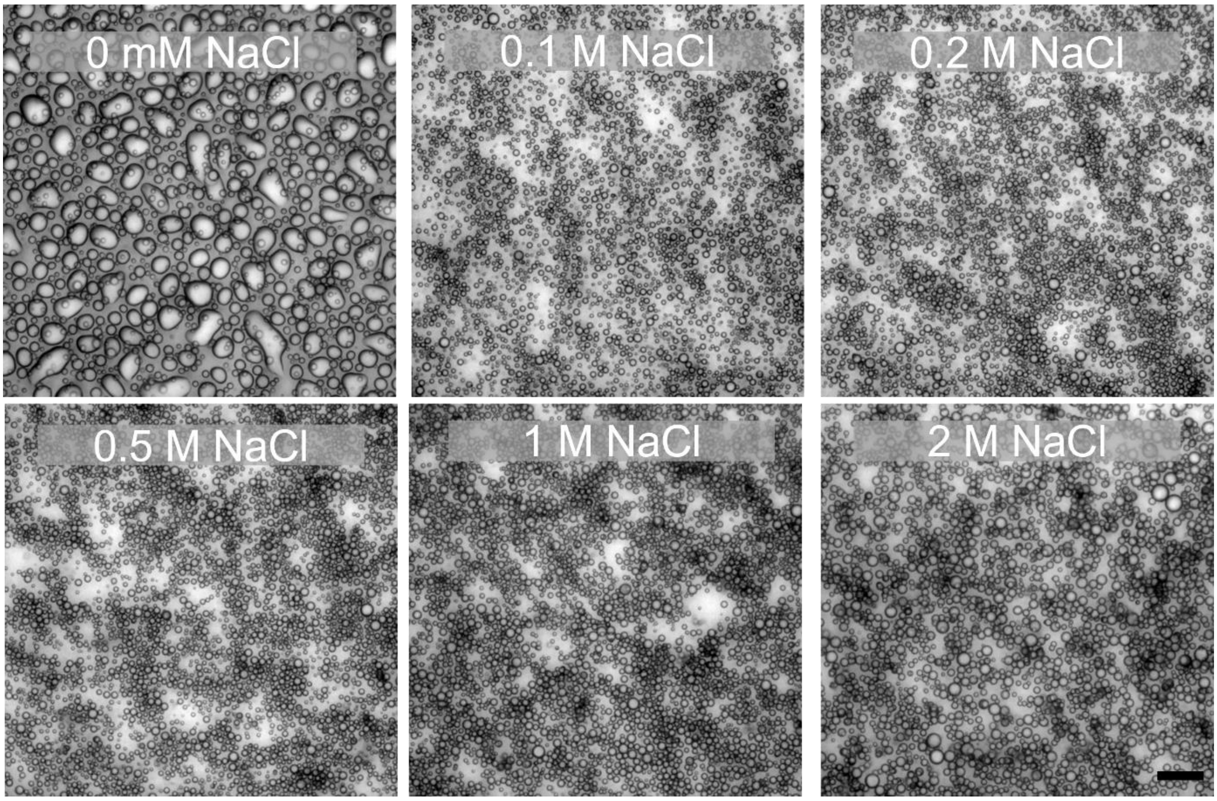

Supplementary Figure 49. The stability of FFM/MFF coacervates (1:1 weight ratio, in total 5 mg mL^{-1}) to electrolytes was tested by incubation with different concentrations of NaCl. The droplets remained condensed, with no solid transition observed, even in the presence of up to 2 M NaCl. Scale bar = 20 μm in all images.

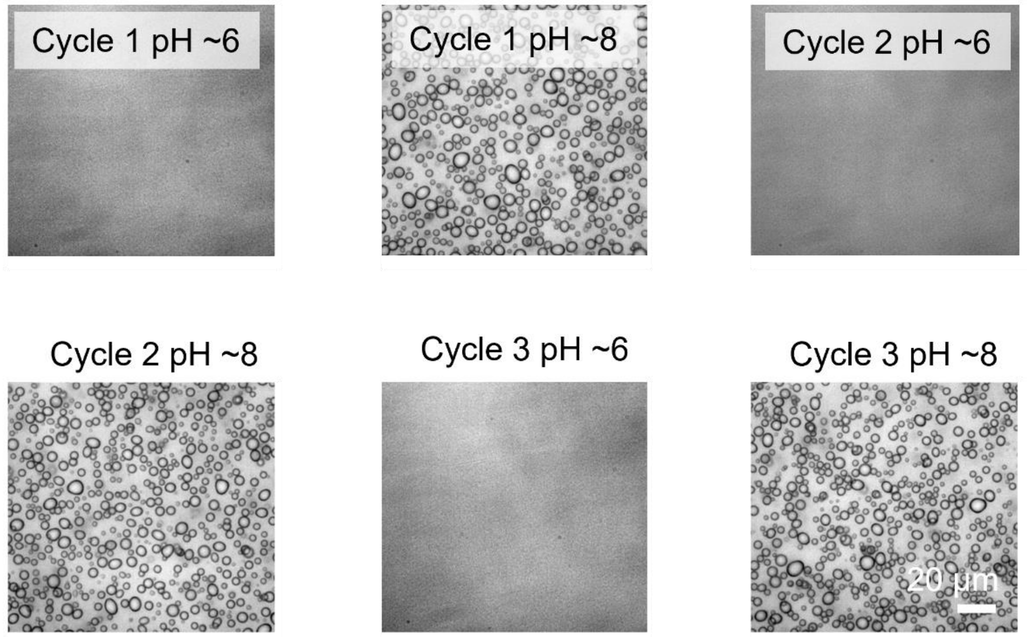

Supplementary Figure 50. pH reversible phase separation of FFM/MFF. Initially, the FFM/MFF peptides (1:1, 2.5 mg mL^{-1} each) are solubilized in pH 6 buffer. Increasing the pH to ~8 induces the formation of liquid droplets. The solubilization and formation of FFM/MFF coacervates can be repeated several times by sequentially decreasing or increasing the pH by adding a concentrated HCl or NaOH solution, scale bar=20 μm in all images.

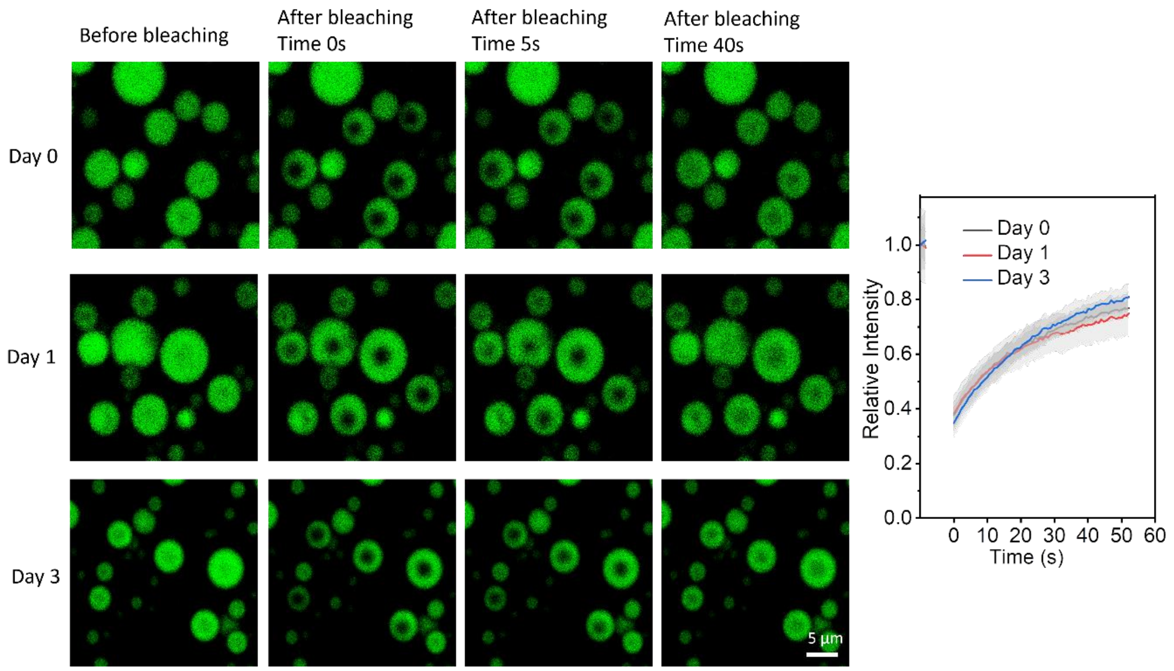

Supplementary Figure 51. Top: Confocal images corresponding to FRAP in MFF/FFM coacervates (1:1 weight ratio, 5 mg mL^{-1}) over time and after storage up to 3 days, the green emission is from rhodamine B. Scale bar = 5 µm. Similar results were obtained with 3 samples measured independently. Data represent mean ± SD for n = 5 representative microscopic images.

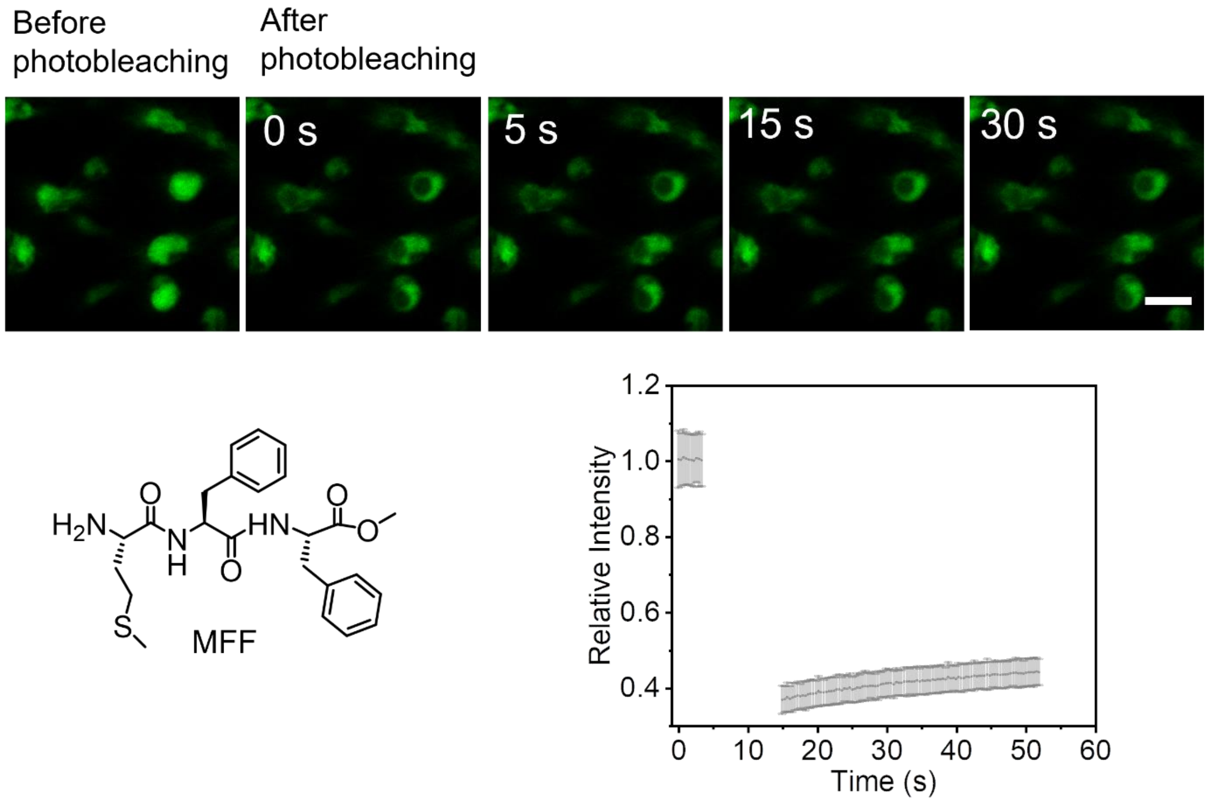

Supplementary Figure 52. Top: Confocal images corresponding to FRAP in MFF aggregates (5 mg mL^{-1}) over time, the green emission is Rhodamine B. Scale bar = 5 µm. Down: FRAP traces of MFF aggregates (5 mg mL^{-1}) over time. Similar results were obtained with 3 samples measured independently. Data represent mean ± SD for n = 5 representative microscopic images.

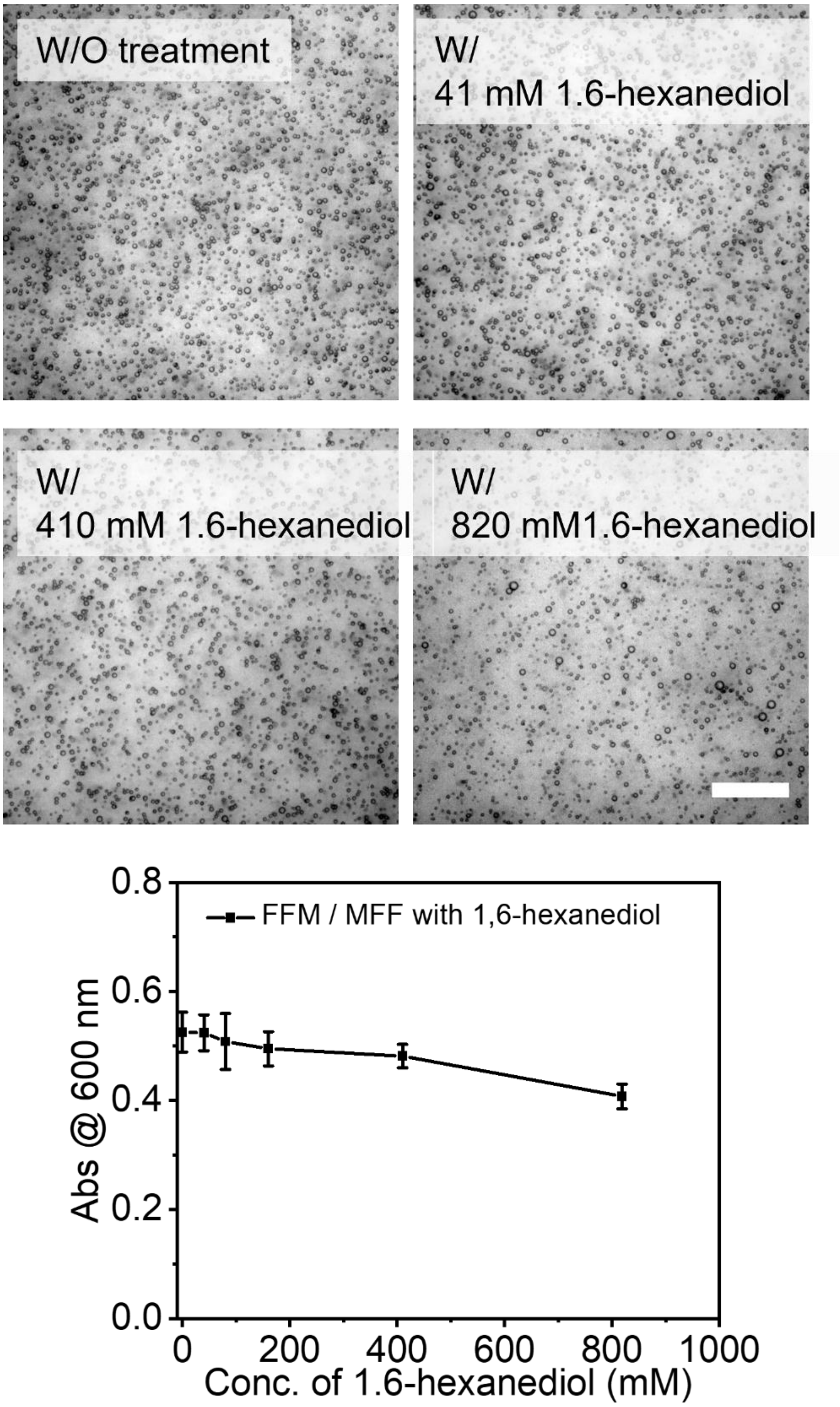

Supplementary Figure 53. Top: Phase separation behavior of FFM/MFF peptide (1:1 weight ratio, 5 mg mL^{-1}) in the presence of 1,6-hexanediol, scale bar= 50 μm in all images. Bottom: The turbidity of FFM/MFF coacervates showed a slight decrease in the presence of up to ~800 mM 1.6-hexanediol, indicating that hydrophobic interactions may not be the main effect driving the phase separation. Data represent the mean ± SD for n = 3 independent samples.

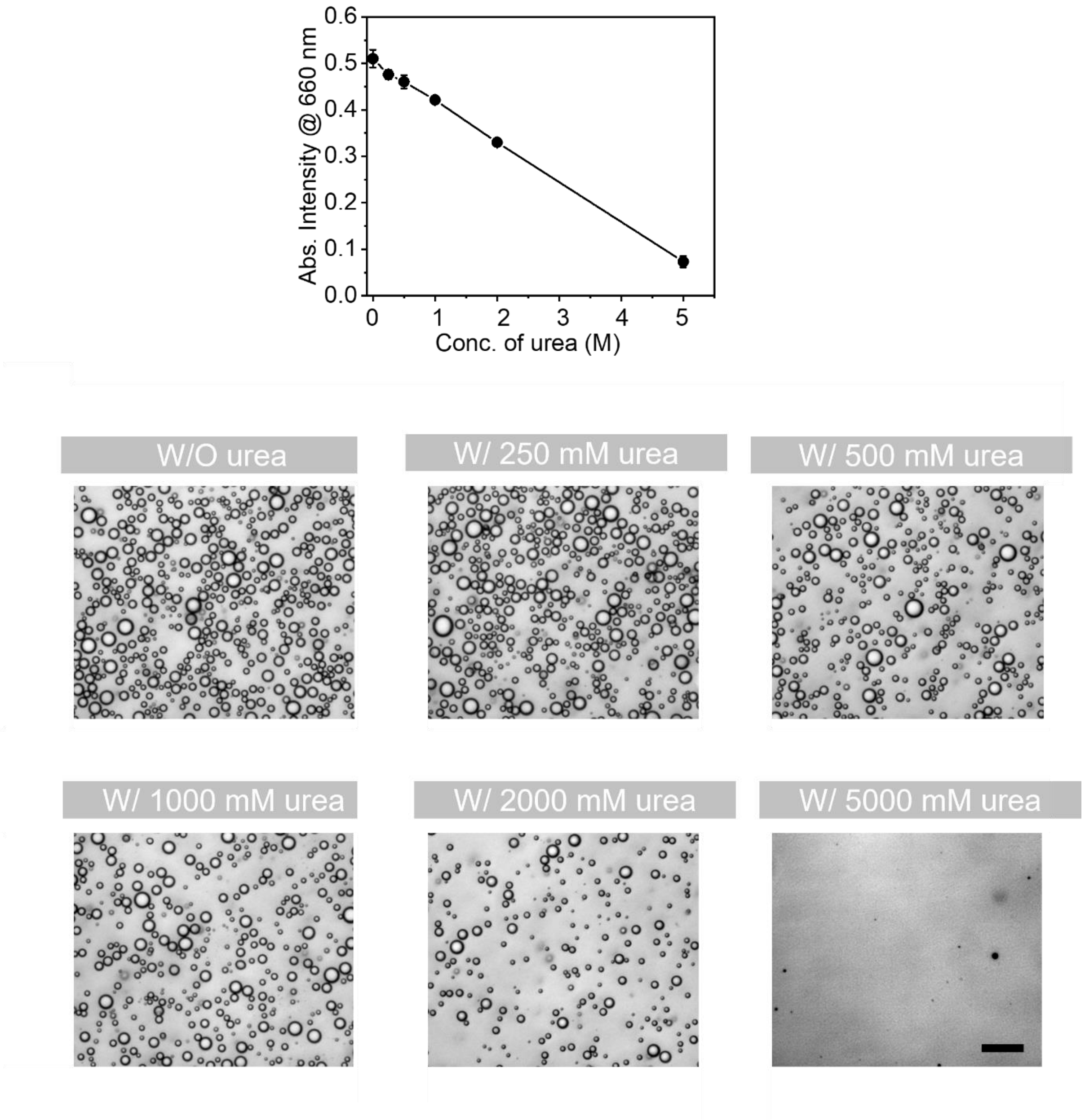

Supplementary Figure 54. The phase separation behavior of the FFM/MFF peptide (1:1 weight ratio, 5 mg mL^{-1}) was affected by the presence of high concentrations of urea. There is a clear decrease in turbidity with increasing urea concentration (top). Microscopic imaging shows that the density of peptide coacervates decreased with the addition of urea, which almost completely disappeared in the presence of 5 M urea. This indicates that the formation of FFM/MFF coacervates is associated with the intra/intermolecular hydrogen bonding interactions, scale bar= 20 μm in all images.

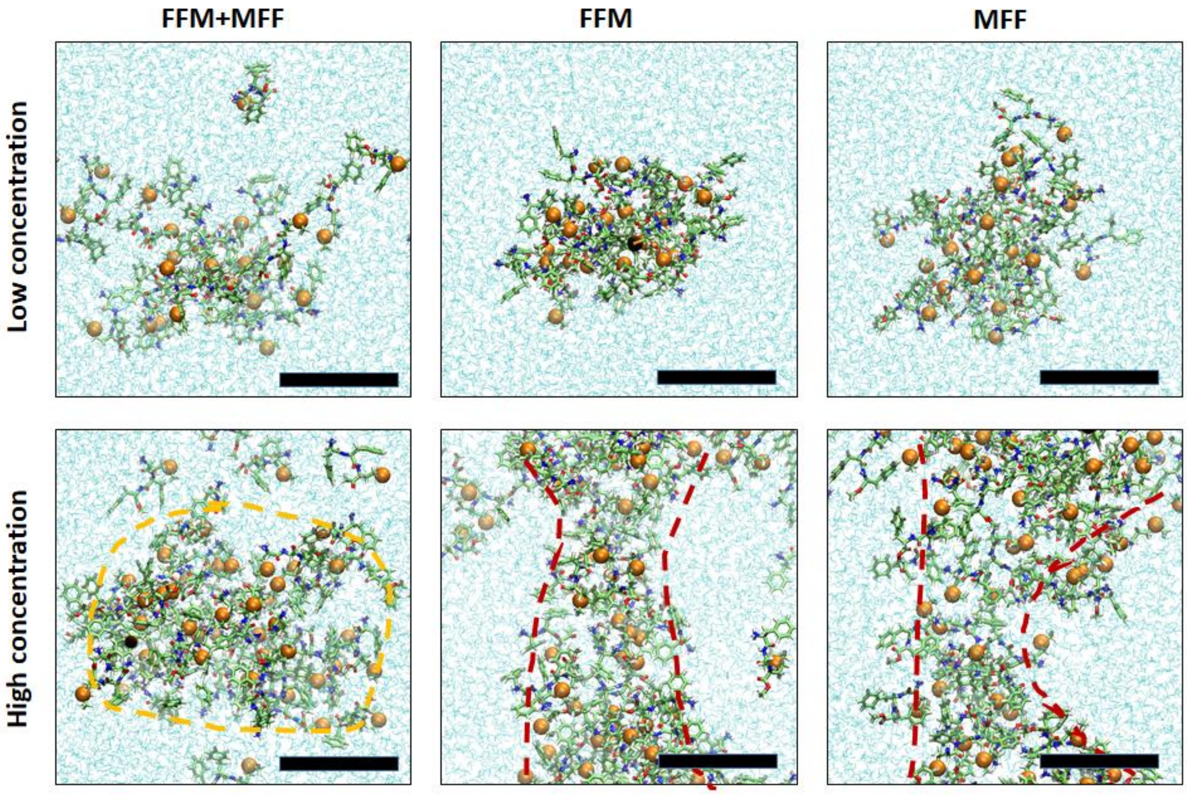

Supplementary Figure 55. Snapshots of the aggregation state of FFM+MFF, FFM only, and MFF only at low (80 mg mL^{-1}) and high (160 mg mL^{-1}) concentrations in the molecular dynamics (MD) simulations. It can be seen that pure FFM and pure MFF at high concentrations (160 mg mL^{-1}) can form fiber-like aggregates that run through the simulation boxes (shown in red dashed outline). The FFM/MFF mixture can only form loose aggregate (shown in orange dashed outline). The length scale bar is 2 nm.

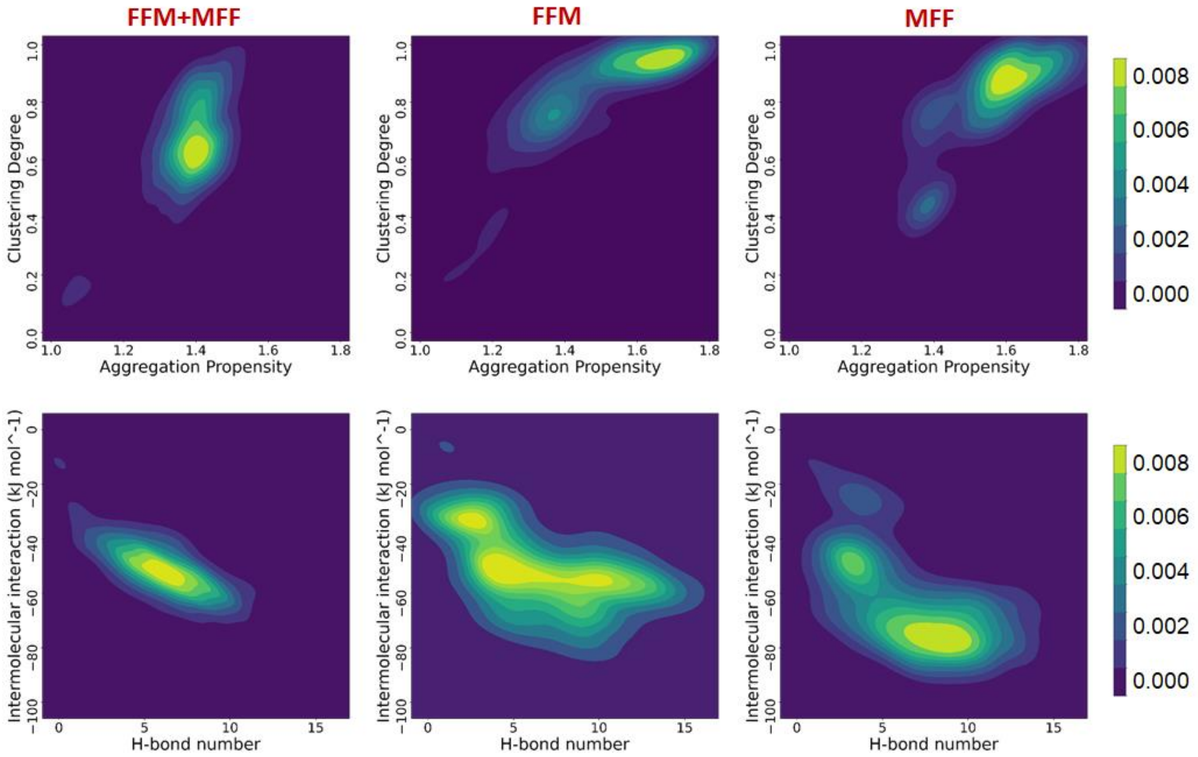

Supplementary Figure 56. Analysis of aggregation propensity, clustering degree, intermolecular H-bond number, and intermolecular interaction in the case of FFM+MFF, only FFM and only MFF with a low concentration (80 mg mL^{-1}) in the MD simulation. Top panel: clustering degree of the FFM+MFF mixture, pure FFM and pure MFF plotted as a function of their aggregation propensity values in the MD simulations. Bottom panel: intermolecular interaction values of the FFM+MFF mixture, pure FFM and pure MFF as a function of their intermolecular H-bond number values in the MD simulation. Pure FFM and pure MFF exhibit a high clustering degree (CD > 0.8) and a high aggregation propensity (AP > 1.6). The FFM+MFF mixture exhibits a low clustering degree (0.6-0.7) and a low aggregation propensity (~1.4). The color scale bar representing the probability density along the z-axis is displayed on right.

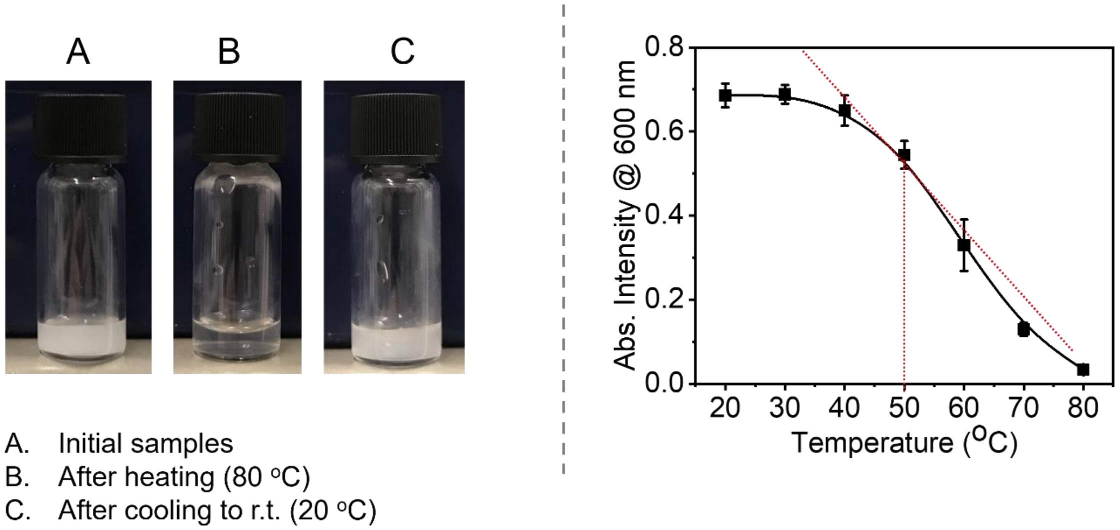

Supplementary Figure 57. Response of FFM/MFF coacervates (1:1 weight ratio, 5 mg mL^{-1}) to thermal heating. Left: The cloudy coacervate solution became clear after heating to 80°C, which returned to the cloudy state after cooling to room temperature. Right: Cloudiness of FFM/MFF coacervates at different temperatures, where the transition temperature is estimated to be ~ 50°C. Data represent the mean ± SD for n = 3 independent samples.

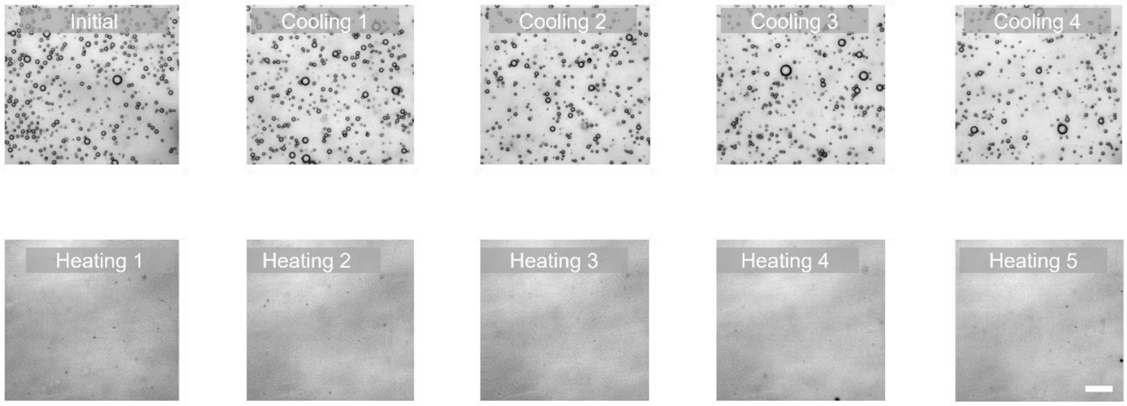

Supplementary Figure 58. Microscopic imaging showed the response of FFM/MFF coacervates (1:1 weight ratio, 5 mg mL^{-1}) to thermal heating, scale bar=20 μm in all images.

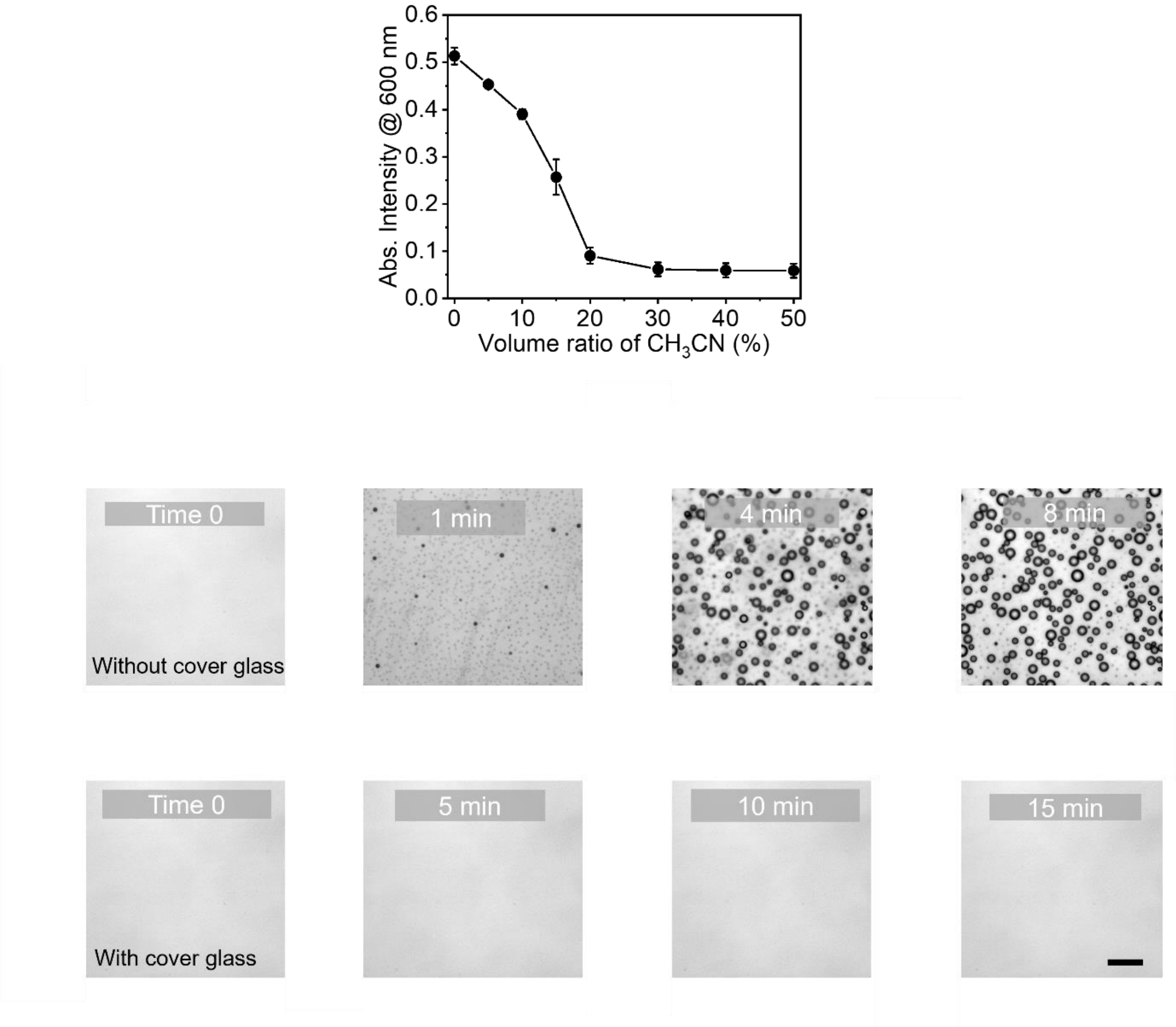

Supplementary Figure 59. The phase separation behavior of FFM/MFF peptide (1:1 weight ratio, in total 5 mg mL^{-1}) in the presence of organic solvent. Turbidity of FFM/MFF peptide (1:1 weight ratio, in total 5 mg mL^{-1}) in the presence of different volumes of CH_3CN. The turbidity showed a sharp decrease when treated with more than 20% CH_3CN (volume ratio). Top row: Coacervates (in 5 mM HEPES) were dissolved by adding 50% volume of CH_3CN. Evaporation of CH_3CN resulted in the formation of liquid coacervates again. Data represent the mean ± SD for n = 3 independent samples. Bottom row: However, with a cover glass to inhibit solvent evaporation, the solution remains transparent, and no droplets are observed during prolonged incubation, scale bar= 20 μm in all images. Similar results were obtained with 3 samples measured independently.

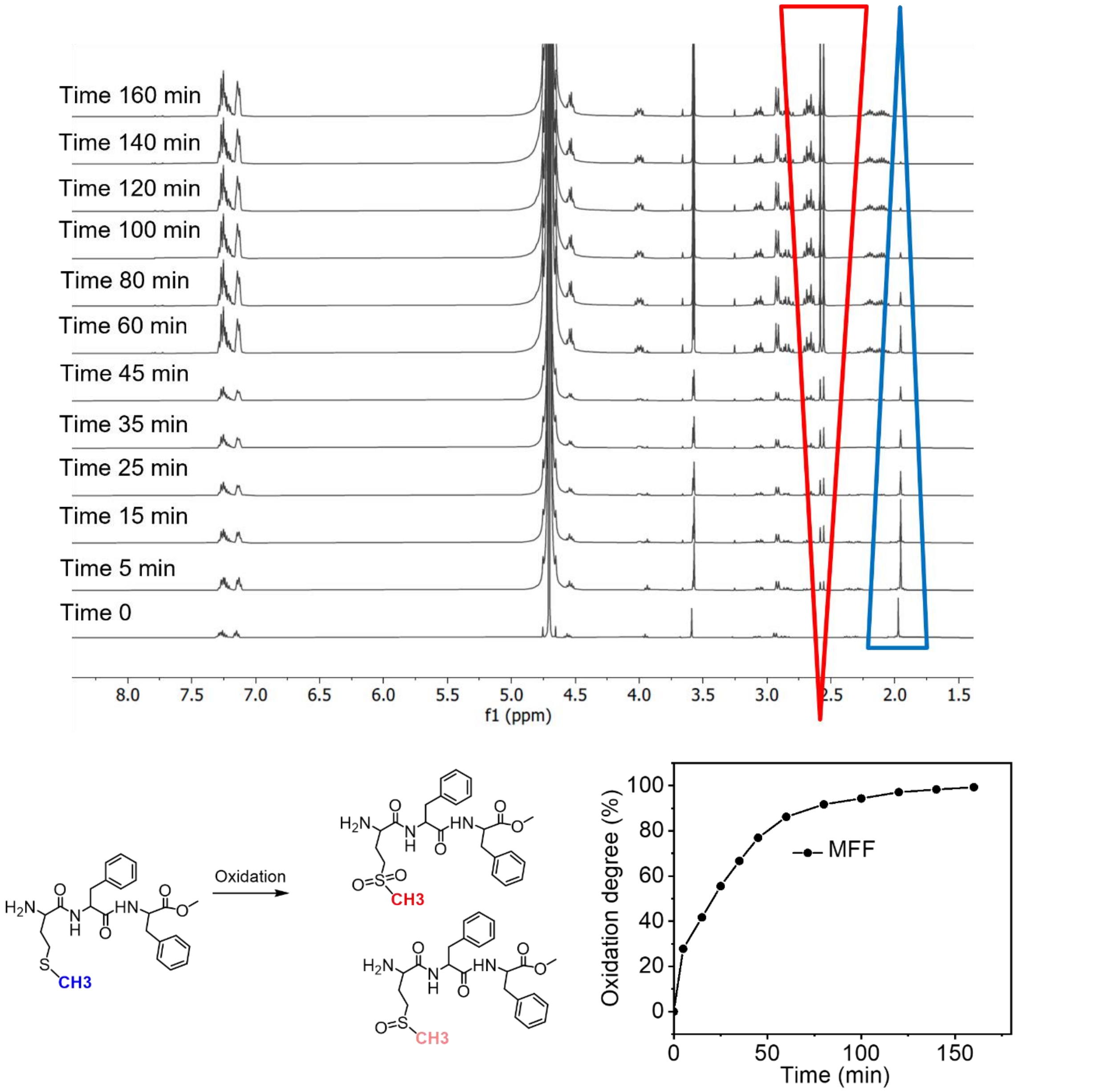

Supplementary Figure 60. Top: HNMR measurements of MFF (5 mg mL^{-1}) incubated with 0.5% H_2O_2 over time. The -S-CH_3 peak in MFF (marked in blue) gradually decreases, while the CH_3 peak in the oxidized product (marked in red) gradually increases. Bottom: Chemical scheme of the MFF oxidation together with the calculated degree of oxidation by comparing the protons in MFF and the oxidized product.

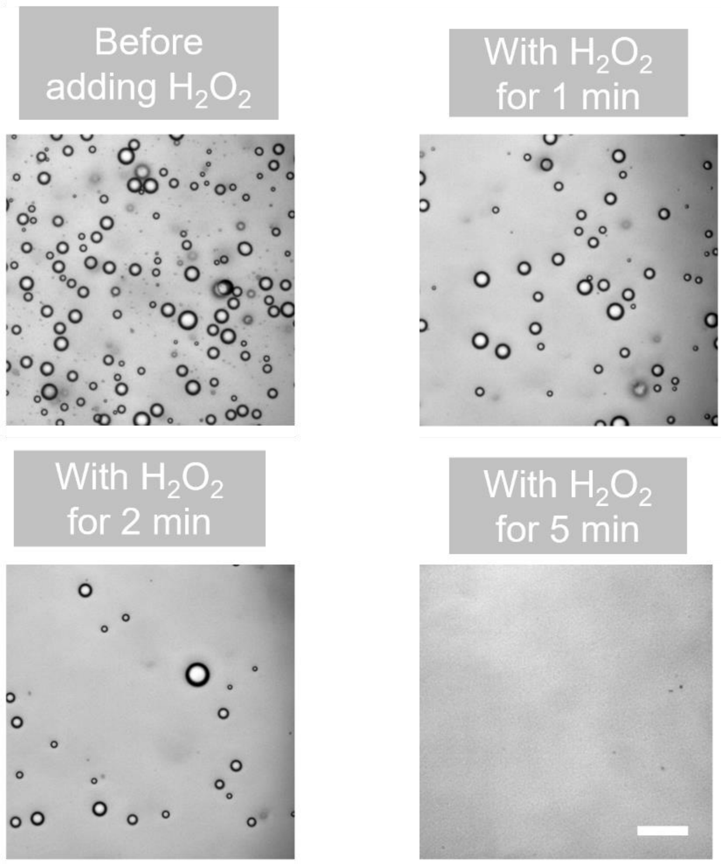

Supplementary Figure 61. Microscopic imaging illustrating the responsiveness of FFM/MFF coacervates (1:1 weight ratio, total 5 mg mL^{-1}) to 1% H_2O_2. The initial BPCs gradually dissolved in the medium upon exposure to 1% H_2O_2. Scale bar=20 μm in all images. Similar results were obtained with 3 independently measured samples.

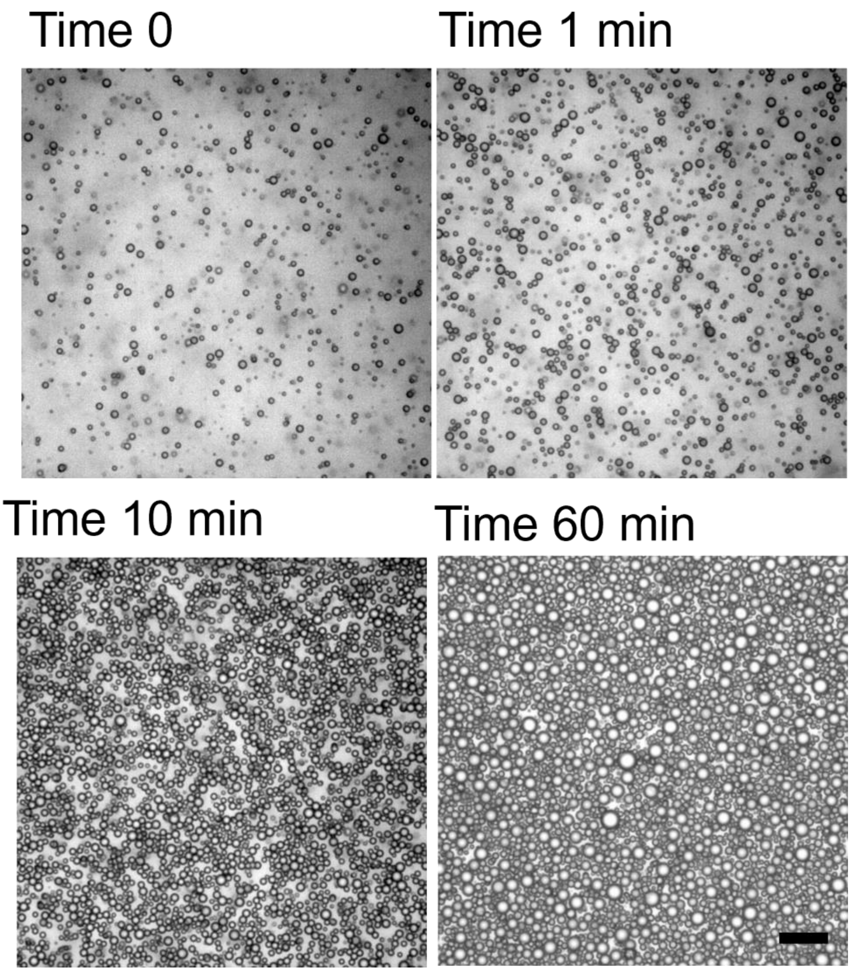

Supplementary Figure 62. Microscopic imaging revealed that in the absence of H_2O_2, the FFM/FFF coacervates (1:1 weight ratio, 5 mg mL^{-1}) maintain a condensed state, with no fiber transition observed after 60 min observation, scale bar=20 μm in all images.

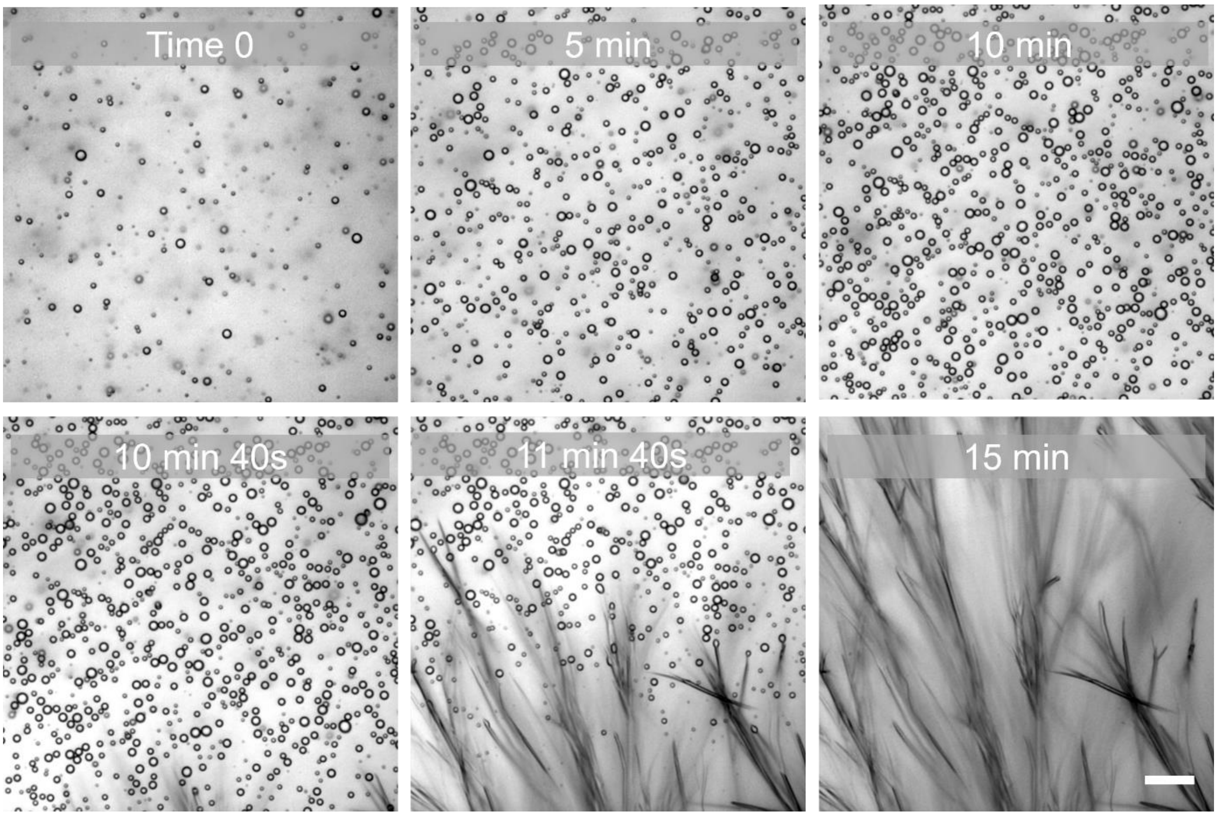

Supplementary Figure 63. Microscopic imaging revealed the response of the FFM/FFF coacervates (1:1, in total 5 mg mL^{-1}) to the presence of 1% H_2O_2. In the presence of H_2O_2, the BPCs showed a gradual solid transition that produced a fibrous structure after ~10 min of incubation. Within minutes, all BPCs were transformed into a solid fibrous structure, scale bar=20 μm in all images. Similar results were obtained with 3 samples measured independently.

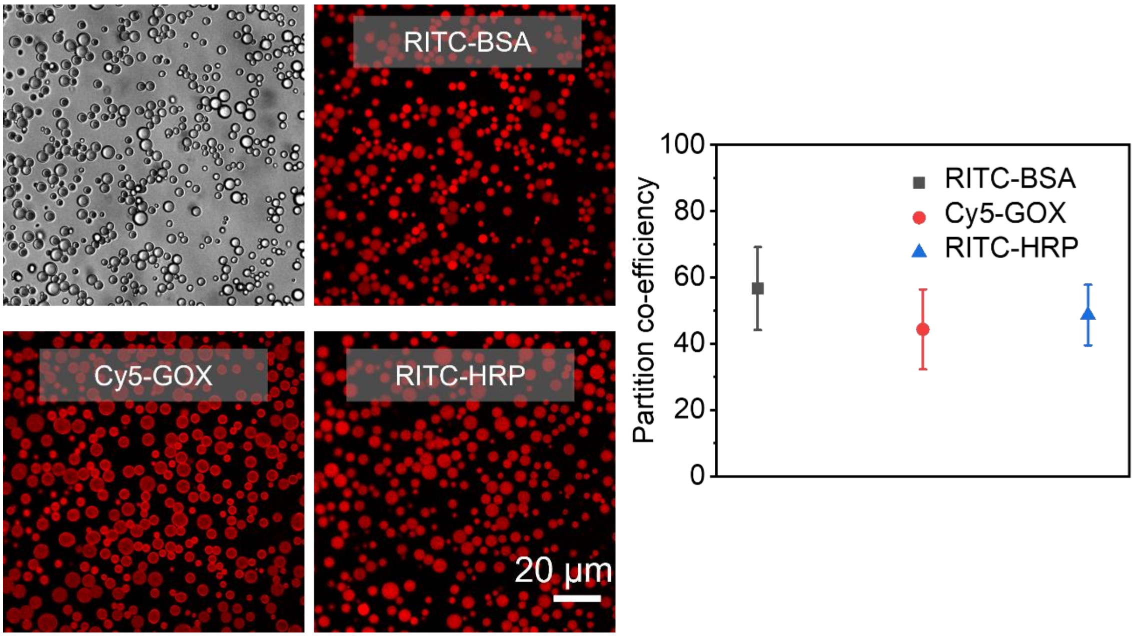

Supplementary Figure 64. Partitioning of FFM/MFF peptide-coacervates towards various proteins and enzymes. Confocal imaging analysis showed the peptide-coacervates are able to partition various proteins and enzymes, scale bar=20 μm. Similar results were obtained with 3 samples measured independently. Data represent mean ± SD for n = 5 representative microscopic regions of interest.

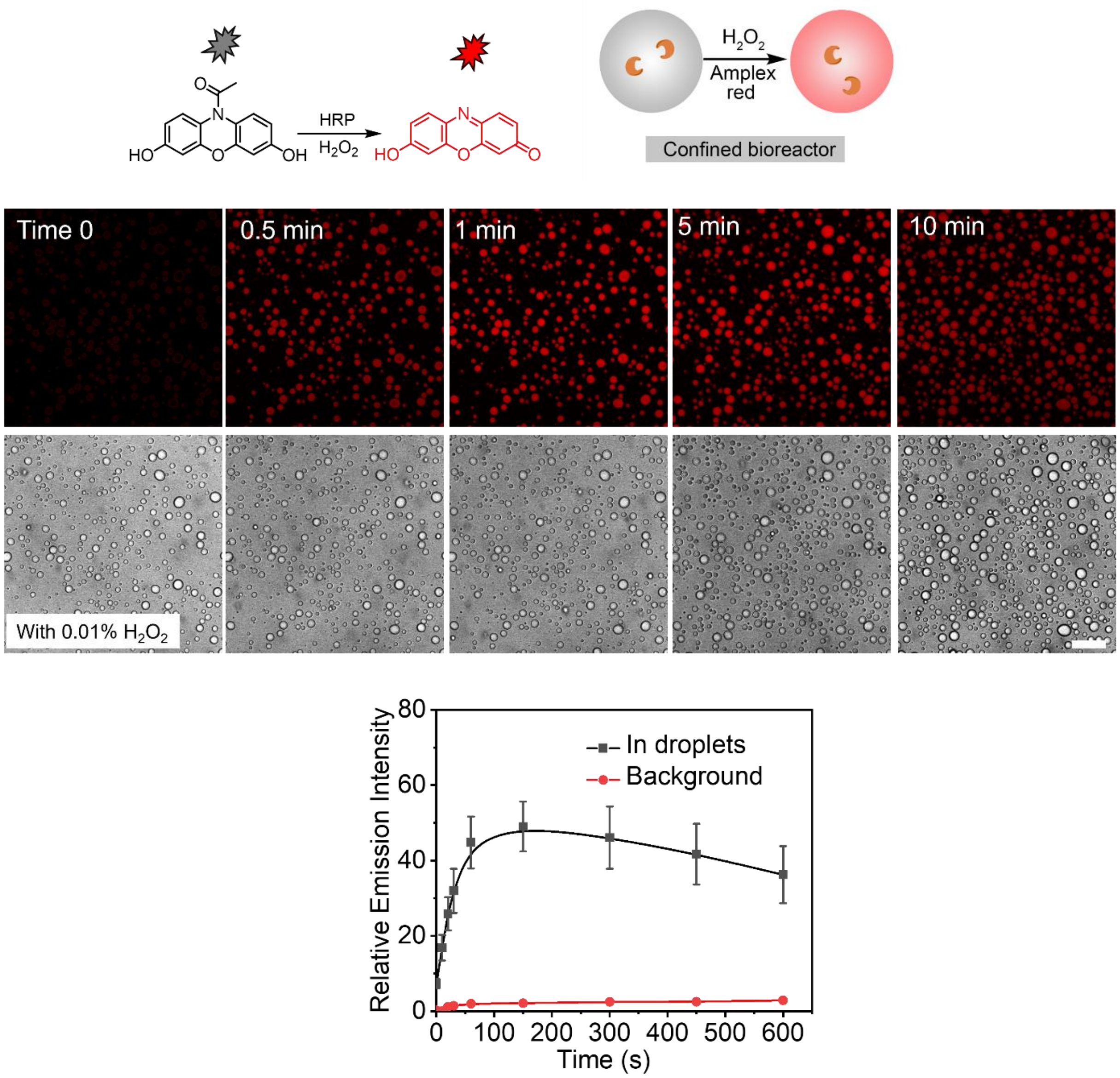

Supplementary Figure 65. Top: Schematic illustration of enzymatic microreactors obtained with BPCs (FFM/MFF)) and HRP. The presence of H_2O_2 and HRP catalyzes the conversion of non-fluorescent Amplex-red to fluorescent Resofurin; Middle: Confocal imaging of the HRP-integrated BPCs and Amplex-red in the presence of 0.01% H_2O_2 after 10 min, scale bar: 20 μm in all images; Bottom: Quantification of fluorescence intensity inside the droplets and in the background. The emission inside the droplets reaches a plateau. Similar results were obtained with 3 samples measured independently. Data represent mean ± SD for n = 5 representative microscopic regions of interest.

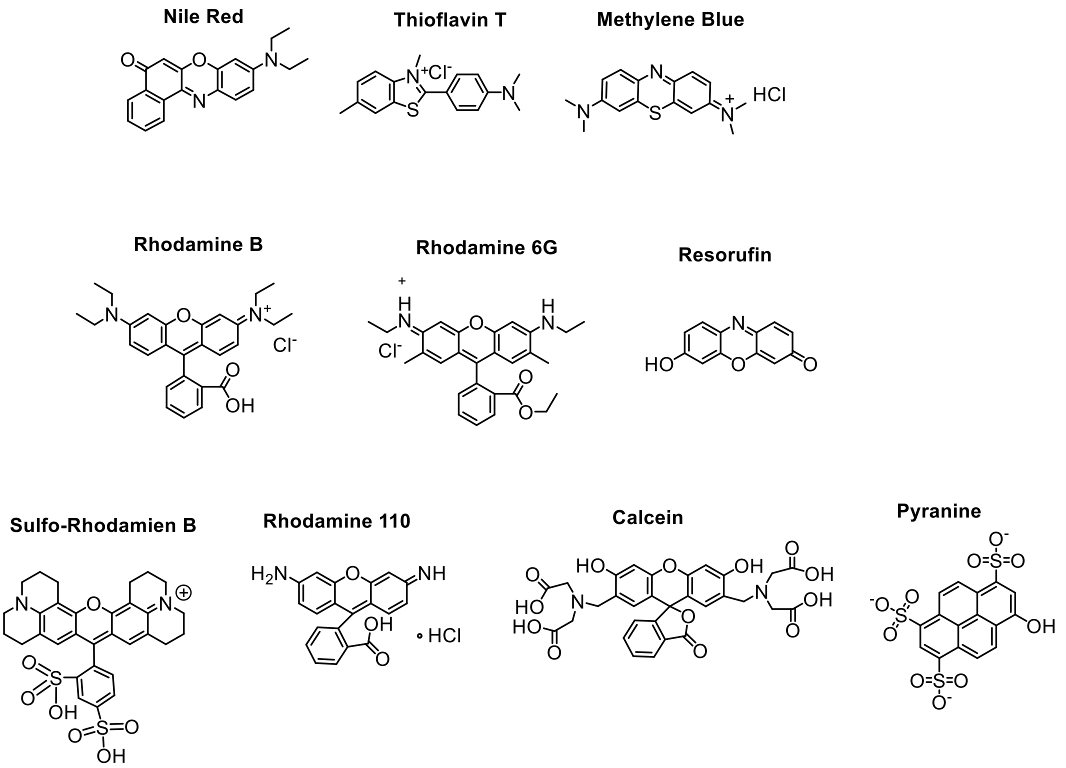

Supplementary Figure 66. Dyes used in the FFM/MFF coacervate partitioning test

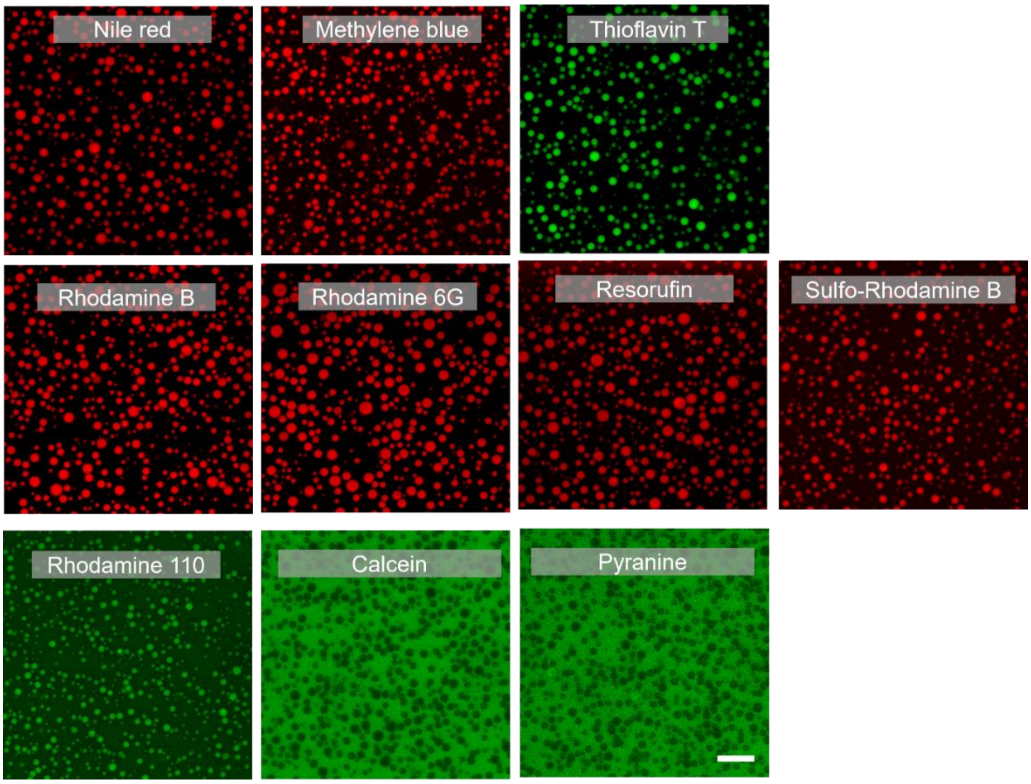

Supplementary Figure 67. Confocal images of FFM/MFF coacervates (1:1 weight ratio, 5 mg mL^{-1}) in the presence of different fluorescent dyes (0.02 mg mL^{-1}), scale bar=20 μm in all images. Similar results were obtained with 3 samples measured independently.

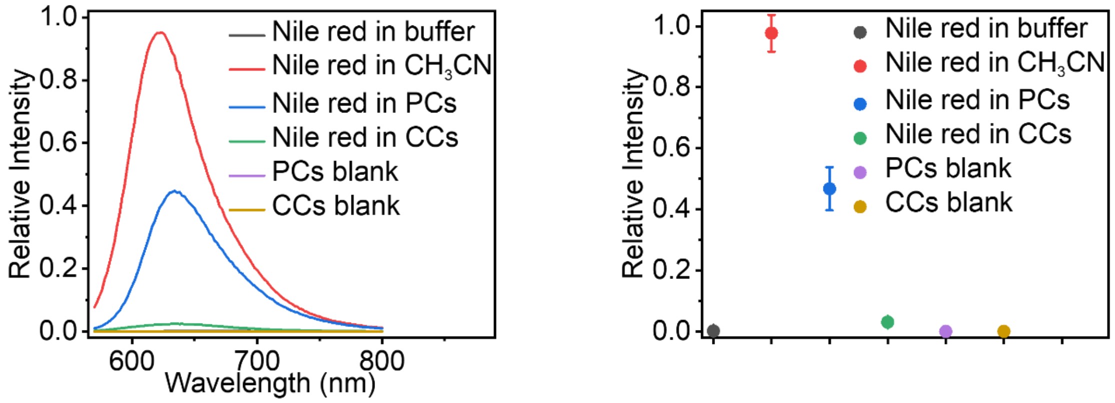

Supplementary Figure 68. Fluorescent emission curve (left) and intensity (right) of Nile red (0.05 mg mL^{-1}) dispersed in HEPES buffer, CH_3CN, peptide condensates (PCs), complex coacervates (CCs), as well as PCs and CCs blank solution. Data represent mean ± SD for n = 3 independent samples.

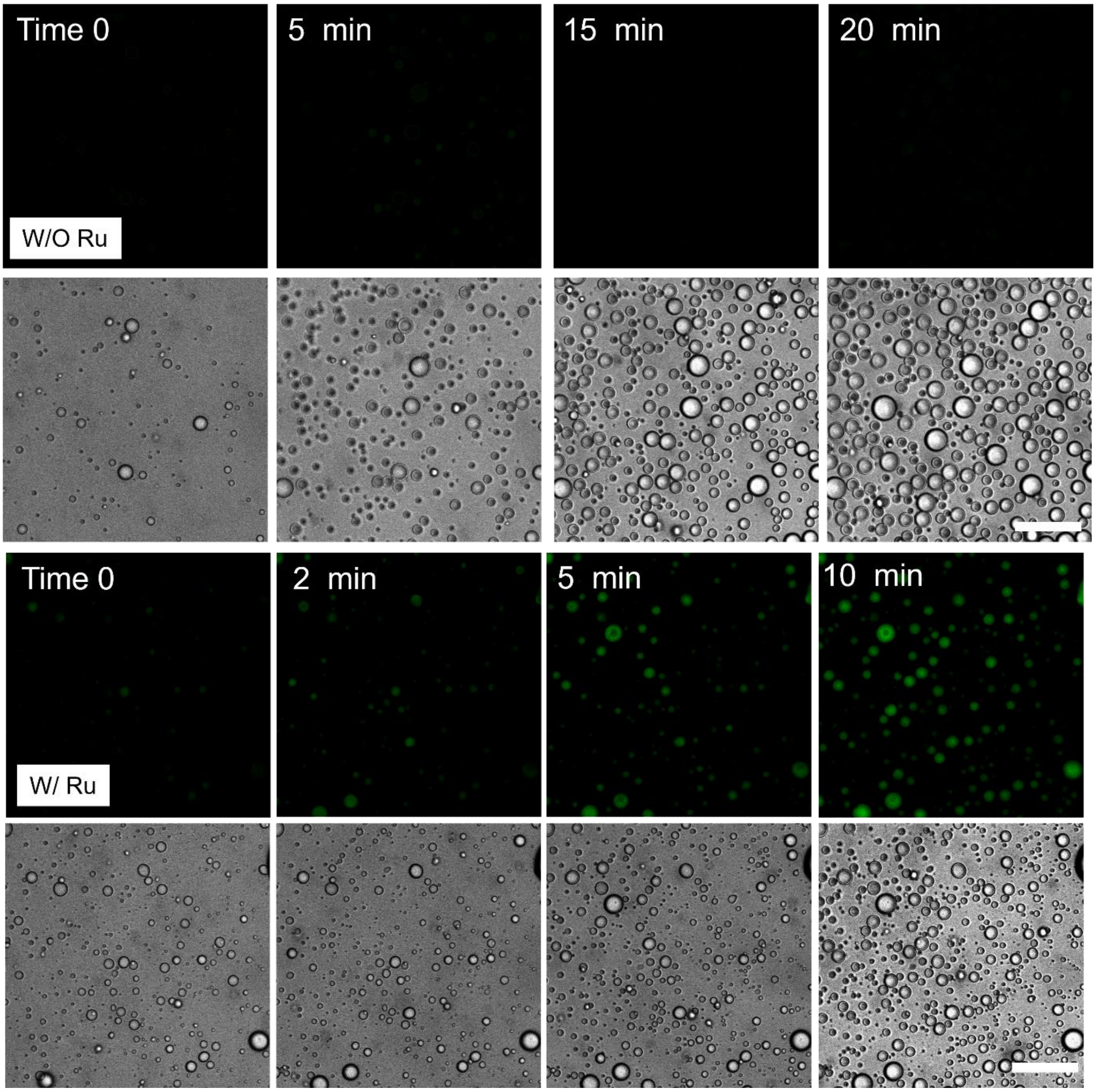

Supplementary Figure 69. Confocal imaging of peptide condensates (FFM/MFF, 1:1 weight ratio, 5 mg mL^{-1}) utilized in the Ru-catalyzed decaging of Rho-pro. Top: without Ru, there is no emission increase in the peptide condensates after prolonged incubation time. Down: When the peptide condensates are integrated with Ru, green emission are gradually produced with the coacervates, scale bar: 20 μm in all images. Similar results were obtained with 3 samples measured independently.

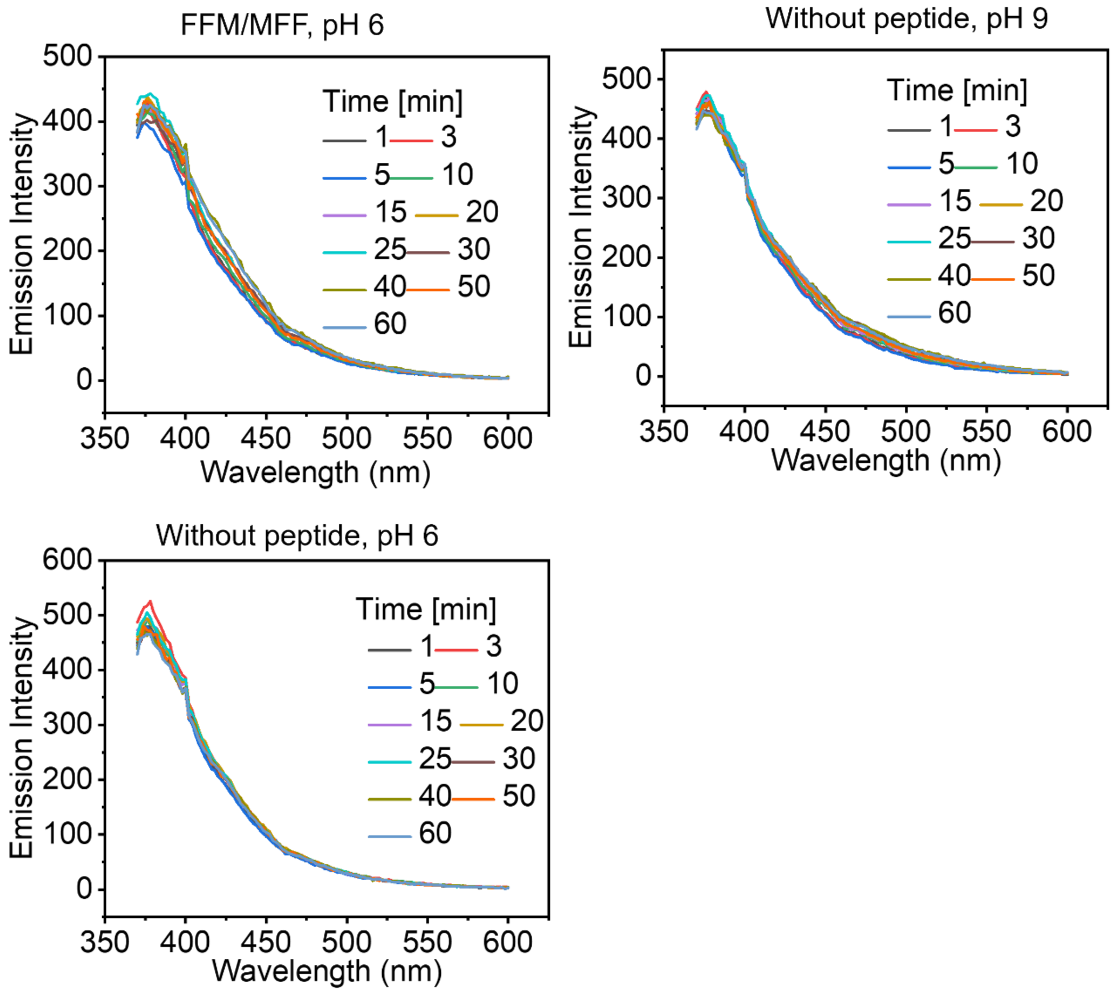

Supplementary Figure 70. Fluorescence emission curve of the control groups in the CuAAC reactions, including groups with only FFM/MFF peptide solution at pH 6, without peptide at pH 6, and without peptide at pH 9. The Cu(I) catalyst was present in all cases. There is almost no increase in product emission intensity at λ_{em}=451 nm.

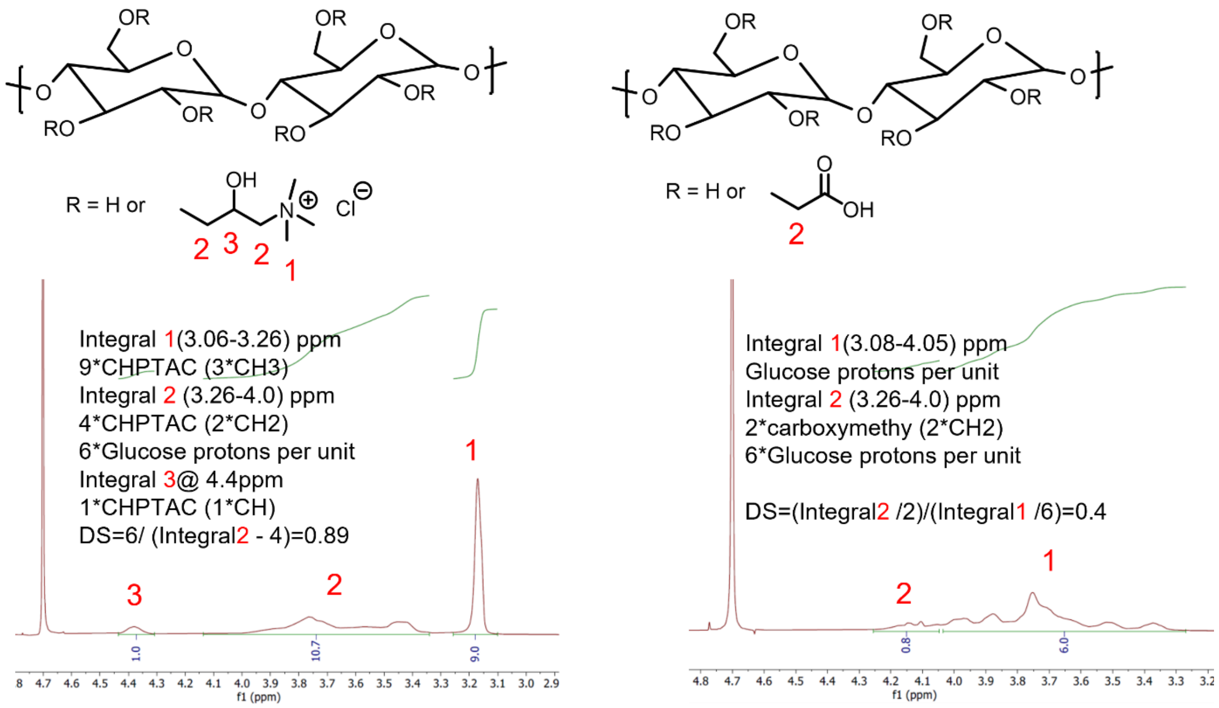

Supplementary Figure 71. ^{1}H NMR spectra of charged amylose derivatives and the calculation of the degree of substitution (DS = number of modifying groups per glucose unit), of both quaternized and carboxymethylated amylose. In the left figure CHPTAC refers to 3-chloro-2-hydroxypropyltrimethylammonium chloride, which was used as a reagent in the substitution reaction to prepare quaternized amylose.

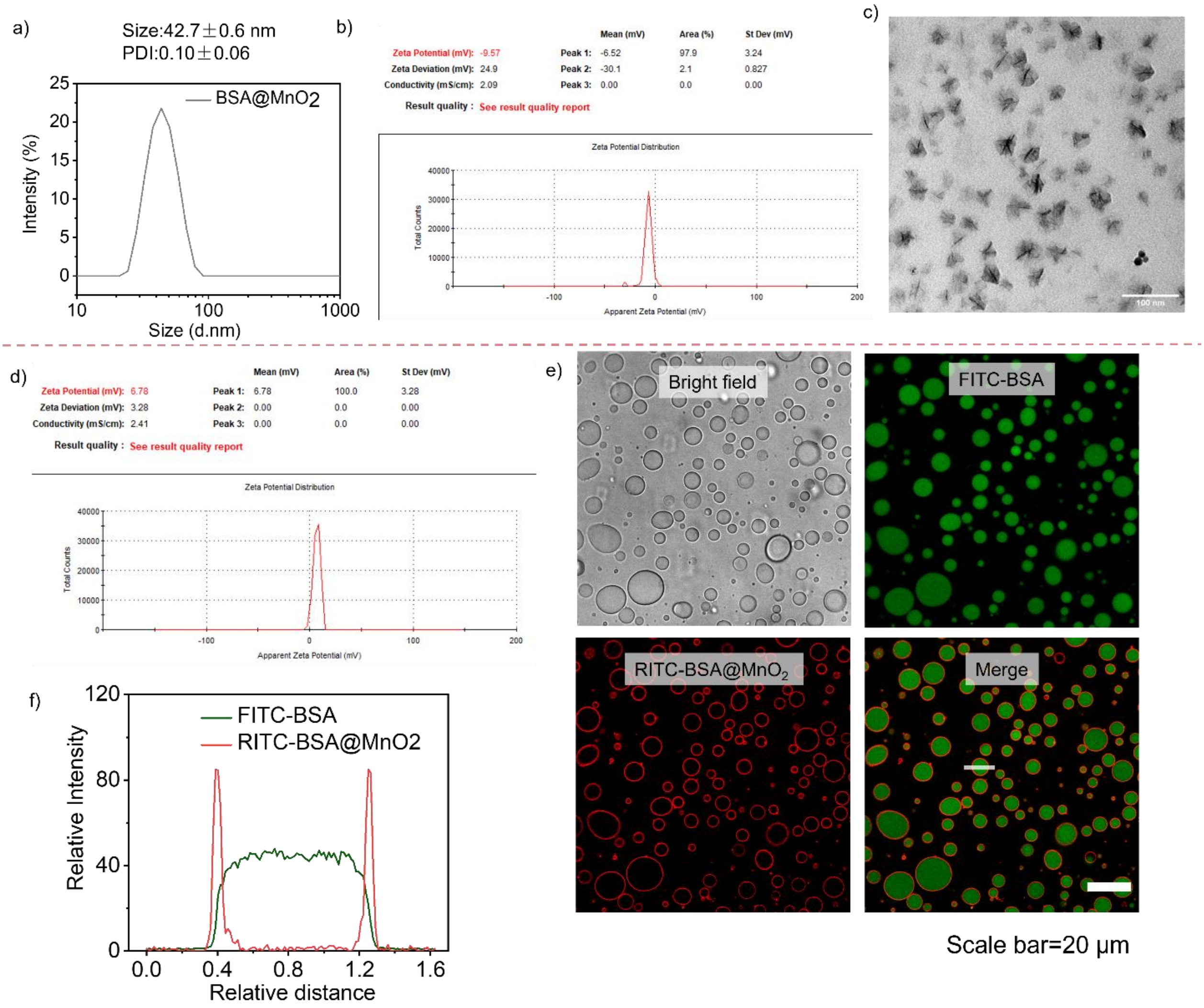

Supplementary Figure 72. Top: a) DLS, b) zeta potential measurement, and c) TEM image of BSA@MnO_2, which showed an average size of 40 nm and negative charge of -11.0+2.5 mV, scale bar=100 nm. Bottom: d) Zeta potential of unstabilized coacervates, which showed a positive charge of 6.03±0.73 mV. e) BSA@MnO_2 stabilized complex coacervate formed by the association between Q-AM and C-AM, scale bar=20 μm. In confocal imaging, BSA@MnO_2 particles are mainly located on the surface of the complex coacervates, while BSA is sequestered inside the complex coacervates, as verified by line profile analysis of fluorescence intensity, as shown in f). Similar results were obtained with 3 samples measured independently.

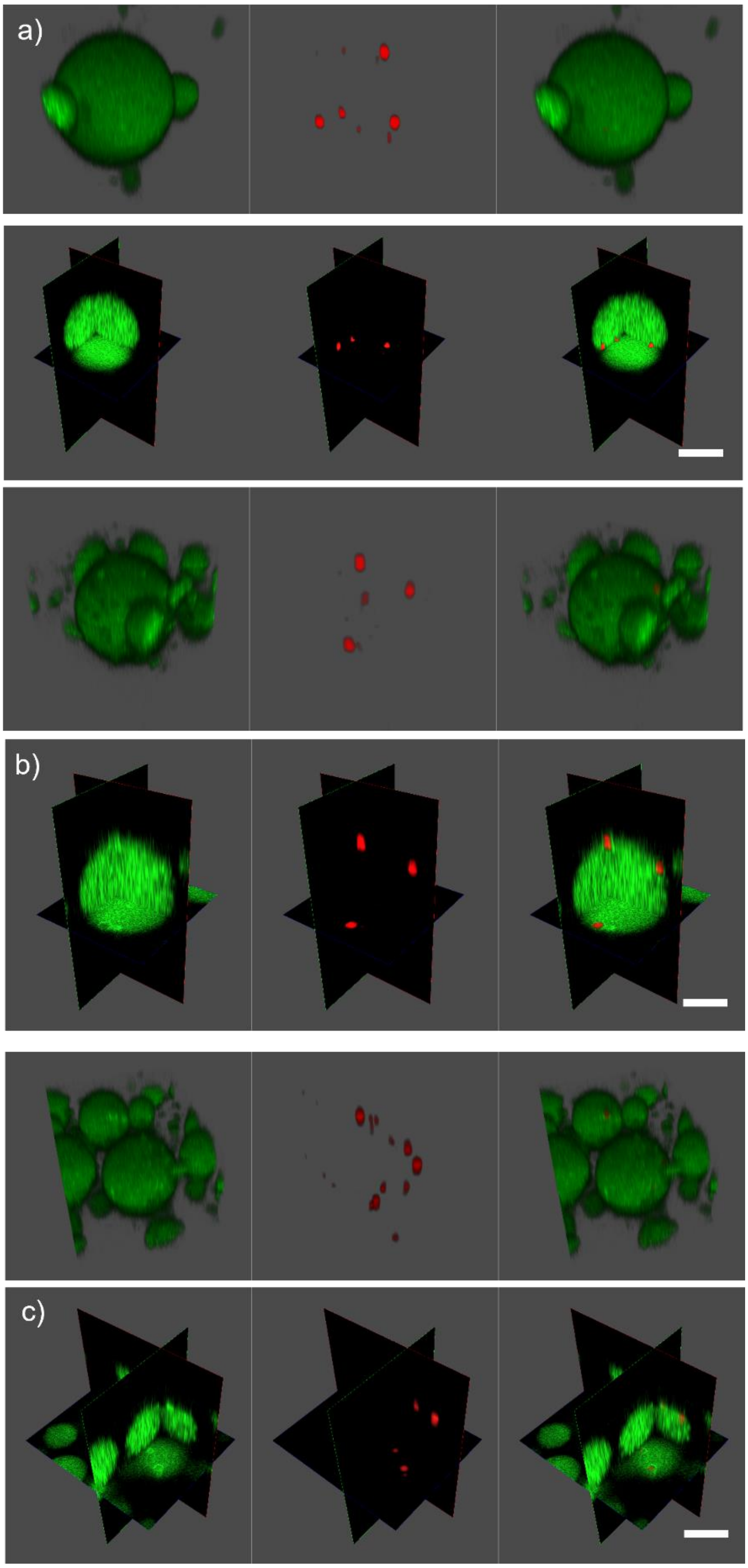

Supplementary Figure 73. 3D cross-sectional images and reconstruction images of multi-compartment artificial cells (MACs) formed by integrating BPCs into membrane-bound complex coacervates. Green channel: FITC-BSA; red channel: Nile Red, scale bar=5 μm in all images. Similar results were obtained with 3 samples measured independently.

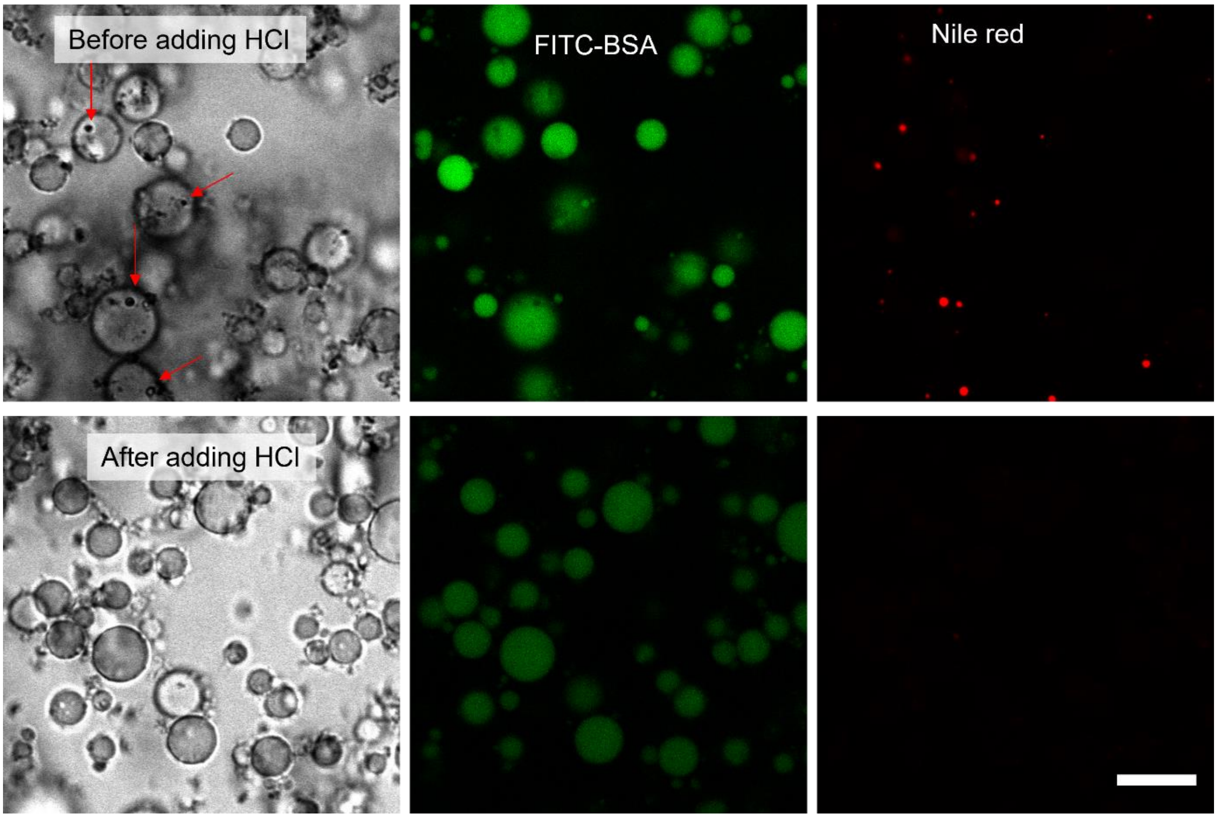

Supplementary Figure 74. Confocal images revealed that the presence of H^+ can dissolve the BPCs in OR-MACs. Before HCl treatment, internal BPCs with Nile red emission are observed inside MACs. After HCl treatment to pH~5 (observed with pH paper), BPCs disappeared and there was nearly no emission of Nile red, the scale bar=20 μm in all images. Similar results were obtained with 3 samples measured independently.

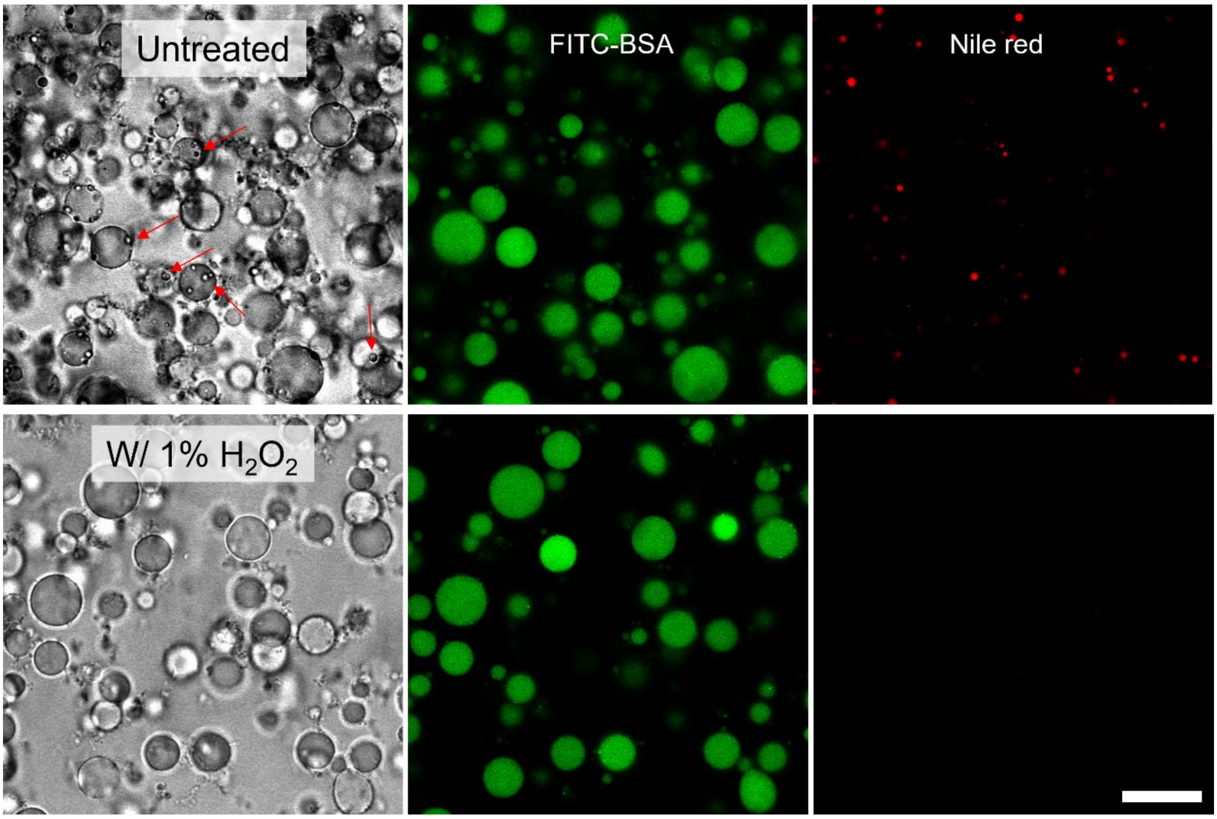

Supplementary Figure 75. Confocal images revealed that the presence of H_2O_2 can dissolve the BPCs in MACs. Before treatment, BPCs with Nile red emission are observed inside OR-MACs. After treatment with 1% H_2O_2, the BPCs disappeared and there was nearly no emission of Nile red, the scale bar=20 μm in all images. Similar results were obtained with 3 samples measured independently.

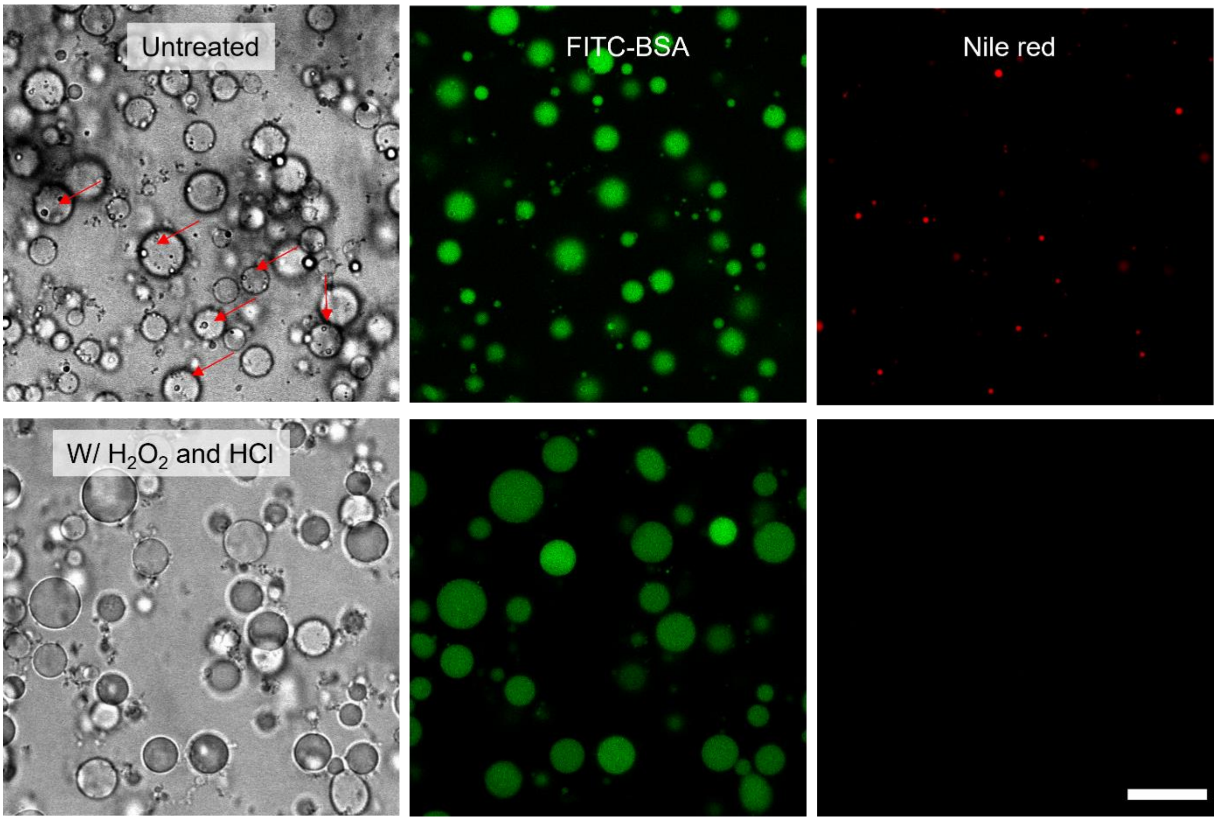

Supplementary Figure 76. Confocal images revealed that the presence of H_2O_2 and H^+ is able to dissolve the BPCs in OR-MACs. Before treatment, BPCs with Nile red emission are clearly observed inside OR-MACs. While after treatment with 1% H_2O_2 and HCl (to pH~5, observed with pH paper), the BPCs disappeared and there was nearly no emission of Nile red, the scale bar=20 μm in all images. Similar results were obtained with 3 samples measured independently.

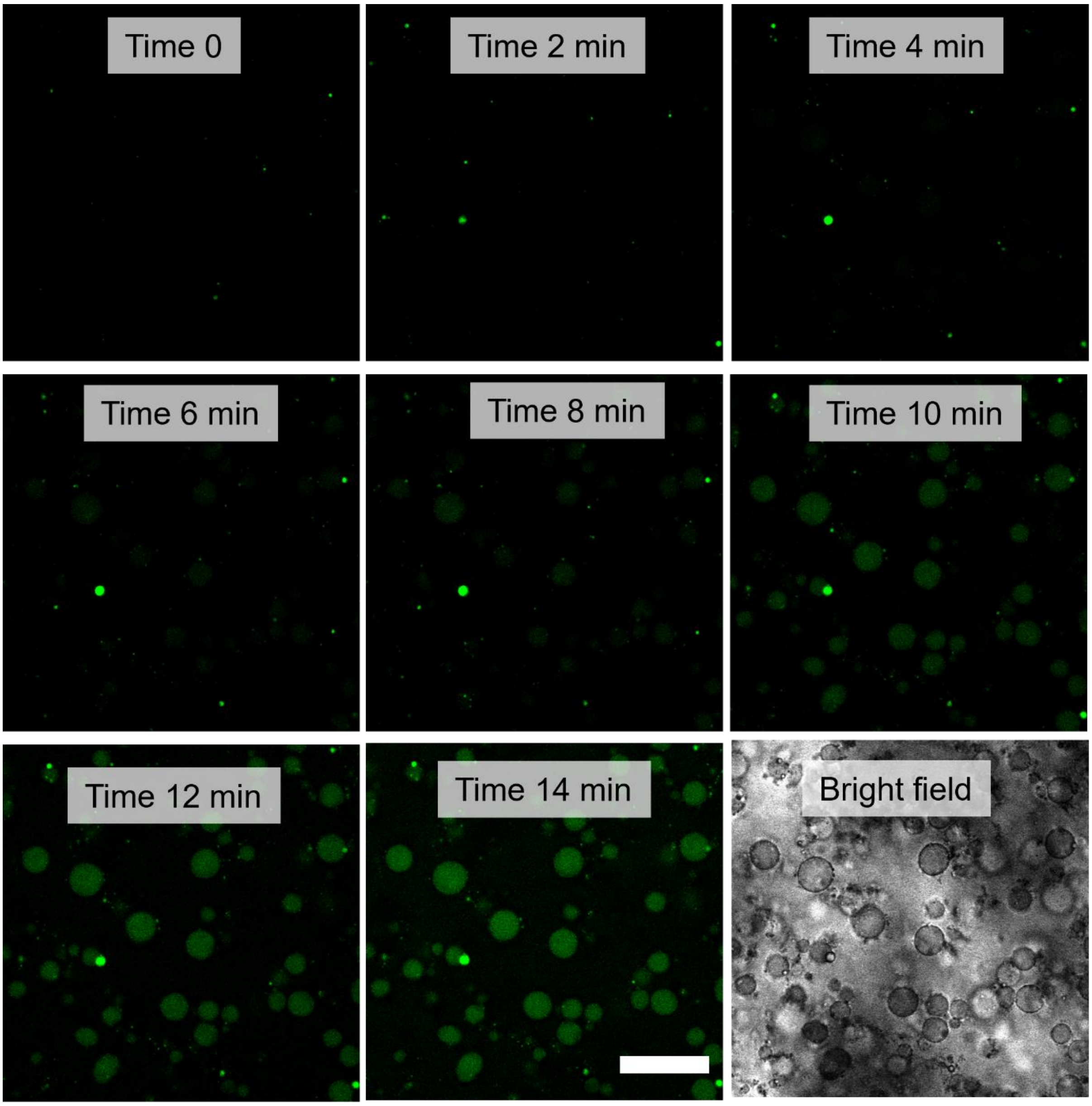

Supplementary Figure 77. Confocal images revealed that the presence of Ru catalyst and internal BPCs is necessary for effective catalytic activity in AND-MACs. The reaction involves the cleavage of allylcarbamates resulting in the activation of a pro-fluorophore, scale bar=20 μm in all images. Similar results were obtained with 3 samples measured independently.

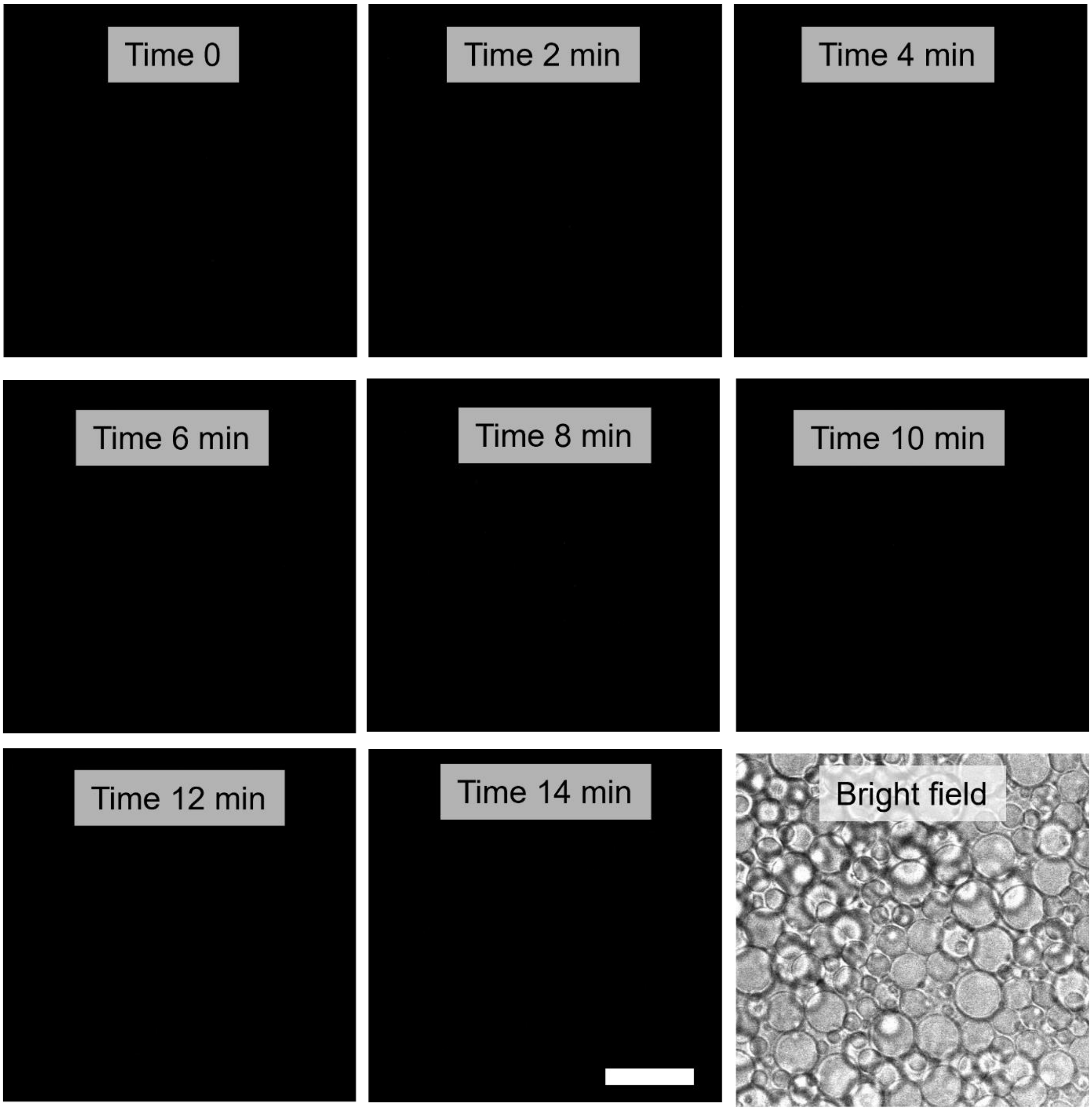

Supplementary Figure 78. Confocal images show that the presence of Ru-catalyst alone (no BPC) is not sufficient to initiate a catalytic reaction in AND-MACs. There is no obvious emission produced inside, scale bar=20 μm in all images. Similar results were obtained with 3 samples measured independently.

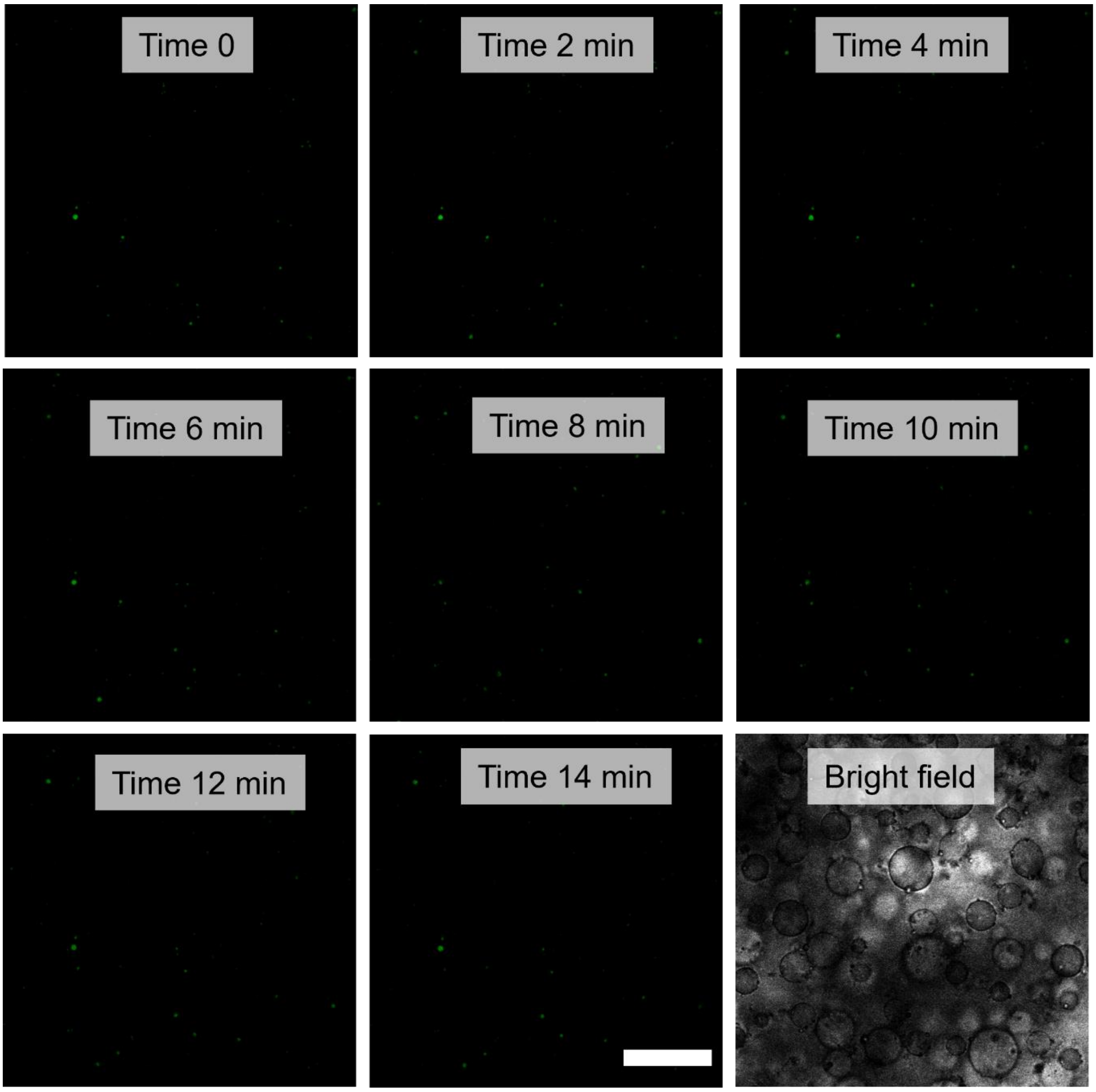

Supplementary Figure 79. Confocal images show that the presence of BPC alone (no catalyst) is not sufficient to initiate a catalytic reaction in AND-MACs. There is no obvious emission produced inside, scale bar=20 μm in all images. Similar results were obtained with 3 samples measured independently.

References:

1. Abbas, M., Lipinski, W. P., Nakashima, K. K., Huck, W. T. S., Spruijt, E. A short peptide synthon for liquid-liquid phase separation. *Nat. Chem.* **13**, 1046-1054 (2021).
2. Cao, S., Ivanov, T., Heuer, J., Ferguson, C. T. J., Landfester, K., Caire Da Silva, L. Dipeptide coacervates as artificial membraneless organelles for bioorthogonal catalysis. *Nat. Commun.* **15**, 39 (2024).
3. Gupta, A., Das, R., Yesilbag Tonga, G., Mizuhara, T., Rotello, V. M. Charge-switchable nanozymes for bioorthogonal imaging of biofilm-associated infections. *ACS Nano* **12**, 89-94 (2018).
4. Mason, A. F., Buddingh, B. C., Williams, D. S., Van Hest, J. C. M. Hierarchical self-assembly of a copolymer-stabilized coacervate protocell. *J. Am. Chem. Soc.* **139**, 17309-17312 (2017).
5. Pijpers, I. a. B.*, et al.* Hybrid biodegradable nanomotors through compartmentalized synthesis. *Nano Lett.* **20**, 4472-4480 (2020).
6. Van Der Spoel, D., Lindahl, E., Hess, B., Groenhof, G., Mark, A., Berendsen, H. Gromacs: Fast, flexible, and free. *J. Comput. Chem.* **26**, 1701-1718 (2005).
7. Huang, J., Mackerell Jr, A. D. Charmm36 all-atom additive protein force field: Validation based on comparison to nmr data. *J. Comput. Chem.* **34**, 2135-2145 (2013).
8. Yesylevskyy, S. O., Schäfer, L. V., Sengupta, D., Marrink, S. J. Polarizable water model for the coarse-grained martini force field. *PLoS Comput Biol* **6**, e1000810 (2010).
9. Hoover, W. G. Canonical dynamics: Equilibrium phase-space distributions. *Phys. Rev. A* **31**, 1695-1697 (1985).
10. Darden, T., York, D., Pedersen, L. Particle mesh ewald: An n* log (n) method for computing ewald sums. *J. Chem. Phys* **98**, 10089-10092 (1993).
11. Frederix, P. W. J. M.*, et al.* Exploring the sequence space for (tri-)peptide self-assembly to design and discover new hydrogels. *Nat. Chem.* **7**, 30-37 (2015).
12. Tang, Y.*, et al.* Prediction and characterization of liquid-liquid phase separation of minimalistic peptides. *Cell Reports Physical Science* **2**, 100579 (2021).
